# Nanoscale π-conjugated ladders

**DOI:** 10.1038/s41467-021-26688-9

**Published:** 2021-11-16

**Authors:** Stefanie A. Meißner, Theresa Eder, Tristan J. Keller, David A. Hofmeister, Sebastian Spicher, Stefan-S. Jester, Jan Vogelsang, Stefan Grimme, John M. Lupton, Sigurd Höger

**Affiliations:** 1 Kekulé-Institut für Organische Chemie und Biochemie der Universität Bonn, Gerhard-Domagk-Str. 1, 53121 Bonn, Germany; 2 Institut für Experimentelle und Angewandte Physik, Universität Regensburg, 93040 Regensburg, Germany; 3 Mulliken Center for Theoretical Chemistry, University of Bonn, Beringstr. 4, 53115 Bonn, Germany

**Keywords:** Conjugated polymers, Polymer synthesis, Single-molecule fluorescence

## Abstract

It is challenging to increase the rigidity of a macromolecule while maintaining solubility. Established strategies rely on templating by dendrons, or by encapsulation in macrocycles, and exploit supramolecular arrangements with limited robustness. Covalently bonded structures have entailed intramolecular coupling of units to resemble the structure of an alternating tread ladder with rungs composed of a covalent bond. We introduce a versatile concept of rigidification in which two rigid-rod polymer chains are repeatedly covalently associated along their contour by stiff molecular connectors. This approach yields almost perfect ladder structures with two well-defined π-conjugated rails and discretely spaced nanoscale rungs, easily visualized by scanning tunnelling microscopy. The enhancement of molecular rigidity is confirmed by the fluorescence depolarization dynamics and complemented by molecular-dynamics simulations. The covalent templating of the rods leads to self-rigidification that gives rise to intramolecular electronic coupling, enhancing excitonic coherence. The molecules are characterized by unprecedented excitonic mobility, giving rise to excitonic interactions on length scales exceeding 100 nm. Such interactions lead to deterministic single-photon emission from these giant rigid macromolecules, with potential implications for energy conversion in optoelectronic devices.

## Abstract (web summary)

Increasing the rigidity of a macromolecule while maintaining solubility is challenging. Here, the authors demonstrate covalent connection of two rigid-rod polymer chains with stiff connectors, leading to rigid ladder structures with well-defined conjugated rails.

## Introduction

**Fig. 1:**
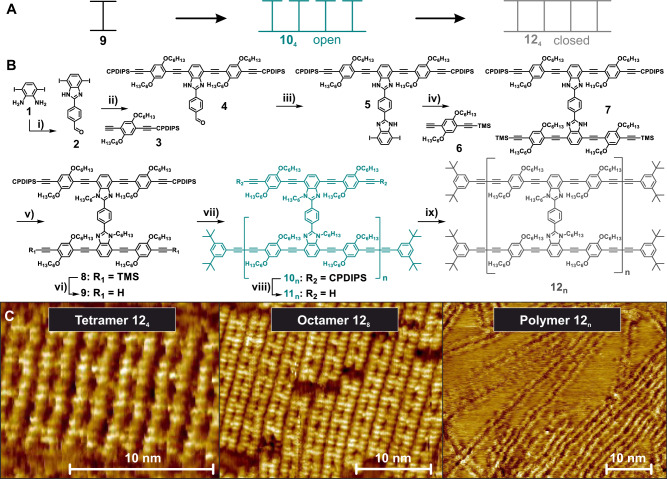
Oligomers and polymers of π-conjugated ladders. **A** Schematic representation of the ladder monomer **9**, and the open (**10**) and closed (**12**) oligomers. **B** The first steps of the synthesis by Sonogashira coupling involves the creation of “H”-shaped molecules (**1**–**9**). **9** is then used for polymerization by Glaser coupling, resulting in the oligomeric structures **10**_**n**_ with *n* = 4, 8, and ≈40 for tetramers, octamers and polymers. After removal of the CPDIPS-protecting groups, an intramolecular zipping reaction under Glaser coupling conditions leads to the ladder structures **12**_**n**_. i) Terephthalaldehyde, ZrCl_4_, CHCl_3_, r.t., 16 h, 49%, ii) **3**, PdCl_2_(PPh_3_)_2_, CuI, PPh_3_, THF, piperidine, r.t., 3 d, 55%; iii) **1**, ZrCl_4_, CHCl_3_, r.t., 15 h; iv) **6**, PdCl_2_(PPh_3_)_2_, CuI, PPh_3_, THF, piperidine, r.t., 2 d, 57% for two steps; v) 1-iodohexane, K_2_CO_3_, acetone, r.t., 2 d, 93%; vi) K_2_CO_3_, MeOH, CH_2_Cl_2_, 40 °C, 5 h, 99%; vii) oligomerization: 1) CuCl, CuCl_2_, CH_2_Cl_2_, pyridine, 25 °C, 3 h, 2) 3,5-di-*tert*-butyl phenylacetylene, 16 h, 4.5% (**10**_**4**_), 4.3% (**10**_**8**_); polymerization: 1) CuCl, CuCl_2_, CH_2_Cl_2_, pyridine, 25 °C, 16 h, 2) 3,5-di-*tert*-butyl phenylacetylene, 5 h, 2.8% (**10**_**n**_); viii) Bu_4_NF, CH_2_Cl_2_, THF, 35 °C, 1 to 3 h; ix) 1) CuCl, CuCl_2_, pyridine, 35 °C, 25 to 45 min, 2) 3,5-di-*tert*-butyl phenylacetylene, 15 to 17 h, 59% (**12**_**4**_), 54% (**12**_**8**_), 23% (**12**_**n**_) for two steps. (**C**) STM images of the ladder molecules **12** on graphite (image sizes: 14.9 × 12.0 nm^2^, 29.9 × 23.6 nm^2^, and 50.0 × 39.0 nm^2^).

The ladder is one of mankind’s oldest and most useful inventions—by some estimates twice the age of the wheel. While everyday ladders are much shorter than their “persistence length”—the distance over which correlations in the direction of the tangent are lost—they appear infinitely stiff to the user. Rope-ladders, on the other hand, are designed to be flexible so that they are easily rolled up to a compact snail. An example of a structure intermediate between these two extremes in terms of persistence length is the garden hose, which appears stiff only on short scales. A microscopic analogy of the latter is the rigid-rod polymer^1^, which is characterised by a spatial degree of shape persistence. Although denoted as stiff, such polymers have lengths far exceeding their persistence length^1,2^. Nature widely exploits ladder formation, often supramolecular as in DNA or collagen, for information storage or to enhance structural rigidity. The ladder structure has a higher shape persistence than the constituents of the ladder. Synthetic ladder-type molecular structures have been reported in many variants^3,4^, both noncovalent^5–8^ and covalent^9–12^. It has been shown that supramolecular “ladderization” can rigidify the structure, leading to an increase in the on-chain charge-carrier mobility and an enhancement of the two-photon absorption cross-section^13–15^. Although more robust to changes in environment, none of the covalently formed ladders reported to date actually appear to resemble a real-world ladder in structure, with well-defined discrete rigid rungs and continuous rigid rails, adjustable in spacing on the nanometre scale. In the context of π-conjugated macromolecules such a molecular design seems rather desirable. Each individual rail should exhibit signatures of a high degree of order in one dimension, i.e., minimal perturbation of the π-electron system, along with subtle influences arising from the presence of the second rail spaced at the—adjustable—constant distance of the length of the rung molecules. Interestingly, in the language of molecular spectroscopy, such a ladder molecule combines the two extremes: a perfect “J-aggregate,”^16^ named after Edwin Jelley, where all transition dipole moments of the individual π-conjugated units add up to form one larger unit; and an “H-aggregate,”^17^ referring to the hypsochromic shift resulting from the destructive interference of two cofacially positioned transition dipoles. In such a scheme, ladderization would solve two problems related to organic semiconductors at once^17^: it intramolecularizes interchain interactions in the bulk material to a certain degree since two conjugated wires align at exactly the same spacing with respect to each other, providing a fixed coupling between chains which would otherwise be random. And second, it is expected that ladderization enhances the persistence length of the entire molecular structure and thus of each individual conjugated rail, potentially raising the mobility of charge carriers and excitation energy. As in biological compounds, the system can then be viewed as self-rigidifying. Such rigidification of conjugated polymers can be observed in structures containing sterically demanding, e.g., dendritic side chains^18^, or by encapsulation of the main chain in a tube of macrocycles, either supramolecular^19^ or molecular^20,21^. However, in all these cases the conjugated backbone becomes isolated from the environment and therefore interchain interactions in the bulk and thus the flow of charge and excitation energy is impeded. Here, we introduce such covalently bonded ladder structures. Figure 1A illustrates the synthetic covalent zipping strategy to arrive at π-conjugated phenylene-ethynylene-butadiynylene ladder oligomers and polymers, where the “H”-shaped monomers^22^ representing the rungs and the individual rail segments of the ladder are polymerized.

## Results

### Synthetic procedure

The chemistry behind our synthesis is outlined in Fig. 1B. By condensation of **1** with two equivalents of terephthalaldehyde, the monocondensation product of the di-aldehyde is isolated. Twofold Sonogashira coupling of **2** with the 3-cyanopropyl-di-isopropyl (CPDIPS)-protected bisacetylene **3** containing additional solubilizing hexyloxy groups yields **4**, which is condensated with **1**. A further twofold Sonogashira coupling with **6** gives the H-shaped monomer **7** containing the two strands of the ladder: one with more labile trimethylsilyl (TMS)-protected acetylenes and one with stable CPDIPS-protected acetylenes. Deprotecting **7** at the TMS-protected position and using it as a monomer allows polymerization by Glaser coupling. However, workup and purification of the coupling products are challenging because of the high polarity of the compounds. Instead, the benzimidazole moieties of **7** are alkylated, which reduces the polarity of the “H”-shaped molecules and at the same time increases their solubility. Deprotection of **8** under mild conditions removes the TMS group while the CPDIPS group remains on the acetylenes. Oligomerization by oxidative homocoupling of the monomer **9** under Glaser coupling conditions is performed by stirring **9** in the catalyst mixture for a short time, and proceeds without uncontrolled cross-linking reactions. The progress of the reaction is monitored by gel permeation chromatography (GPC) and is terminated by end-capping of the products by the addition of a large excess of di-*tert*-butyl phenylacetylene. Oligomers **10**_**n**_ with only one of the ladder rails in place were isolated by recycling GPC. The CPDIPS-protecting groups were removed with Bu_4_NF to allow the intramolecular zipping reaction under Glaser coupling conditions to occur at high dilution and with a large excess of the catalyst. Subsequent end-capping, again with di-*tert*-butyl phenylacetylene, yields the ladder oligomers **12**_**n**_. Recycling GPC on **12**_**4**_ and **12**_**8**_ (Supplementary Fig. 33 and Supplementary Fig. 36) shows that only a small amount of by-product with higher molecular weight is formed, demonstrating that the intramolecular Glaser coupling is much faster than intermolecular cross-linking reactions. Extended reaction times for the Glaser coupling of **9** resulted in the formation of a polymer with a peak molecular weight *M*_*p*_ = 171 × 10^3^ g mol^−1^ and a dispersity *D* = 2.6. A narrowly dispersed polymer sample with *M*_*p*_ = 152 × 10^3^ g mol^−1^ and *D* = 1.1 was obtained by GPC separation. Since the mass of rigid-rod polymers is generally overestimated in polystyrene-calibrated GPC^23^, we corrected these values as described in Ref. ^19^ and estimate an average degree of polymerization of *n*~40 for the polymer fractions (Supplementary Fig. 18). The subsequent ladderization reaction of these fractions followed the protocol outlined above. The molecular weight of ~85.3 × 10^3^ g mol^−^^1^ arrived at following this method coincides remarkably well with the data from the MALDI-TOF measurements as discussed in Supplementary Note 4.3 and 4.4. Full details of the synthesis, purification and chemical analysis are provided in Supplementary Note 4, along with information on optical spectroscopy in Supplementary Note 1.1.

### STM imaging

The ladder structures of the product molecules are immediately visible in the scanning-tunnelling microscope (STM) images in Fig. 1C and in Supplementary Note 3. Within the linear ladders, rungs and rails with intramolecular distances of 2.3 nm and 1.2 nm, respectively, are clearly resolved for the tetramer and octamer (see Supplementary Fig. 6 and 7). **12**_**4**_ forms a 2D crystalline packing in which the rails align in parallel in a similar way to (single-stranded) hexyl-substituted arylene-alkynylenes^24^ and the H-shaped monomers^22^. Intermolecular rod-rod distances are defined by the hexyloxy chains that align along the HOPG main axis direction, thereby determining the growth direction of the lamella (see Supplementary Note 3.2). **12**_**8**_ forms a densely packed array, but with a reduced degree of order and overlap of the rails as compared to **12**_**4**_. The polymer **12**_**n**_ appears as an assembly of double-stranded structures in the image. While most polymer chains **12**_**n**_ are densely packed with neighbouring chains present along their entire length, others interact with adjacent molecules only at the ends of the rods. This pinning results in isolated longer segments, which interact only with solvent molecules and the HOPG surface but are still sufficiently rigid so as to not be deflected by the STM tip^25^. Interestingly, the ladder structures also allow the bridging of step edges of the graphite or of other ladder polymers (see Supplementary Fig. 8). Limiting factors on the STM image resolution are discussed in Supplementary Note 3.3.

### Molecular-dynamics simulations

**Fig. 2:**
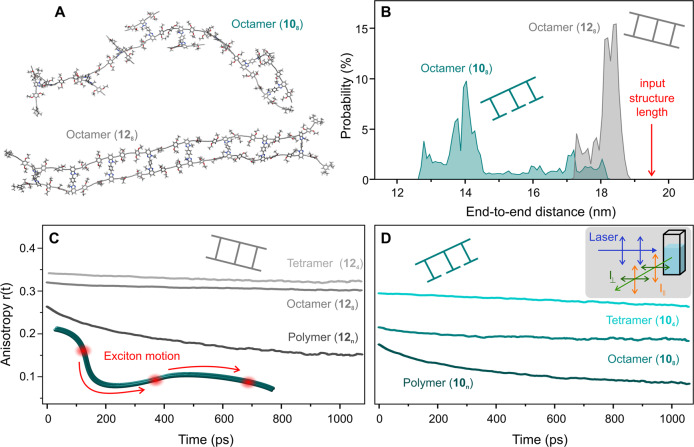
Shape persistence of π-conjugated ladders. **A** Representative structures computed from room temperature molecular-dynamics simulations of the open (**10**_**8**_) and closed (**12**_**8**_) octamer structure. **B** Radial distribution functions of the end-to-end distances of **10**_**8**_ and **12**_**8**_. The open octamer forms mostly bent conformations with an average length of 14.2 nm, whereas the closed structure yields a narrow distribution around 18.2 nm, close to the length of the input structure of 19.5 nm. **C** and **D** Transient fluorescence depolarization in toluene solution at room temperature, demonstrating the high level of rigidity of the ladder structures. Some depolarization arises in the polymers due to exciton motion along slightly curved chains.

While it is gratifying to visualize the ladders directly in real space for molecules deposited on a substrate, it is not obvious how rigidity is preserved in the condensed phase, or indeed in the native environment of a polymer film. We begin by exploring atomistic molecular-dynamics (MD) simulations using the recently developed “GFN” force field (GFN-FF)^26^. GFN-FF is an automated partially polarizable generic force field for the accurate description of structures and dynamics of large molecules of any atomic constituent, combining force-field speed with almost quantum-mechanical accuracy^27,28^. In contrast to most classical force-field and machine-learning potentials, GFN-FF performs a Hückel calculation for the conjugated pi-system to obtain the respective bond order. These calculations are employed to derive the bond bending and torsion force constants in order to properly compute the rigidity of the conjugated ladders. MD simulations were carried out for periods of 1 ns at room temperature (298 K), employing the implicit “GBSA” solvation model for tetrahydrofuran^29^. Further details of the computation are given in the Supplementary Note 2. Two representative room-temperature equilibrium molecular geometries are shown in Fig. 2A. The open octamer **10**_**8**_ is much more flexible and therefore less extended than the closed structure **12**_**8**_. Panel B shows the radial distribution functions of the end-to-end distances of **10**_**8**_ and **12**_**8**_ evaluated over the respective MD trajectories. Because of twisting and folding, **10**_**8**_ is, on average, compacted by almost 25% compared to the length of the optimized input structure of 19.5 nm. The molecular conformations scatter widely. In contrast, the distribution for **12**_**8**_ is much narrower and differs from the input by only 7%, attesting to the high degree of rigidity of the ladders. Supplementary Videos 1 and 2 show 100 ps segments of the evolution of the molecular dynamics for the open and closed octamer. Whereas the open octamer changes its conformation from straight to disordered over time, the ladder clearly remains rigid over the course of the molecular-dynamics simulation trajectory.

### Fluorescence polarization anisotropy

We probe this rigidity experimentally by examining the depolarization of the fluorescence in solution. Although this quantity is often reported for time-integrated fluorimetry^18^, it is more insightful to resolve it in time to be able to differentiate between the rotational diffusion and intramolecular energy-transfer phenomena, which occur on different timescales. The polarization anisotropy, a measure of the polarization memory, is defined as $r\left(t\right)=({I}_{\parallel }-G{I}_{\perp })/({I}_{\parallel }+2G{I}_{\perp })$, where $I$ indicates the fluorescence intensity, either parallel or orthogonal to the polarization plane of the excitation, and the factor $G$ accounts for the polarization sensitivity of the detection setup. A perfect dipole, randomly distributed in three dimensions, will yield an initial anisotropy of 0.4, which decreases with time because of rotational diffusion. The tetramer ladder **12**_**4**_, shown in Fig. 2C, indeed displays a very high polarization anisotropy of 0.35. The value is almost constant over the lifetime of the excited state of order 1 ns since the radius of gyration of the molecule is so large. A similar value of 0.32 is found for the octamer, implying that it is also almost perfectly extended. We note that the decrease in $r(t)$ over the lifetime of the excited state of the ladder octamer **12**_**8**_ is within the resolution of the measurement, implying that rotational diffusion, i.e. a depolarization due to motion of the molecule in solution, is unlikely to be of any relevance in the measurement. For the polymer, in contrast, a swift decay of $r(t)$ is found over the first 200 ps. These dynamics cannot be attributed to rotational diffusion given the size of the polymer chains, but are instead assigned to energy transfer^30^, i.e., motion of excitons, along the polymer backbone as indicated in the sketch. If the polymers were indeed perfectly extended, migration of excitons along the chain would have no effect on $r(t)$. However, evidently the ladder is not perfectly rigid and so $r(t)$ drops to 0.18 during the lifetime of the exciton. Such a depolarization would be reached if the entire chain acquires a curvature of some 37° overall, which, given the chain extension of order 100 nm, is a very small distortion for such a large molecule in solution.

As predicted by the molecular-dynamics simulations, the open structures **10**_***n***_ are much more flexible and show substantially lower $r(t)$ anisotropy values in panel D. The fact that the anisotropy for the open polymer even drops below 0.1 demonstrates that it acquires a three-dimensional conformation^31^. This is because a value close to zero is only possible if the polymer nanoparticle is capable of absorbing light in any arbitrary polarization plane and redistributing its excitation energy in the nanostructure^31–34^.

### Single-molecule fluorescence polarization modulation

**Fig. 3:**
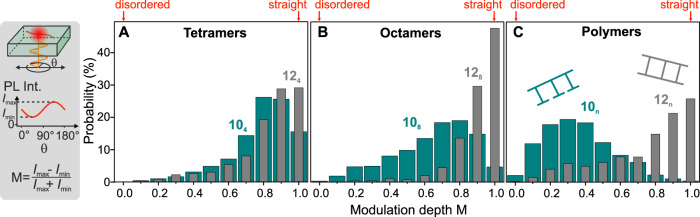
Molecular rigidity on the single-molecule level. Single molecules are embedded in an inert matrix and imaged with a fluorescence microscope, recording the intensity as the plane of polarization of the exciting laser is rotated. The fluorescence modulation depth M offers a metric to assess the rigidity of the chain. The histograms of M are shown for approximately 1000 single molecules each of the open and closed tetramers (**A**), octamers (**B**), and polymers (**C**).

Although it is reassuring to see that the rigidity of the ladder is retained in solution, at room temperature, the ensemble measurements tend to underestimate the overall degree of structural order because they average over many different conformations^31^. To assess the degree of order on the level of each individual ladder molecule, we employ single-molecule fluorescence spectroscopy. Recording the fluorescence intensity $I$ of single molecules diluted in a polymethylmethacrylate (PMMA) matrix as the plane of polarization of the incident light source is rotated provides an accurate measure for the degree of chain extension^32,35^. We define the modulation depth of $I$ as $M=({I}_{{{\max }}}-{I}_{{{\min }}})/({I}_{{{\max }}}+{I}_{{{\min }}})$, ranging from 0 for a random coil to 1 for a perfectly rigid rod^32^. Figure 3 compares ${M}$ values for many single molecules each of the single-stranded polymers **10**_**n**_ (green) and ladders **12**_**n**_ (grey). For the tetramer ladders, it is apparent that roughly twice as many single molecules, a total of almost 30%, are perfectly extended ($M=1$) when compared to the precursors. For the octamers, the difference between open and closed structures is much more dramatic: only 4% of the open structures show a modulation depth of $M=1$, whereas almost half of the closed ladders do.

It is not always trivial to relate the modulation depth measurements directly to molecular shape because tilting of the molecule within the focal plane of the microscope will also affect the polarization anisotropy. We previously reported extensively on this effect for conjugated shape-persistent macrocycles, for which all single molecules have exactly the same shape^36^. In such molecules, changes in the modulation depth relate directly to tilting in the focal plane. In the present case, the structural rigidity is likely to appear more pronounced for the octamer ladders than for the tetramers because the molecules may also tilt out of the plane of the PMMA matrix, reducing the apparent modulation depth—even if the ladder tetramers are actually straighter than the ladder octamers. Such tilting is more likely for the shorter molecules, which are more mobile in the matrix during spin coating, than for larger molecules, and gives rise to an underestimate of the rigidity. At the same time, of course, for flexible molecules the probability of folding or kinking of the chain increases with increasing chain length, lowering the observed modulation depth. On the other hand, flexible polymer chains may collapse into ordered rigid structures, leading to an increase in modulation depth with increasing chain length as discussed in Ref. ^32^. This is clearly not the case here: for the polymer in panel C, none of the open ladder structures show complete fluorescence modulation, with the distribution of $M$ values being broad and peaking at around 0.3, close to the value expected for an isotropic polymer nanoparticle^35^. In stark contrast, 25% of the closed ladders are perfectly straight, with $M=1$, and 62% have $M > 0.8$. However, 16% of all ladder molecules show $M < 0.5$, implying a substantial degree of disorder and suggesting that a subset of the molecules are bent within the matrix. Qualitatively, these experimental histograms of the fluorescence modulation depth offer an appealing microscopic analogue for the chain extension measured in the molecular-dynamics simulations in Fig. 2B: the histograms for **12**_**8**_ in Fig. 3B are much narrower than those for **10**_**8**_.

It is conceivable that, in rare events, topological defects may arise during ladderization by incomplete zipping^35^, compromising overall rigidity due to the presence of a single-strand segment. An example of such a rare event, where the zipping reaction failed at the position of one rung, is shown in Supplementary Fig. 7B and discussed in detail in Supplementary Note 3.2.2. With this information, it can be argued that the histogram of the modulation depth values of the ladder polymer **12**_**n**_ resembles a bimodal distribution, with the distribution of low $M$ values closely matching the distribution found for the open polymers **10**_**n**_ (see Supplementary Fig. 4 and Supplementary Note 1.2.4). Such single topological defects are well known from ladder-type poly(*para*-phenylene), where they give rise to distinct spectrally separable chromophores with different dipole orientations within one polymer chain^37^.

### Single-molecule photon-correlation spectroscopy

**Fig. 4:**
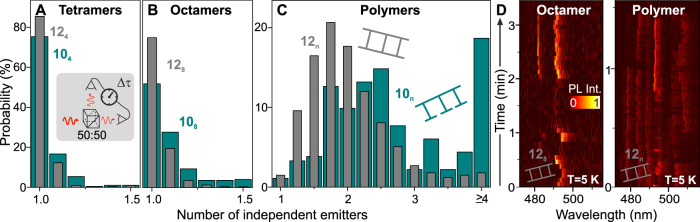
Number of independent emitting units in open (green) and closed (grey) ladder structures. **A–C** The number of emitters can be estimated from the temporal correlation of the fluorescence intensity, by passing the emitted light through a beam splitter and recording the photon coincidence rate on two photodetectors as a function of delay time Δτ. Histograms are shown for approximately 200 single molecules each, demonstrating that single ladder polymers emit fewer than two photons at once even though they contain two separate conjugated strands. **D** At 5 K, exciton mobility along the chain is reduced, so that individual optical transitions occur at distinct wavelengths. The octamer ladder displays two optical transitions as would be expected from the two conjugated strands. In the polymer ladder, up to six distinct optical transitions are identified, which can be assigned to independent emitters. This increase in the number of emitting units arises because the conjugation along each strand becomes disrupted when the molecule is frozen in the matrix.

The degree of order of the ladder structure will also affect the photophysics of the molecule. Whereas the open structures **10**_**n**_ contain only one extended conjugated chromophore, the closed ladders **12**_**n**_ comprise two. Each segment of a conjugated chain can, in principle, serve as a chromophore, a source of light emission. Interruptions of the conjugation will increase the number of effective chromophores in the polymer^38^. However, the chromophores can also interact with each other through a mechanism whereby the excited state of one unit absorbs the excitation energy of another^39^. This “singlet-singlet annihilation” process depends on the mobility of excitons within the molecule, and can lead to multichromophoric systems deterministically emitting precisely one photon at a time^39^. This effect can be probed by measuring the correlation of the fluorescence intensity of one single molecule. As sketched in Fig. 4A, the luminescence is passed through a beam splitter, and the rate of photon coincidence on two photodetectors is recorded. If the molecule emits only one photon at once, the correlation amplitude between the two detectors will be zero at zero delay time $\Delta \tau$ between the two detectors. As described in detail in Supplementary Note 1.2.3 and presented in Ref. ^40^, this information on “photon antibunching” can be translated into an effective number of independent emitters present within the molecule by considering the ratio between photon coincidence rates at zero and finite delay times. This mathematical translation procedure depends sensitively on the signal-to-background intensity level in the fluorescence measurement, as outlined in Supplementary Fig. 3. In brief, the ratio will tend to 0 for infinitely high signal-to-background level for one single chromophore, 0.5 for two, 0.67 for three, and so on. Figure 4A demonstrates that most open tetramer molecules indeed emit precisely one single photon at a time. The ladder structures have a slightly higher fidelity of such photon antibunching. Even though the ladder molecules contain two chromophores—the two conjugated rails—they still act as sources of single photons. The difference between open and closed octamers is more substantial as seen in panel B, but here, again, 75% of the single ladder molecules are perfect single-photon emitters. For the polymers shown in panel C the difference is quite dramatic. Here, over 57% of the ladder structures emit fewer than two photons at a time, whereas almost 20% of the open polymers have four or more independent emitters. It is perhaps surprising that the behaviour of **10**_**n**_ appears to be different from that of **10**_**8**_, with the polymer accommodating many more chromophores, but this is rather expected given the fact that the polymer is, on average, roughly five times as long as the octamer. In addition, chain folding may increase upon embedding the molecule in the PMMA matrix, which can cause even more breaks in conjugation to form with an increase in the number of apparent chromophores^34,38^. Furthermore, because the polymer molecule is more extended in space than the octamer, the transfer of excitation energy to only one emitting chromophore at a given time, an effect well studied in single polymer chains^35,41^, is not as probable in **10**_**n**_ as it is in **10**_**8**_.

The fact that single ladder polymer molecules **12**_**n**_ exist that effectively behave as one single independent emitter demonstrates that singlet-singlet annihilation occurs both along each entire chain and between the two parallel chains^42^. This is a remarkable observation given the fact that the polymers are, on average, roughly 100 nm in length and the size of the primary exciton is of order 1 nm. Each exciton on each conjugated rail therefore explores the entire span of the polymer chain so that exciton annihilation occurs. Such exciton motion along the chain also results in the gradual loss of polarization anisotropy in **12**_**n**_ in the time-resolved ensemble spectroscopy in Fig. 2C. Typically, the exciton diffusion length in π-conjugated ladder-type polymers is limited to some 10 nm^43^. Only in the unique compound of *trans*-polydiacetylene polymerized in its monomeric crystal has ballistic exciton migration been found to occur over distances of several microns^44^. We propose that a small subset of our ladder polymers is sufficiently rigid to behave like such almost perfect one-dimensional quantum wires, in analogy to crystalline polydiacetylenes^45^.

### Cryogenic single-molecule fluorescence spectroscopy

The singlet-singlet annihilation responsible for photon antibunching in the double-stranded ladder structures can be suppressed by reducing the overlap between the emission spectrum of a “donor” chromophore and the absorption of an “acceptor”^41^, where the acceptor is the excited state of a second chromophore^40^. Such a suppression can arise when the extrinsic electronic disorder along the π-electron system induced, for example, due to interactions with the amorphous environment, exceeds the intrinsic spectral width of the transition. A simple way to probe this condition is to cool the sample down to cryogenic temperatures. Figure 4D shows the temporal evolution of the fluorescence spectrum of single molecules of **12**_**8**_ and **12**_**n**_ at a temperature of 5 K. In contrast to the results from photon-correlation spectroscopy obtained at room temperature (panels A–C), the fluorescence of the octamer at 5 K clearly shows two distinct transitions, corresponding to the two conjugated rails. The spectra change in peak position and intensity with time, manifesting spectral diffusion and blinking due to interactions with the environment^46^. Less intense features are apparent in the fluorescence spectrum due to the discrete vibronic progression^47^. The spectrum of the ladder polymer shows up to six distinct peaks, corresponding to simultaneously emitting spectrally distinct chromophores^39^. We conclude that, at these low temperatures, excitonic motion along the ladder rails is inhibited, as is the singlet-singlet annihilation effect. The subtle disorder in the ladder structure serves to localize the emission centres within the molecule^41^, thereby providing a direct fingerprint of the overall structural complexity of the molecule in luminescence.

## Discussion

We have demonstrated a versatile synthetic strategy to form highly conjugated ladder polymers of unparalleled dimensions. In a conventional ladder, the rails serve to fixate the rungs a constant distance apart. In our molecular ladders, the rungs separate the rails, preventing the polymer from collapsing. This concept is quite generic and also applies to macrocycles, which are turned into “spoked wheel” structures by affixing rungs^48,49^. Unlike conventional approaches to rigidification, which rely on dendronization^18^ or encapsulation^19,20^, the polymer strands here retain their native environment, implying that they can be employed in optoelectronic applications such as for exciton and charge transport in photovoltaic or light-emitting devices. Given the high degree of control over structural rigidity and electronic ordering, we also anticipate applications in quantum optics: with an extension of the double-stranded ladder polymer of some 100 nm, this structure appears to constitute the longest deterministic source of single photons reported to date.

## Methods

The synthetic methods and the detailed characterisation of the materials are described in the Supplementary Material, as are the spectroscopic methodology, the STM technique and the molecular-dynamics simulations.

## Supplementary information

Supplementary Information

Description of Additional Supplementary Files

Supplementary Movie 1

Supplementary Movie 2

## Data Availability

The raw data that support the plots within this paper and the other findings of this study are available from the corresponding author upon reasonable request.
