# Supplementary material for: Nanoscale π-conjugated ladders
Source: Nat Commun. 2021 Nov 16;12:6614. doi: 10.1038/s41467-021-26688-9 (PMC8595307; doi:10.1038/s41467-021-26688-9)
Supplement: Supplementary file 1 — Supplementary Information [file 41467_2021_26688_MOESM1_ESM.pdf]

Supplementary Information

Nanoscale π-conjugated ladders

Stefanie Meißner[1], Theresa Eder[2], Tristan J. Keller[1], David A. Hofmeister[1], Sebastian Spicher[3], Stefan-S. Jester[1], Jan Vogelsang[2], Stefan Grimme[3], John M. Lupton[2,*)], Sigurd Höger[1,*)]

[1]Kekulé-Institut für Organische Chemie und Biochemie der Universität Bonn

Gerhard-Domagk-Str. 1, 53121 Bonn, Germany

[2]Institut für Experimentelle und Angewandte Physik, Universität Regensburg

93040 Regensburg, Germany

[3]Mulliken Center for Theoretical Chemistry, University of Bonn

Beringstr. 4, 53115 Bonn, Germany

[*)] Corresponding authors. john.lupton@ur.de, hoeger@uni-bonn.de

Contents

Supplementary Notes, Figures and References

**Supplementary Notes 1: Photophysics**	**3**
**Supplementary Notes 1.1: Ensemble measurements**	**3**
**Supplementary Note 1.1.1: Absorption and emission spectroscopy**	**3**
**Supplementary Note 1.1.2: Transient fluorescence depolarization spectroscopy**	**4**
**Supplementary Notes 1.2: Room-temperature single-molecule spectroscopy**	**5**
**Supplementary Note 1.2.1: Sample preparation and setup**	**5**
**Supplementary Note 1.2.2: Excitation polarization modulation depth**	**6**
**Supplementary Note 1.2.3: Single-molecule photon-correlation spectroscopy**	**7**
**Supplementary Note 1.2.4: Bimodal distribution of polarization modulation-depth histogram**	**9**
**Supplementary Note 1.3: Cryogenic single-molecule spectroscopy: sample preparation and setup**	**9**
**Supplementary Note 2: Computational details - GFN-FF**	**10**
**Supplementary Notes 3: Scanning tunnelling microscopy**	**11**
**Supplementary Note 3.1: Experimental setup and methods**	**11**
**Supplementary Notes 3.2: Additional scanning-tunnelling microscopy images**	**11**
**Supplementary Note 3.2.1: Molecular structure and ordering of 12_4**	**11**
**Supplementary Note 3.2.2: Molecular structure and ordering of 12_8, and identification of defects**	**12**
**Supplementary Note 3.2.3: Molecular structure and ordering of 12_n, and identification of overlapping chains**	**13**
**Supplementary Note 3.3: Comment on STM image resolution and visibility of defects**	**15**
**Supplementary Notes 4: Synthesis**	**16**
**Supplementary Note 4.1: Materials and equipment**	**16**
**Supplementary Note 4.2: Synthesis of starting materials**	**18**
**Supplementary Note 4.3: Oligomerization**	**28**
**Supplementary Note 4.4: Polymerization**	**48**
**Supplementary References**	**55**

Supplementary Notes

Supplementary Notes 1: Photophysics

Supplementary Notes 1.1: Ensemble measurements

Supplementary Note 1.1.1: Absorption and emission spectroscopy

To perform ensemble measurements in solution, the analytes were dissolved in toluene and measured in 10-mm quartz cuvettes (Quartz SUPRASIL® from Hellma analytics). The absorption spectra in Supplementary Fig. 1 were recorded using a Perkin Elmer spectrometer (Lambda 650). To obtain the corresponding emission spectra, we used a Horiba Jobin-Yvon Fluoromax 4 fluorescence spectrometer at an excitation wavelength of 440 nm.

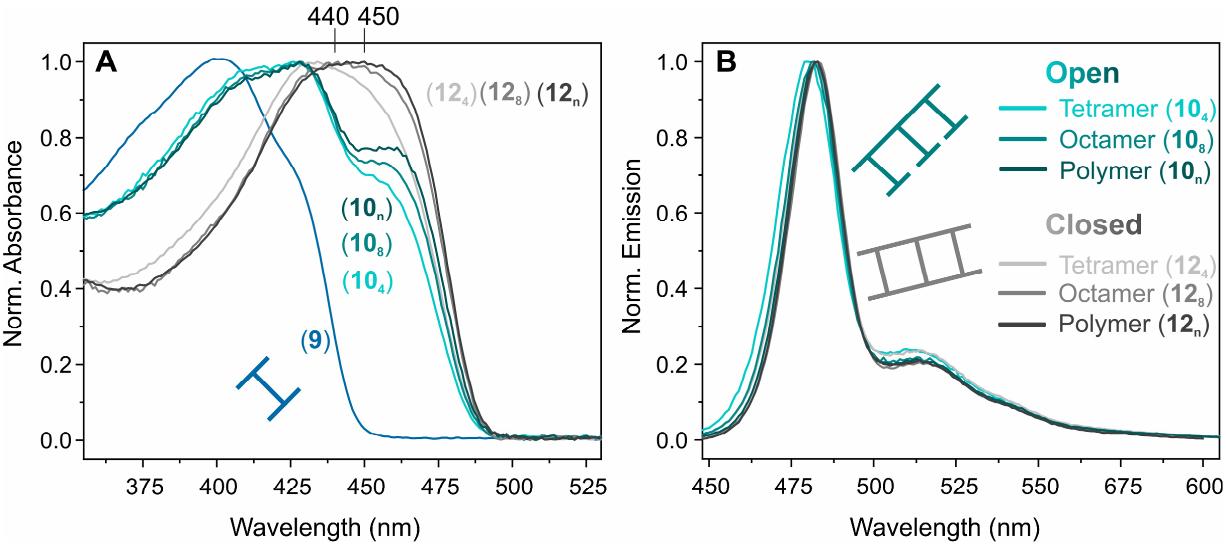

Supplementary Figure 1. Ensemble absorption and emission spectroscopy of the different compounds in toluene solution. (A) The absorption spectra vary depending on the type (open or closed) of the molecule. In closed formations (grey) an increased absorption is observed at higher wavelengths above 450 nm, which can be assigned to the larger conjugated parts of the molecules. This feature is completely absent in the monomer **9** (blue). The open formations (cyan), however, exhibit a stronger absorbance at wavelengths below 450 nm. This feature can be assigned to absorption of the open monomeric units and the linking segments, the rungs of the ladder. Additionally, absorption of the longer conjugated backbone unit can be seen at wavelengths above 450 nm, but this absorption is significantly reduced compared to the closed ladder formations. All absorption measurements were performed with a low absorbance of <0.06 at 440 nm to avoid inner filter effects. **(B)** The PL emission spectra are virtually identical for all larger molecules (**12_8, 10_n** and **12_n**). The luminescence of the tetramers (**10_4** and **12_4**) is more blue-shifted due to

their shorter conjugation length. Upon close inspection, one can discern a slight blue shift of the open octamer **10_8** as well, suggestive of slight bending, which may decrease the effective conjugation length. Also notable is the slight difference in the vibronic contribution between the system containing four repeat units and the larger systems, providing further evidence for a greater conjugation length in the latter. The fact that emission spectra are virtually identical for both open and closed formations points to efficient excitation energy transfer to the longer conjugated segments of the molecules.

Note that, at first inspection, conjugation in the connecting segments between the ladder strands may appear feasible. However, the central phenylene is expected to twist out of the plane of conjugation because of interactions with the NC_6H_{13} groups on either side. There is no indication of an electronic delocalization effect due to conjugation through the ladder rungs in either absorption or fluorescence. Comparison of the absorption spectrum of the individual "rung" monomer **9** to the rail oligomers in Supplementary Fig. 1 shows that there is an absorption feature in this spectral region for all systems under consideration. However, since this absorption is of high energy, one would expect energy transfer to larger conjugated parts of the molecule to prevent emission from these rung segments, and indeed we see no evidence for such emission. However, we do have preliminary evidence (not shown) that the single-molecule excitation polarization modulation depth decreases for shorter excitation wavelengths.

Supplementary Note 1.1.2: Transient fluorescence depolarization spectroscopy

To measure the transient fluorescence depolarization shown in Figure 2 of the main text, the so-called L-format method with a single emission channel is used[1], as sketched in Supplementary Fig. 2. The analyte is excited by a frequency-doubled Ti:sapphire femtosecond laser system (Chameleon Ultra II, Coherent Inc., and HarmoniXX, APE GmbH), operating at 450 nm. To determine the polarization sensitivity of the setup, a half-wave plate allows to switch between vertically and horizontally polarized excitation. The emission is detected with a Hamamatsu streak-camera system consisting of a spectrograph (Bruker 520IS, Bruker Corporation), a streaking unit (C5680, Hamamatsu Corporation), and an ORCA-ER CCD camera (C4742-95, Hamamatsu Corporation). By measuring vertically ($I_V(t)$) and horizontally polarized ($I_H(t)$) emission components upon vertically polarized excitation, the time resolved anisotropy r(t) is calculated as shown in Supplementary Fig. 2. The vertically and horizontally polarized emission can also be referred to as $I_{||}$ and $I_{\perp}$ since it is parallel respectively perpendicular to the vertically polarized excitation. To correct for the polarization sensitivity of the detection setup, vertically and horizontally polarized emission components were also detected under horizontally polarized laser excitation. With these two

measurements, a correction factor $G=I_V/I_H$ is introduced to correct for the systematic polarization sensitivity of the setup.

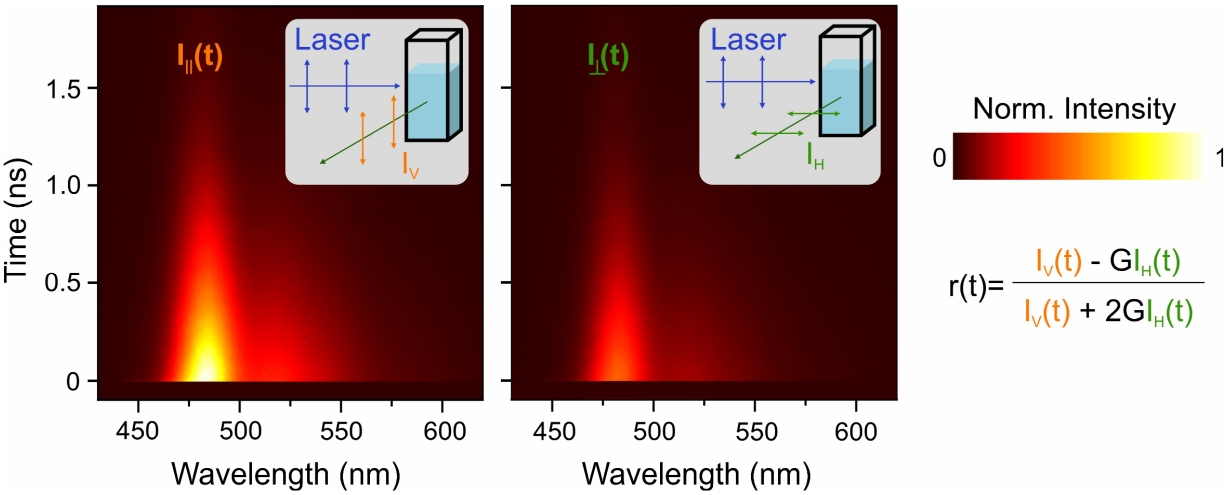

Supplementary Figure 2. Time-resolved fluorescence depolarization spectroscopy. Examples of streak-camera images of the closed octamer $\mathbf{12_8}$ for detection of vertically (I_V) and horizontally (I_H) polarized fluorescence components following vertically polarized excitation. After determining the correction factor G by exciting the solution with horizontally polarized radiation, the time-dependent polarization anisotropy r(t) can be calculated from these images as stated.

Supplementary Notes 1.2: Room-temperature single-molecule spectroscopy

Supplementary Note 1.2.1: Sample preparation and setup

To perform single-molecule measurements, the analyte molecules were embedded in a non-fluorescent poly(methyl-methacrylate) (PMMA, M_n=46 kDa, PDI=2.2 from Sigma Aldrich Co.) host matrix. To this end, the following steps were carried out. First, borosilicate glass cover slips were sonicated (Elma, Elmasonic P) in a 2 % Hellmanex III (Hellma Analytics) solution, followed by rinsing with ultrapure MilliQ water. Second, the cover slips were transferred into a UV-ozone cleaner (Novoscan, PSD Pro Series UV) to bleach the glass coverslips from residual fluorescent molecules. Third, the analyte was diluted in toluene to single-molecule concentration (~10^{-12} M) and mixed with a 2 % PMMA/toluene solution. Fourth, this solution was then dynamically spin coated (Laurell Technologies Corporation, Model WS-400BZ-6NPP/LITE) at 2000 r.p.m. in a glovebox under dry-nitrogen atmosphere onto the glass cover slips, resulting in a film thickness of roughly 100 nm. Aside from the low-temperature data in Figure 4, all measurements in the main text as well as for the supporting data were performed under ambient conditions so as to quench triplet states

which limit the single-molecule fluorescence yield. An inverted microscope (Olympus IX71) was used for wide-field as well as for confocal excitation of the samples, and for fluorescence detection.

Supplementary Note 1.2.2: Excitation polarization modulation depth

To perform the excitation polarization measurements, a fibre-coupled diode laser (PicoQuant LDH-C-440) with a wavelength of 440 nm was operated in continuous mode and the excitation light was passed through a clean-up filter (AHF analysis technology, HC Laser Clean-up MaxDiode 445/10). Subsequently, the linearly polarized excitation laser beam was rotated with an electro-optical modulator (FastPulse Technology Inc., 3079-4PW) and an additional λ/4-wave plate as described elsewhere[2]. The laser beam was expanded and focused via a lens system onto the back focal plane of the oil-immersion objective (Olympus, UPLSAPO 60OX, NA = 1.35) through the back port and a dichroic mirror (AHF analysis technology, RDC 442 nt) in the microscope. An excitation area of ≈ 80 × 80 μm^2 was generated in the focal plane and the fluorescence of the sample was collected by the same objective, subsequently passing through the dichroic mirror onto an EMCCD camera (Andor iXON3 897). The excitation intensity was set to ≈ 500 mW/cm^2 and the overall magnification resulted in a spatial resolution of approximately 160 nm^2 per pixel, leading to diffraction-limited spots of ≈ 2 × 2 pixels for a single molecule. The polarization was rotated by 180° over periods of 20 s and the fluorescence intensity of each spot was recorded as a function of the polarization angle. A total spot size of ≈ 5 × 5 pixels was assumed to calculate the overall intensity and the local background of the surrounding area was subtracted for each molecule. The data analysis was conducted with a customized software in MATLAB[3]. During data evaluation, it became clear that the measured modulation depth is slightly dependent on the plane of polarization of the molecular absorption, i.e. on the absolute orientation of the transition dipole moment. Such an effect can be caused by a slight polarization artefact of the setup, most probably induced by the dichroic mirror installed, and effectively decreases the apparent modulation depth measured. Such an artefact only becomes apparent in highly polarized absorbers. We therefore sorted the molecules with respect to their absolute phase angle in the modulation experiments, i.e. with respect to their orientation in the laboratory frame, and discarded those single molecules from further evaluation, which were orientated along the less-sensitive detection direction of the setup. This selection procedure was performed analogously for all samples to ensure comparability between the different datasets.

Supplementary Note 1.2.3: Single-molecule photon-correlation spectroscopy

For this measurement, the samples were excited by a frequency-doubled Ti:sapphire oscillator (Spectra Physics MaiTai BB) with laser pulses of approximately 80 fs duration and a repetition rate of 80 MHz. The wavelength was set to 440 nm and the beam was expanded to a diameter of ~1 cm with a lens system before being coupled into the oil-immersion objective. An excitation intensity of ≈ 200 W/cm^2 was used for the tetramers (**10_4**, **12_4**) and octamers (**10_8**, **12_8**) and an intensity of ≈ 40 W/cm^2 for the polymers (**10_n**, **12_n**). To obtain fluorescence images, an area of 20 × 20 µm^2 was scanned using a piezo stage (Physik Instrumente (PI) GmbH & Co. KG). Using the piezo stage and the fluorescence images, the laser beam was focused on one single molecule at a time and its emission was collected by the objective and subsequently spatially and spectrally filtered by a 50 µm pinhole and a fluorescence filter (AHF Analysentechnik AG, Edge basic LP 442 long-pass filter). The filtered fluorescence was split with a 50:50 beam splitter into two equivalent detection channels. Two avalanche photodiodes (APD) from PicoQuant (τ-SPAD-20), connected to a time-correlated single-photon counting module (TCSPC, PicoQuant GmbH, HydraHarp 400) were used as detectors. A Hanbury Brown and Twiss[4] arrangement was used to measure the time difference $\Delta\tau$ between photon arrival times. The TCSPC data was correlated and evaluated using a LabView program. An example histogram of correlation events for a closed octamer **12_8** is shown in Supplementary Fig. 3. The central peak counts the occurrence of photons recorded on both detectors after one laser pulse whereas the lateral peaks count how often there is a difference of one, two or three laser pulse periods between incident photons. Following Ref. (5), the central and mean lateral values of the photon coincidence histogram can then be used to calculate the number of independent emitters as shown in Supplementary Fig. 3. For the histogram in Figure 4 of the main text, this analysis was performed for each molecule individually. To correct for the background, the signal-to-background ratio S/B for each molecule was determined from the fluctuations in the intensity time traces, and a theoretical limit for the number of emitters present was calculated as explained in detail in Ref. 5. As the S/B ratio ranges between 75 and 600 for all single molecules, the theoretical minimum of the number of emitters extracted from the photon antibunching dip in the correlation is below 1.05 for the case of one emitter and below 2.11 for the case of two emitters as shown in Supplementary Fig. 3.

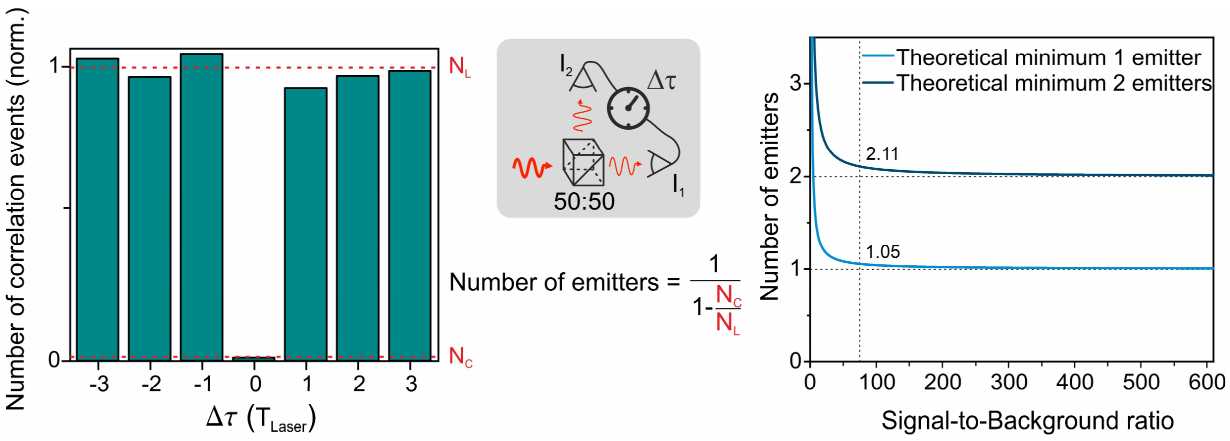

Supplementary Figure 3. Photon antibunching measurement to determine the number of independent emitters in a molecule. Left: an example of a photon correlation histogram obtained for one single closed octamer molecule **12**$_8$, as extracted from the PL intensity trace. The correlation events for each shift in time Δτ between two laser pulses in intervals of the laser period T_{Laser} = 12.5 ns are counted, and the resulting histogram is normalized to the mean value of occurrences on the lateral peaks N_L. This value describes the amplitude of correlations for photon arrival times with a time lag larger than one excitation pulse period. The probability of measuring two photons at the two detectors for one and the same laser pulse is given by the central peak value N_C, which is 0.011 for the example of a closed octamer molecule depicted. The central-to-lateral peak value ratio, N_C/N_L, can be evaluated for each molecule measured individually, as was done in Figure 4 of the main text. For each individual ratio N_C/N_L, the number of independent emitting units can be computed. To correct for the influence of the fluorescence background in the measurement of the photon correlation, the signal-to-background (S/B) ratio of the measurement must be accounted for. This value ranges between 75 for dark molecules and 600 for bright ones. Following the analysis in Ref. 6, the theoretical minimum of N_C/N_L, and therefore the effective number of independent emitters, can be calculated as shown on the right-hand side of the figure. For dark molecules with a low S/B ratio of 75, the number of emitters determined from the photon antibunching dip rises from 1 to 1.05 for the case of one single emitter present, and from 2 to 2.11 for the case of two. In bright molecules with a large S/B ratio these values converge to 1 and 2, respectively.

Supplementary Note 1.2.4: Bimodal distribution of polarization modulation-depth histogram

We note that the distribution histogram of polarization modulation-depth values for the ladder polymer **12_n** contains a substantial number of low values. Interestingly, the statistical distribution in this low-value range appears to closely match the distribution observed for the open polymer structures **10_n**, as shown in Supplementary Fig. 4. We proposed that the distribution for **12_n** is bimodal in nature and that the distribution in the range of low values arises from polymers for which a defect is present so that a kink occurs in the chain. An example of such a defect is discussed in more detail in the STM image in Section 3.3.2. Such a kinked closed polymer structure would then tend to represent the open polymer **10_n** conformation.

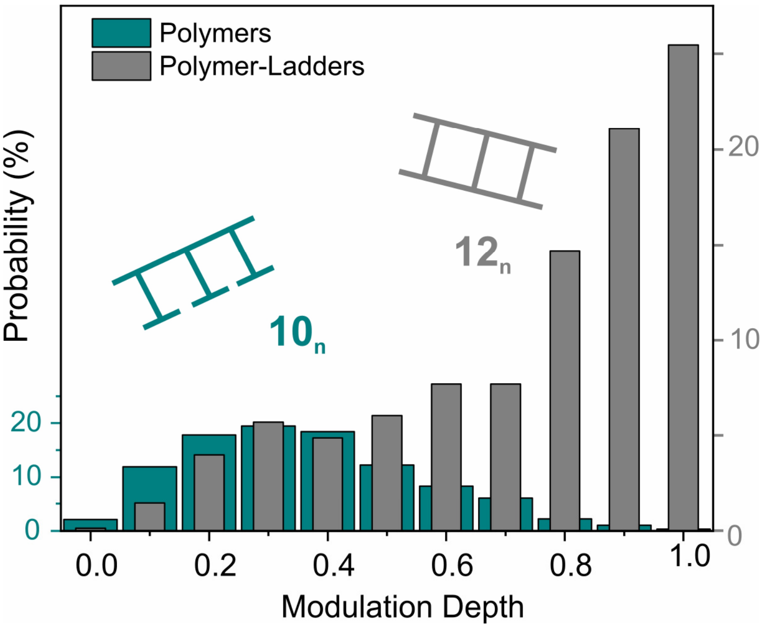

Supplementary Figure 4. Bimodal distribution of modulation depth values of the closed ladder polymers 12_n. The distribution closely matches that of the open polymers **10_n** in the range of low values.

Supplementary Note 1.3: Cryogenic single-molecule spectroscopy: sample preparation and setup

To measure the spectra at cryogenic temperatures shown in Fig. 4 of the main text, the analyte molecule samples were prepared as explained in section 1.2 except that sapphire glass was used instead of borosilicate glass for the cover slips. The substrate was mounted in a cold-finger helium-flow cryostat and cooled to a temperature of approximately 5 K. Excitation of the sample was carried out by a frequency-doubled Ti:sapphire femtosecond laser system (Chameleon Ultra II, Coherent Inc., and HarmoniXX, APE GmbH), operating at 440 nm. The laser was focused on the back focal plane of the objective (Olympus, LUCPLFLN40X) with a lens, resulting in parallel illumination of around 70 × 70 µm² on the sample. The red-shifted fluorescence was subsequently transmitted through a dichroic mirror (AHF Analysentechnik, RDC

442 nt) onto a sCMOS-Camera (ORCA-Flash), which allows the inspection of all emitting molecules in the illuminated area. A fluorescence filter (AHF Analysentechnik AG, Edge basic LP 442 long-pass filter) blocks scattered laser radiation. The fluorescence of selected spots could then be focused on the entrance slit of a grating spectrometer (Princeton Instruments, Acton SP2300) with a lens. A grating of 600 grooves/mm was used to disperse the signal before the spectrum was recorded with a cooled CCD camera (Princeton Instruments, PIXIS 100). An integration time of 2 s or 1 s was used for the octamers and polymers, respectively, at an excitation power of approximately 800 W/cm^2.

Supplementary Note 2: Computational details - GFN-FF

For the input structures, geometry optimizations were performed at the GFN-FF[7] level of theory. The resulting structures served as input for subsequent molecular dynamics (MD) simulations. MD simulations with GFN-FF were carried out for 1 ns at room temperature (298 K) employing the implicit GBSA (THF) solvation model. A time step of 2 fs at an increased hydrogen mass of 4 amu and an equilibration phase of 200 ps was chosen. All calculations were performed with the freely available xtb 6.3.2[8] (GFN1-xTB[9], GFN2xTB[10], GFN-FF[7]) program packages with default convergence criteria $10^{-7}\,E_h$ for energies and $10^{-5}E_h \cdot \mathrm{Bohr}^{-1}$ for gradients. All calculations were performed on Intel© Xeon E5-2660 v4 @ 2.00 GHz machines.

Trajectory evaluation was performed with TRAVIS[11]. The radial distribution function of the end-to-end distances was calculated between the *para* carbon atoms of the terminating benzene rings of the ladder strand as shown in Supplementary Fig. 5.

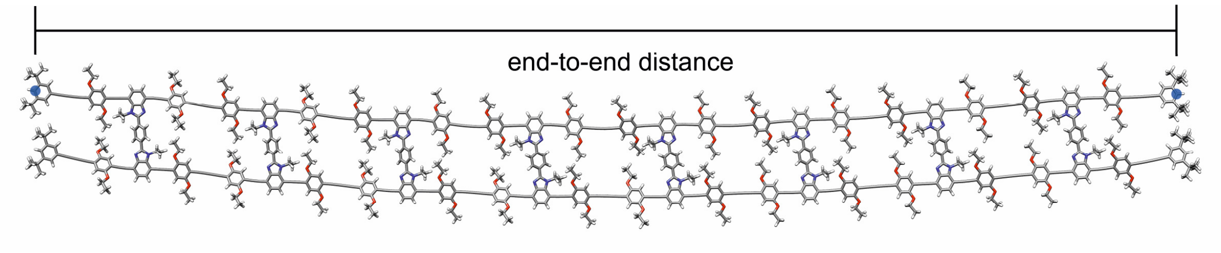

Supplementary Figure 5. Determination of the end-to-end distance. The length is calculated between the *para* carbon atoms of the benzene rings, which terminate one strand of the π-conjugated ladder, shown here for the example of **12_8**.

Supplementary Notes 3: Scanning-tunnelling microscopy

Supplementary Note 3.1: Experimental setup and methods

Scanning tunnelling microscopy (STM) of $\mathbf{12_4}$, $\mathbf{12_8}$ and $\mathbf{12_n}$ was performed under ambient conditions (at room temperature, r.t.) at the solution/solid interface, using 1,2,4-trichlorobenzene (TCB) as the solvent and highly oriented pyrolytic graphite (HOPG) as a substrate. In a typical experiment, 0.2 µL of a 10^{-5} to 10^{-6} M solution of the compound of interest is dropped onto a freshly cleaved HOPG substrate at 80 °C – 100 °C, kept at this temperature for 20 s, and allowed to cool to r.t. before the STM measurements are performed with the tip immersed into the solution. Thermal annealing aimed at overcoming the diffusion barrier of the analytes on the surface, thus promoting self-assembly and increasing supramolecular order. Such a reduction of the lateral degrees of freedom and maximization of the intermolecular interactions in 2D superstructures formed in this way should allow the best image resolution to be achieved in STM, which was one of the main goals here.

Bias voltages between -0.6 V and -1.5 V and tunnelling current set points in the range of 20 pA to 50 pA were applied to image the supramolecular adlayers shown here. The experimental setup consists of an Agilent 5500 scanning-probe microscope that is placed on a Halcyonics actively isolated microscopy workstation. It is acoustically shielded with a home-built isolation box. Pt/Ir (80/20) tips were prepared by cutting the wire with scissors, and were subsequently modified after approach by applying short voltage pulses until the desired resolution was achieved. HOPG was obtained from TipsNano (*via* Anfatec) in ZYB-DS quality. All STM images (unless otherwise noted) were calibrated by subsequent immediate acquisition of an additional image at reduced bias voltage, where the atomic lattice of the HOPG surface is observed, which is used as a calibration grid. Data processing, also for image calibration and noise filtering, was performed using the SPIP 5 (Image Metrology) software package. Molecular and supramolecular modelling was performed using Wavefunction Spartan '18.

Supplementary Notes 3.2: Additional scanning-tunnelling microscopy images

In addition to the STM images shown in Figure 1 of the main text, enlarged sections with additional assignments are shown in Supplementary Figures 6 – 8.

Supplementary Note 3.2.1: Molecular structure and ordering of $\mathbf{12_4}$

At a concentration of $c = 3 \times 10^{-6}$ M, $\mathbf{12_4}$ forms 2D-crystalline domains on HOPG (Supplementary Fig. 6). Submolecular features are clearly resolved, so that the exact shapes and orientations of the four H-shaped subunits of each tetramer can be clearly identified. Dot-shaped contrast features along the rails of the

ladder as well as the rung units are attributed to individual *p*-phenylene units. The observed intramolecular rail-rail distance w = (1.2 ± 0.1) nm as well as the rung-rung distance e = (2.3 ± 0.1) nm both match the expectations from the molecular model. The backbones of **12_4** are aligned in parallel, so that a lamellar packing with a periodicity of b = (2.7 ± 0.1) nm is obtained. In this image, the rails of neighbouring molecules, which appear bright, interact intermolecularly *via* interdigitating hexyloxy side chains that appear as darker image regions but are not submolecularly resolved, as expected for this chain length[12]. However, the side chains are aligned along the HOPG main-axis directions, indicated by the white asterisk in Supplementary Fig. 6[13], and thereby determine the growth direction of the lamella. The end groups of the rigid-rod units of adjacent rows point past each other, and the slight blurring of the corresponding image regions is attributed to residual motional degrees of freedom[14].

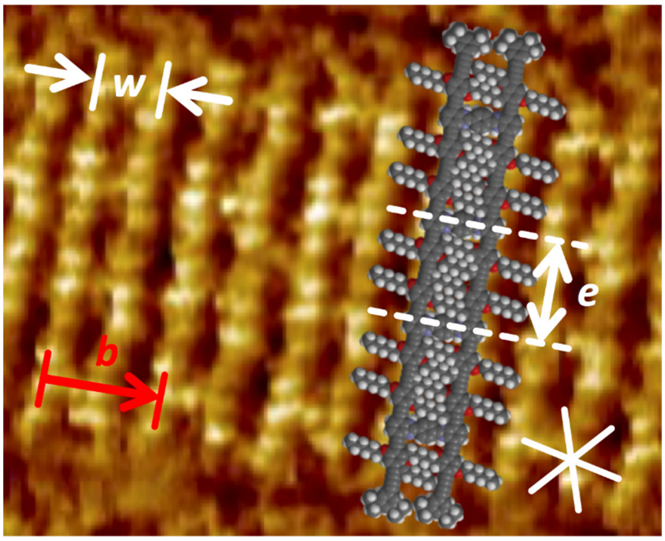

Supplementary Figure 6. Reproduction of the STM image of 12_4 from Figure 1C of the main text with a molecular model superimposed along with additional assignments. Image parameters: 14.9 × 12.0 nm^2, $c = 3 \times 10^{-6}$ M, sample thermally annealed for 20 s at 80 °C, V_S = -0.9 V, I_t = 50 pA. The lines and arrows indicate the rail-rail distance w = (1.2 ± 0.1) nm, the lattice unit vector b = (2.7 ± 0.1) nm, and the rung-rung distance e = (2.3 ± 0.1) nm. The white asterisk indicates the HOPG main axis directions.

Supplementary Note 3.2.2: Molecular structure and ordering of 12_8, and identification of defects

At a concentration of $c = 1 \times 10^{-5}$ M, **12_8** on HOPG forms a quasi-lamellar[15] assembly (Supplementary Fig. 7). Similar to the STM image of **12_4**, the STM image of **12_8** shows submolecularly resolved features, therefore allowing clear insights into the adsorbate pattern. In accordance with the reduced surface mobility related to the increased contact area of the adsorbed species with the substrate, the lamellae of **12_8** do not form adsorbate patterns of two-dimensional crystallinity. Nevertheless, these molecules are

not randomly adsorbed, but the backbones are rather shifted along the normal direction of the lamellae. The intermolecular distances (determined by the interdigitating hexyloxy side-chain periphery) as well as the intramolecular rail-rail distance remain unaltered within the scope of measurement accuracy. However, an example of one defective molecule is seen in Supplementary Fig. 7B. This defect, comprising one interrupted rail and the rotation of the ladder segments around the uninterrupted rail axis is marked by the arrow. Such a single defect would result in a kinked but otherwise rigid polymer chain. In solution, such a kinked chain of $\mathbf{12_n}$ would then more closely resemble the conformation of the open polymer $\mathbf{10_n}$, offering an explanation for the seemingly bimodal appearance of the polarization modulation depth histogram in Supplementary Fig. 4. However, we found no evidence for such defect formation in GPC, MALDI or NMR of any of the compounds, suggesting that such defects are quite rare.

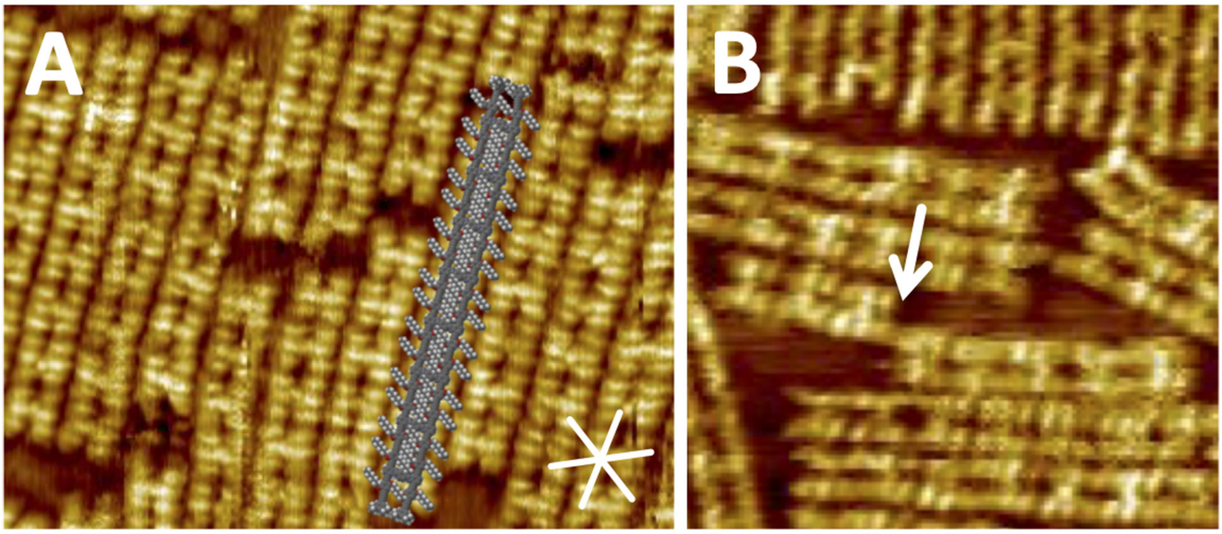

Supplementary Figure 7. STM images of $\mathbf{12_8}$. (A) Reproduction of Figure 1C of the main text with a superimposed molecular model, and (B) an additional STM image with one molecule having an interrupted strand (marked by the arrow). Image parameters: (A) 29.9 × 23.6 nm^2, V_S = -1.5 V, I_t = 37 pA; (B) 23 × 23 nm^2 (internal scanner calibration), V_S = -1.3 V, I_t = 27 pA; both images: $c = 1 \times 10^{-5}$ M, sample thermally annealed for 20 s at 80 °C. The white asterisk in (A) indicates the HOPG main axis directions, and the arrow in (B) indicates an individual defect in one molecule of $\mathbf{12_8}$.

Supplementary Note 3.2.3: Molecular structure and ordering of $\mathbf{12_n}$, and identification of overlapping chains

At a concentration of $c = 2 \times 10^{-6}$ M (assuming the molecular weight of the polymer with a degree of polymerization of $n \sim 40$ as inferred from the GPC data), the assembly of $\mathbf{12_n}$ shown in Supplementary Fig.

8 was observed. As seen in the lower right-hand side of image A, the ladders tend to align in parallel and adopt intermolecular distances matching the intermolecularly interdigitating hexyloxy side chains, similar to those observed for $\mathbf{12_4}$ and $\mathbf{12_8}$. However, the packing order is reduced, which is attributed to both the polydispersity of the sample and a reduced on-surface mobility of these giant molecules in the context of self-assembly. Some extended regions of the ladders, as exemplarily marked by arrows 1 and 2 in Figure S7A, are *not* surrounded by other ladders and are somewhat less sharply resolved in the image when compared to the densely packed ladders $\mathbf{12_n}$ in the lower right-hand corner of Supplementary Fig. 8A and also when compared to $\mathbf{12_4}$ (Supplementary Fig. 6) and $\mathbf{12_8}$ (Supplementary Fig. 7). Moreover, a crossing of two (stacked) rods is clearly identified (arrow 4 in Supplementary Fig. 8A), and a proposed model of the arrangement, with hexyloxy chains omitted, is shown in Supplementary Fig. 8B.

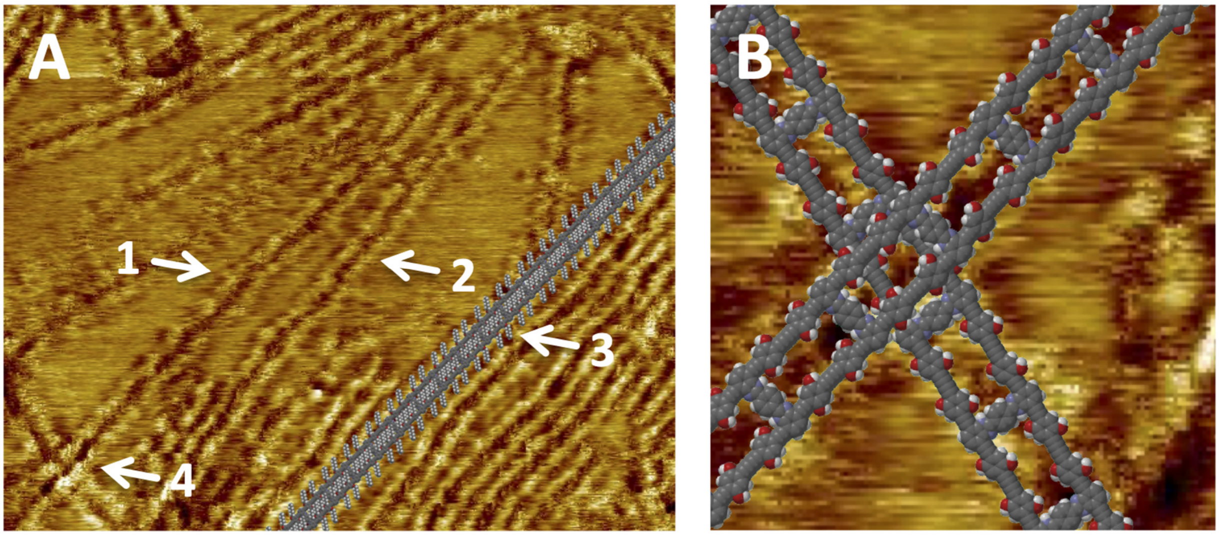

Supplementary Figure 8. STM images of $\mathbf{12_n}$. (A) Enlarged reproduction from Figure 1C of the main text with a molecular model superimposed, and (B) a close-up of A with a superimposed model. Arrows 1 and 2 highlight two individual ladders on HOPG, extended parts of which have no contact to other ladder molecules but retain sufficiently low 2D motional mobility to remain visible in the STM. Arrow 3 marks a molecule with a polymerization degree of > 18 as inferred from the image size, and arrow 4 indicates the crossing of two ladder molecules shown in (B). Image parameters: (A) 50×39 nm^2 (internal scanner calibration), (B) 12.5×12.5 nm^2 cut-out of (A); (A) and (B) $c = 2 \times 10^{-6}$ M (assuming the molecular weight of the oligomer with an average degree of polymerization of $n \sim 40$ as obtained from the GPC data), sample thermally annealed for 20 s at 100 °C prior to STM imaging, V_S = -0.6 V, I_t = 20 pA.

Supplementary Note 3.3: Comment on STM image resolution and visibility of defects

The resolution of the polymer strands, and that of the small molecules, achievable with the STM depends not only on the molecule-surface interactions, but also to a great extent on the intermolecular interactions. Densely packed 2D crystalline monolayers of small molecules, even at the solid/liquid interface at room temperature, usually lead to very high resolution down to the level of individual CH_2 groups (for densely packed alkanes), whereas disordered films, such as those made of polydisperse polymers, are typically visible only with significantly lower resolution. Examples of other STM images of single-stranded arylene-alkynylene polymers have been published by Lei *et al.*[16] and Samorí *et al.*[17], with a rather similar resolution to what we can achieve.

For the case of $\mathbf{12_4}$, $\mathbf{12_8}$, and $\mathbf{12_n}$ this limitation implies the following:

(1) In the 2D crystals or in the densely packed ordered monolayers formed by $\mathbf{12_4}$ and $\mathbf{12_8}$, respectively, the molecules are very well localized and immobile, which translates to a rather high image resolution.

(2) $\mathbf{12_n}$ packs with a significantly lower degree of order, which translates to a higher degree of residual molecular mobility on the surface at room temperature. This thermal motion of the molecules is faster than the timescale of STM measurements, and therefore leads to lower image resolution. In particular, considering the densely packed image region of Supplementary Fig. 8A and Figure 1C (Main Text), right panel, $\mathbf{12_n}$ in the lower right image corner packs densely, and the polymer strands are aligned in a lamellar fashion, so that a high resolution is possible because of the comparatively low molecular mobility. In contrast, the individual isolated strands in the upper central image region are imaged with much lower resolution because of their comparatively higher mobility on the surface.

We have attempted to acquire STM images of the open tetramer $\mathbf{10_4}$ and the octamer $\mathbf{10_8}$, but we have not succeeded in observing any image features that could be related to these species. This failure is only one of the reasons why we expect the examples of extended $\mathbf{12_n}$ molecules shown in the Figure 1C and Supplementary Fig. 8 to be without defects. In addition, we have acquired the STM image shown in Supplementary Fig. 7B, in which one molecule of the octamer $\mathbf{12_8}$ has a single defect, so that different features appear in the STM image. This case does not offer any quantitative insight into the occurrence of defects, but shows that defects can, in principle, be clearly visualized due to the different packing conditions associated with them.

Supplementary Notes 4: Synthesis

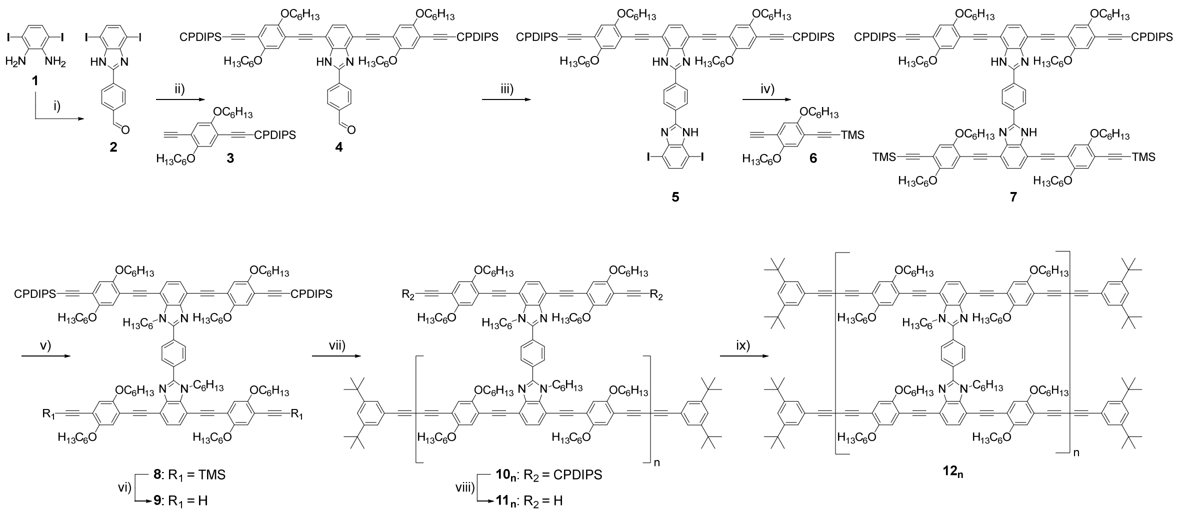

Supplementary Figure 9. **Monomer synthesis and polymerization**. i) Terephthalaldehyde, $ZrCl_4$, $CHCl_3$, r.t., 16 h, 49 %; ii) **3**, $PdCl_2(PPh_3)_2$, CuI, PPh_3, THF, piperidine, r.t., 3 d, 55 %; iii) **1**, $ZrCl_4$, $CHCl_3$, r.t., 15 h; iv) **6**, $PdCl_2(PPh_3)_2$, CuI, PPh_3, THF, piperidine, r.t., 2 d, 57 % for two steps; v) 1-iodohexane, K_2CO_3, acetone, r.t., 2 d, 93 %; vi) K_2CO_3, MeOH, CH_2Cl_2, 40 °C, 5 h, 99 %; vii) oligomerization: 1) CuCl/$CuCl_2$, CH_2Cl_2, pyridine, 25 °C, 3 h; 2) 3,5-di-*tert*-butyl phenylacetylene, 16 h, 4.5 % (**10_4**), 4.3 % (**10_8**); polymerization: 1) CuCl/$CuCl_2$, CH_2Cl_2, pyridine, 25 °C, 16 h; 2) 3,5-di-*tert*-butyl phenylacetylene, 5 h, 2.8 % (**10_n**); viii) Bu_4NF, CH_2Cl_2, THF, 35 °C, 1 to 3 h; ix) 1) CuCl, $CuCl_2$, pyridine, 35 °C, 25 to 45 min, 2) 3,5-di-*tert*-butyl phenylacetylene, 15 to 17 h, 59 % (**12_4**), 54 % (**12_8**), 23 % (**12_n**) for two steps.

Supplementary Note 4.1: Materials and equipment

Commercially available reagents were used without further purification, unless stated otherwise. All oxygen- and moisture-sensitive reactions were carried out using standard Schlenk techniques under argon. Dry solvents (THF, dichloromethane) were obtained from an M. Braun SPS 800 Solvent Drying System under argon atmosphere. Piperidine and pyridine were distilled under argon over CaH_2. Solvents used for workup and purification were either distilled (dichloromethane, cyclohexane, ethyl acetate), or obtained from commercial suppliers of "*p.a.*" quality (methanol, chloroform) or HPLC-grade (THF).

Thin layer chromatography was performed on silica gel-coated aluminium plates (Macherey-Nagel, Alugram SIL G/UV254, 0.25 mm coating with fluorescence indicator). For the detection of the substances, UV light of wavelengths of λ = 254 nm and λ = 366 nm was used. Column chromatography was performed with glass columns with glass frits filled with 40 – 63 µm silica gel from Merck as a stationary phase. The eluents are given in the reaction instructions.

Flash chromatography was performed with an Interchim puriFlash® Minibox pump and Interchim puriFlash® columns, already packed with 30 µm silica gel (PF-30SI-JP/40G) or 15 µm C_{18} reversed phase silica gel (PT-15C18T-F0012). For reversed phase performances a precolumn (F0012) filled with 50 µm C_{18} reversed-phase silica gel (IR-50C18) was used in combination with the already packed column.

^{1}H- and ^{13}C-NMR spectra were recorded on a Bruker Avance I 400 MHz, Bruker Avance I 500 MHz, Bruker Avance III HD 500 MHz Prodigy or Bruker Avance III HD 700 MHz Cryo. Chemical shifts are given in parts per million (ppm) referenced to residual ^{1}H- or ^{13}C-signals in deuterated solvents. All NMR spectra were recorded at room temperature.

EI-MS spectra were measured on a Thermo Finnigan MAT 95 XL. MALDI-MS spectra were recorded on an ultrafleXtreme MALDI-TOF/TOF spectrometer from Bruker Daltronik. DCTB or dithranol were used as a matrix.

Analytical GPC was performed in THF at 35 °C on an Agilent Technologies system at a flow rate of 1 mL/min using an IsoPump G1310A, ALS G1329A autosampler, PSS columns (set of 4 columns 8 mm x 300 mm, polystyrene, porosity of 10^{2}, 10^{3}, 10^{5} and 10^{6} Å, with precolumn, Polymer Standards Service GmbH), and a VWD G1314B and a RID G1362A detector. Calibration was carried out with polystyrene standards from Polymer Standards Service GmbH.

Recycling GPC (recGPC) was performed in THF at 35 °C on a Shimadzu system at a flow rate of 5 mL/min using a LC-20 AD pump, DGU-20 A3 degasser, SIL-20 A HAT autosampler, CTO-20 A oven, FRC-10 A fraction collector, FCV-20 AH2 switching valve, PSS columns (set of 3 columns 20 mm x 300 mm, polystyrene, preparative PSS SDV linear S with precolumn 20 mm x 50 mm, preparative PSS SDV) and a SPD-20A UV-detector (λ_1 = 254 nm and λ_2 = 366 nm).

Supplementary Note 4.2: Synthesis of the starting materials

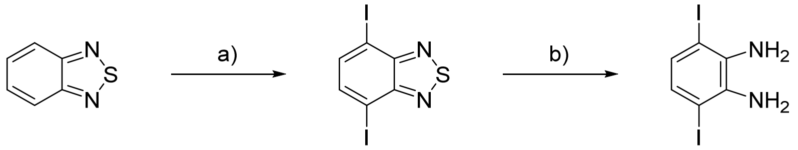

Supplementary Figure 10. Synthesis of 1: a) I_2, Ag_2SO_4, conc. H_2SO_4, 24 h, 70 °C, 54%; b) $NaBH_4$, $CoCl_2 \cdot 6\ H_2O$, THF, MeOH, 3 h, reflux, 35%.

14

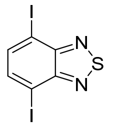

14 was prepared by combining the synthetic procedures of Gampe *et al.*[18] and M. Shimada *et al.*[19].

2,1,3-benzothiadiazole (**13**) (2.79 g, 20.4 mmol), iodine (16.3 g, 64.2 mmol) and silver(I) sulfate (31.7 g, 102 mmol) were suspended in concentrated sulfuric acid (80 mL). The reaction mixture was stirred at 70 °C for 24 h. After cooling to ambient temperature, water was added and the resulting suspension was filtered. The pale-yellow precipitate was suspended in toluene and filtered again. The final filtrate was concentrated under reduced pressure. After adding EtOH, a yellow solid precipitated and was collected by filtration. **14** was obtained in the form of yellow crystals (4.25 g; 10.9 mmol; 54%).

Formula: $C_6H_2I_2N_2S$

Mol weight: 387.97 g mol^{-1}.

^{1}H-NMR (400 MHz, $CDCl_3$, r.t.): δ [ppm] = 7.81 (s, 2H).

^{13}C-NMR (126 MHz, $CHCl_3$, r.t.): δ [ppm] = 154.1, 140.0, 87.9.

MS (EI): *m/z* (%) = 387.9 (100) $[M]^{+\bullet}$, 261.0 (20) $[M\text{-}I]^+$; calculated: 387.80.

1

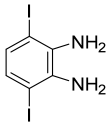

1 was prepared analogously to a modified synthetic procedure of Hündgen *et al*.[14]

Under an Ar atmosphere, **14** (2.00 g, 5.16 mmol) was dissolved in THF (6 mL). Ethanol (17 mL), sodium borohydride (779 mg, 20.6 mmol) and cobalt(II) chloride hexahydrate (6.1 mg, 25.8 µmol) were added. The reaction mixture was stirred for 3 h under reflux. After cooling to ambient temperature, the reaction mixture was evaporated to dryness. Dichloromethane and water were added, the phases were separated and the aqueous layer was extracted with dichloromethane. The combined organic layers were washed with water and brine, dried (Na_2SO_4) and the solvent was evaporated. The crude product was purified *via* flash chromatography (30 µm silica gel; dichloromethane/cyclohexane: 2/1; R_f = 0.40). The product was obtained as a yellow crystalline solid (656 mg; 1.82 mmol; 35 %).

Formula: $C_6H_6I_2N_2$

Mol weight: 359.94 g mol^{-1}.

^{1}H-NMR (400 MHz, DMSO-d_6, r.t.): δ [ppm] = 6.69 (s, 2H), 4.88 (s, 4H, NH_2).

^{13}C-NMR (126 MHz, DMSO-d_6, r.t.): δ [ppm] = 134.7, 128.1, 83.8.

MS (EI): *m/z* (%) = 359.9 (100) $[M]^{+\bullet}$, 232.9 (16) $[M\text{-}I]^+$, 106.0 (11) $[M\text{-}2I]^+$; calculated: 359.86.

2

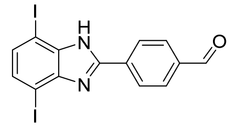

1 (2.23 g, 6.19 mmol) and terephthalaldehyde (1.66 g, 12.4 mmol) were dissolved in chloroform (100 mL) and zirconium(IV) chloride (144 mg, 619 µmol) was added. The reaction mixture was stirred at ambient temperature for 16 h. The pale-yellow suspension was evaporated to dryness and the crude product was recrystallized from acetonitrile. In this case, the double-condensed by-product was the precipitate, therefore it was filtered off and the filtrate was evaporated to dryness. A second recrystallization from acetonitrile gave **2** (1.44 g; 3.05 mmol; 49 %) as a yellow solid.

Formula: $C_{14}H_{8}I_{2}N_{2}O$

Mol weight: 474.04 g mol^{-1}.

^{1}H-NMR (400 MHz, DMSO-*d*$_6$, r.t.): δ [ppm] = 13.21 (bs, 1H, NH)*, 10.11 (s, 1H), 8.57 – 8.53 (m, 2H), 8.11 – 8.08 (m, 2H), 7.44 (s, 2H).

^{13}C-NMR (101 MHz, DMSO-*d*$_6$, r.t.): δ [ppm] = 192.8, 150.6, 137.0, 134.3, 129.8, 128.1.**

MS (EI): *m/z* (%) = 473.9 (100) [M]$^{+\bullet}$; calculated: 473.87.

Annotations:

*The broad signal belongs to the tautomeric NH, the shape and intensity of the signal can differ.

**The number of carbon signals in the spectrum is less than expected, highly probably a result of signal broadening due to the imine-enamine tautomerism.

3

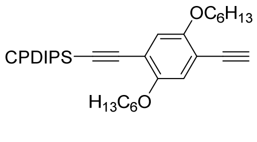

3 was synthesized according to Gaefke *et al.*[20]

4

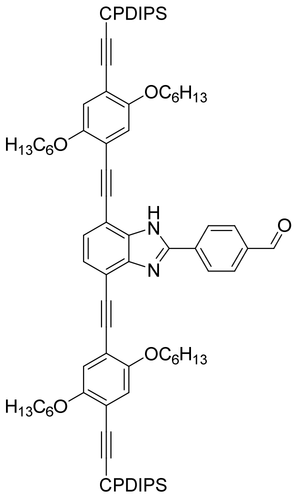

Under an Ar atmosphere, **2** (678 mg, 1.43 mmol), $Pd(PPh_3)_2Cl_2$ (50 mg, 71.5 µmol), CuI (27 mg, 143 µmol), PPh_3 (75 mg, 286 µmol) and **3** (1.50 g, 2.95 mmol) were placed in a Schlenk tube. THF (40 mL) and piperidine (20 mL) were saturated with argon for 30 min and then added to the solids. The reaction mixture was stirred at ambient temperature for 3 d. The reaction was diluted with aq. hydrochloric acid (10%) and dichloromethane. The layers were separated and the aqueous layer was extracted with dichloromethane. The combined organic layers were washed with water and brine, dried (Na_2SO_4) and the solvent was evaporated. The crude product was purified by column chromatography (dichloromethane/ethyl acetate: 40/1, R_f = 0.47) to yield **4** (970 mg; 786 µmol; 55 %) as a yellow oil which solidified upon storing.

Formula: $C_{78}H_{104}N_4O_5Si_2$

Mol weight: 1233.88 g mol^{-1}.

^{1}H-NMR (400 MHz, $CDCl_3$, r.t.): δ [ppm] = 10.06 (s, 1H), 8.40 (d, J = 7.9 Hz, 2H), 7.98 (d, J = 7.9 Hz, 2H), 7.45 (s, 2H), 7.16 (s, 2H), 6.98 (s, 2H), 4.08 (t, J = 6.6 Hz, 4H), 4.01 (t, J = 6.4 Hz, 4H), 2.44 (t, J = 7.0 Hz, 4H), 1.96 – 1.73 (m, 12H), 1.56 – 1.43 (m, 8H), 1.39 – 1.19 (m, 16H), 1.18 – 1.01 (m, 28H), 0.96 – 0.74 (m, 16H).

^{13}C-NMR (126 MHz, $CDCl_3$, r.t.): δ [ppm] = 191.4, 154.7, 153.3, 150.7, 137.5, 130.1, 128.0, 127.3, 119.9, 117.9, 116.5, 114.2, 114.0, 103.9, 95.8, 70.3, 69.4, 31.8, 31.7, 29.5, 29.5, 26.0, 25.8, 22.8, 22.7, 21.5, 20.9, 18.4, 18.2, 14.2, 14.1, 12.0, 9.8.*

MS (MALDI-TOF, DCTB): m/z (%) = 1482.905 (28) $[M+DCTB]^+$, 1232.759 (100) $[M]^+$; calculated: 1232.75.

*Annotation: The number of carbon signals in the spectrum is less than expected, highly probably a result of signal broadening due to the imine-enamine tautomerism.

5

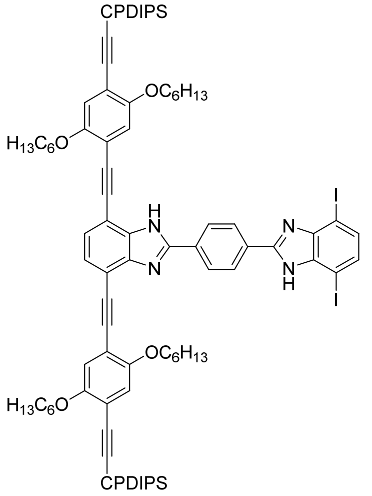

4 (970 mg, 786 µmol) and freshly synthesized **1** (653 mg, 1.81 mmol) were dissolved in chloroform (45 mL). Zirconium(IV) chloride (18 mg, 77.2 µmol) was added, and the resulting reaction mixture was stirred at ambient temperature for 15 h. The solvent was removed under reduced pressure and the crude product was purified *via* column chromatography (dichloromethane/ethyl acetate: 40/1, R_f = 0.45). A yellow film was obtained.

Due to the imine-enamine tautomerism the signals in the proton and carbon NMR spectra are significantly broadened, so that an analysis of the spectra is not possible. Therefore, it is not possible to make any statement on the purity of the compound. For this reason, no yield is given in this step.

Formula: $C_{84}H_{106}I_2N_6O_4Si_2$

Mol weight: 1573.79 g mol^{-1}.

MS (MALDI-TOF, DCTB): *m/z* (%) = 1822.747 (28) $[M+DCTB]^+$, 1572.595 (100) $[M]^+$; calculated: 1572.59.

6

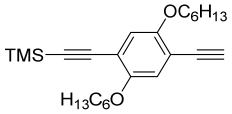

6 was synthesized according to Wang *et al.*[21]

7

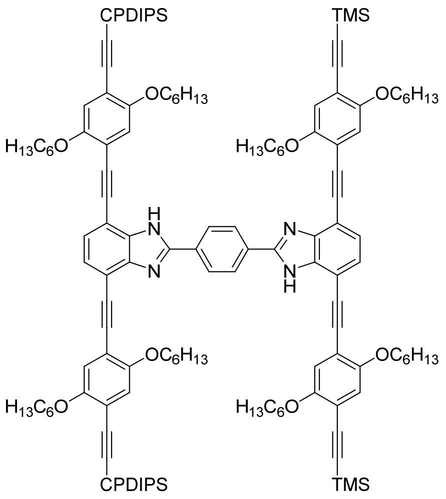

Under an Ar atmosphere, **5** (the crude product of the synthesis, a maximum of 786 µmol is assumed), $Pd(PPh_3)_2Cl_2$ (55 mg, 78.6 µmol), CuI (30 mg, 157 µmol), PPh_3 (82 mg, 314 µmol) and **6** (785 mg, 1.97 mmol) were placed in a Schlenk tube. THF (50 mL) and piperidine (20 mL) were saturated with argon for 30 min and then added to the solids. The reaction mixture was stirred at ambient temperature for 2 d. The reaction was diluted with aq. hydrochloric acid (10%) and dichloromethane. The layers were separated and the aqueous layer was extracted with dichloromethane. The combined organic layers were washed with water and brine, dried (Na_2SO_4) and the solvent was evaporated. The crude product was purified by column chromatography (dichloromethane/ethyl acetate: 40/1, $R_f = 0.52$) and recGPC. Subsequent purification by flash chromatography (15 µm C_{18} reversed phase silica gel; methanol/chloroform: 4/1 with a gradient to pure chloroform) yielded **7** (948 mg; 448 µmol; 57 % for two steps) as a yellow film.

Formula: $C_{134}H_{180}N_6O_8Si_4$

Mol weight: 2115.29 g mol^{-1}.

^{1}H-NMR (500 MHz, CD_2Cl_2, r.t.): δ [ppm] = 11.16 (bs, 2H, NH)*, 8.31 – 8.21 (m, 4H), 7.40 (s, 4H), 7.15 – 7.04 (m, 4H), 7.04 – 6.94 (m, 4H), 4.10 – 3.94 (m, 16H), 2.43 (t, J = 7.0 Hz, 4H), 1.93 – 1.73 (m, 20H), 1.60 – 1.40 (m, 16H), 1.40 – 1.04 (m, 60H), 0.95 – 0.68 (m, 28H), 0.27 (s, 18H).

*The broad signal belongs to the tautomeric NH, the shape and intensity of the signal can differ.

^{13}C-NMR (126 MHz, $CDCl_3$, r.t.): The carbon spectrum only shows signals in the alkyl region, highly probable a result of signal broadening due to the imine-enamine tautomerism.

MS (MALDI-TOF, DCTB): *m/z* (%) = 2613.6 (13) [M+2DCTB]$^+$, 2363.4 (67) [M+DCTB]$^+$, 2113.3 (100) [M]$^+$; calculated: 2113.29.

Analytical GPC (PS-calibrated in THF): $M_p = 2.7 \cdot 10^3$ g mol^{-1}.

8

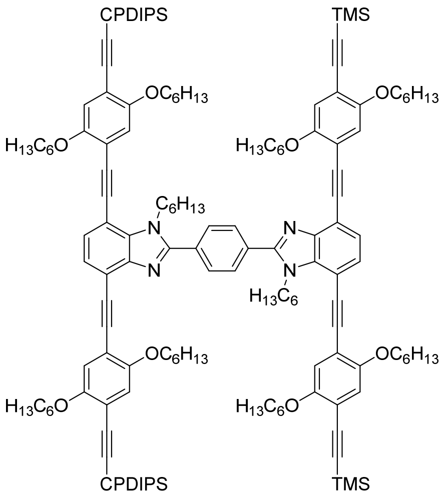

7 (374 mg, 177 µmol) was dissolved in acetone (50 mL). Potassium carbonate (98.0 mg, 707 µmol) and 1-iodohexane (0.52 mL, 3.54 mmol) were added and the reaction mixture was stirred at ambient temperature for 2 d. The solvent was removed under reduced pressure and the crude product was purified by flash chromatography (30 µm silica gel; dichloromethane/cyclohexane: 5/3). **8** (375 mg; 164 µmol; 93 %) was obtained as a yellow film.

Formula: $C_{146}H_{204}N_6O_8Si_4$

Mol weight: 2283.61 g mol^{-1}.

^{1}H-NMR (700 MHz, CD_2Cl_2, r.t.): δ [ppm] = 7.98 (s, 4H), 7.52 – 7.47 (m, 4H), 7.11 (s, 1H), 7.10 (s, 1H), 7.04 (s, 2H), 7.00 (s, 2H), 6.99 (s, 1H), 6.90 (s, 1H), 4.81 (t, *J* = 7.7 Hz, 4H), 4.08 – 3.99 (m, 16H), 2.44 (t, *J* = 7.0 Hz, 2H), 2.44 (t, *J* = 7.0 Hz, 2H), 1.92 – 1.76 (m, 24H), 1.60 – 1.45 (m, 16H), 1.40 – 1.26 (m, 32H), 1.18 – 1.06 (m, 40H), 0.94 – 0.82 (m, 28H), 0.74 – 0.69 (m, 6H), 0.27 (s, 9H), 0.27 (s, 9H).

^{13}C-NMR (176 MHz, CD_2Cl_2, r.t.): δ [ppm] = 155.1, 155.1, 154.9, 154.8, 154.7, 154.6, 154.3, 154.2, 154.2, 154.1, 144.9, 144.9, 134.8, 134.8, 132.7, 132.6, 130.6, 128.6, 126.8, 126.8, 120.3, 120.3, 118.1, 117.8, 117.5, 117.5, 117.4, 117.2, 116.9, 116.7, 115.9, 115.0, 114.9, 114.8, 114.5, 114.5, 114.2, 114.0, 108.2, 104.4, 104.2, 101.8, 101.6, 101.0, 100.7, 96.3, 96.0, 92.8, 92.7, 92.3, 92.2, 92.2, 92.2, 91.7, 91.6, 70.4, 70.3,

70.2, 70.1, 70.0, 70.0, 69.9, 69.9, 46.3, 46.3, 32.3, 32.2, 32.2, 32.2, 32.2, 32.2, 31.9, 31.9, 30.0, 29.9, 29.9, 29.9, 29.9, 29.9, 26.5, 26.5, 26.4, 26.4, 26.3, 26.3, 26.3, 26.3, 26.3, 23.3, 23.2, 23.2, 23.2, 23.2, 23.0, 23.0, 21.9, 21.9, 21.3, 21.3, 18.6, 18.6, 18.4, 18.3, 14.5, 14.4, 14.4, 14.4, 14.2, 12.4, 12.4, 10.2, 0.4, 0.2, 0.2.

MS (MALDI-TOF, DCTB): *m/z* (%) = 2783.8 (74) $[M+2DCTB]^+$, 2533.6 (100) $[M+DCTB]^+$, 2283.5 (30) $[M]^+$; calculated: 2281.48.

Analytical GPC (PS-calibrated in THF): $M_p = 2.8 \cdot 10^3$ g mol^{-1}.

9

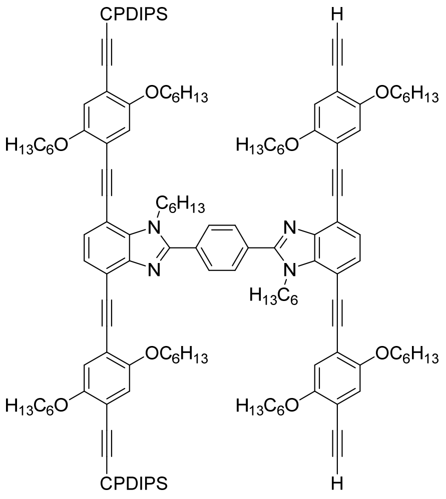

8 (128 mg, 56.0 µmol) was dissolved in dichloromethane (30 mL). Methanol (30 mL) and potassium carbonate (80 mg, 579 µmol) were added and the reaction mixture was stirred at 40 °C for 5 h. Aq. HCl (10%) and dichloromethane were added. The layers were separated and the aqueous layer was extracted with dichloromethane. The combined organic layers were washed with water and brine, dried (Na_2SO_4) and the solvent was removed. The crude product was purified by flash chromatography (30 µm silica gel; dichloromethane/cyclohexane: 10/1) and **8** (118 mg; 55.2 µmol; 99 %) was obtained as a yellow film.

Formula: $C_{140}H_{188}N_6O_8Si_2$

Mol weight: 2139.25 g mol^{-1}.

^{1}H-NMR (500 MHz, CD_2Cl_2, r.t.): δ [ppm] = 7.98 (s, 4H), 7.52 – 7.47 (m, 4H), 7.12, (s, 1H), 7.10 (s, 1H), 7.06 (s, 1H), 7.03 (s, 1H), 7.03 (s, 1H), 7.02 (s, 1H), 6.99 (s, 1H), 6.99 (s, 1H), 4.80 (t, *J* = 7.5 Hz, 4H), 4.08 – 3.97 (m, 16H), 3.42 (s, 1H), 3.40 (s, 1H), 2.44 (t, *J* = 7.0 Hz, 2H), 2.43 (t, *J* = 7.0 Hz, 2H), 1.92 – 1.74 (m, 24H), 1.59 – 1.44 (m, 16H), 1.41 – 1.27 (m, 32H), 1.17 – 1.06 (m, 40H), 0.95 – 0.82 (m, 28H), 0.74 – 0.68 (m, 6H).

^{13}C-NMR (126 MHz, CD_2Cl_2, r.t.): δ [ppm] = 155.1, 155.1, 154.9, 154.8, 154.8, 154.7, 154.2, 154.2, 154.1, 154.1, 144.8, 144.8, 134.8, 134.8, 132.6, 132.6, 130.6, 128.6, 126.8, 120.3, 120.3, 118.3, 118.0, 117.7, 117.4, 117.3, 117.2, 116.8, 116.7, 115.9, 115.1, 114.9, 114.5, 114.4, 114.1, 114.1, 113.6, 113.2, 108.2, 108.1, 104.3, 104.2, 96.3, 96.0, 92.7, 92.7, 92.3, 92.2, 92.1, 92.0, 91.6, 91.6, 83.1, 82.8, 80.6, 80.4, 70.4, 70.3, 70.3, 70.2, 70.0, 70.0, 69.9, 69.9, 46.2, 32.3, 32.2, 32.2, 32.2, 32.1, 32.1, 32.1, 31.9, 30.0, 29.9, 29.9, 29.8, 29.8, 29.7, 26.5, 26.5, 26.4, 26.3, 26.3, 26.3, 26.3, 26.2, 26.1, 23.2, 23.2, 23.2, 23.2, 23.2, 23.2, 23.0, 21.9, 21.3, 21.2, 18.6, 18.3, 14.4, 14.4, 14.4, 14.4, 14.2, 14.2, 12.4, 12.4, 10.2.

MS (MALDI-TOF, DCTB): m/z (%) = 2636.7 (24) $[M+2DCTB]^+$, 2386.6 (100) $[M+DCTB]^+$, 2137.4 (68) $[M]^+$; calculated: 2137.40.

Analytical GPC (PS-calibrated in THF): $M_p = 2.7\cdot10^3$ g mol^{-1}.

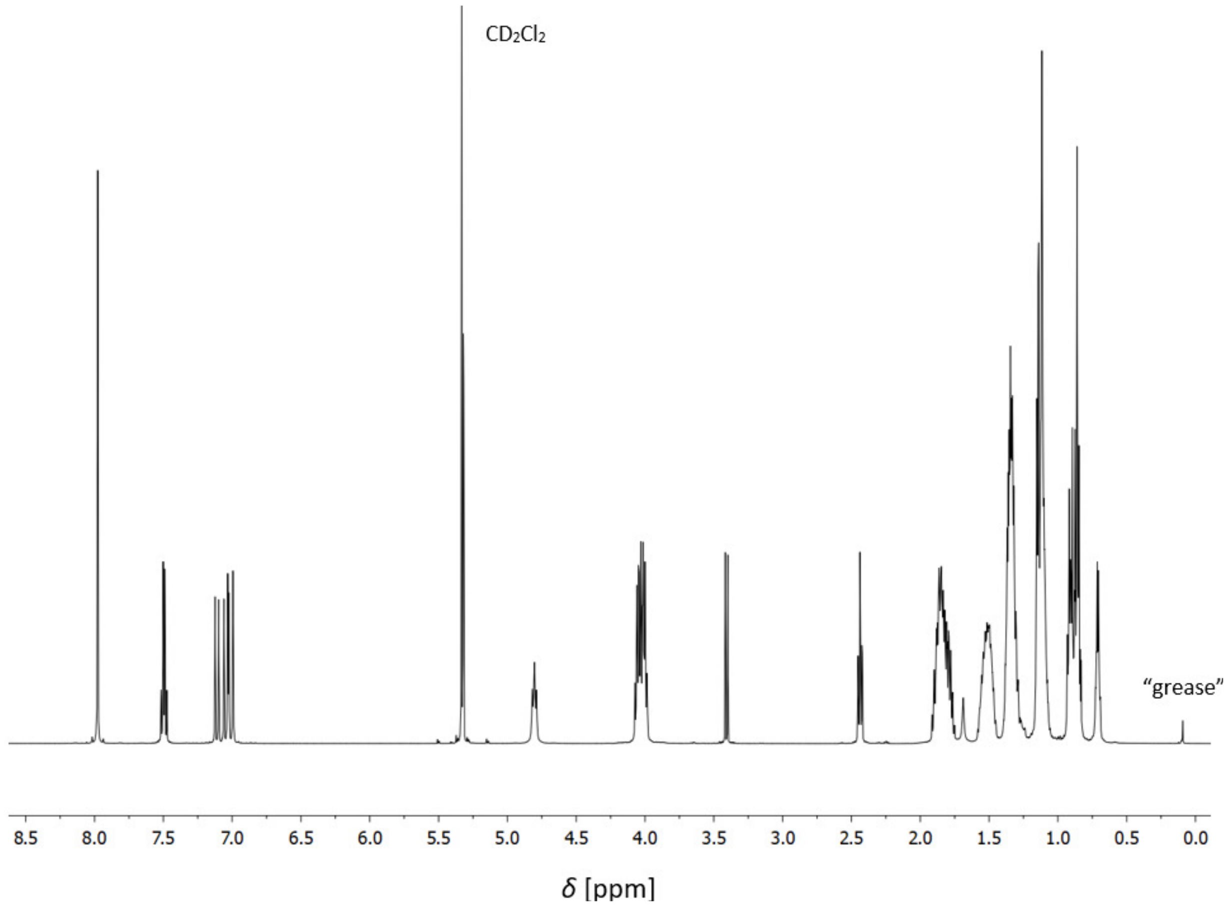

Supplementary Figure 11. **^{1}H-NMR spectrum of 9** in CD_2Cl_2.

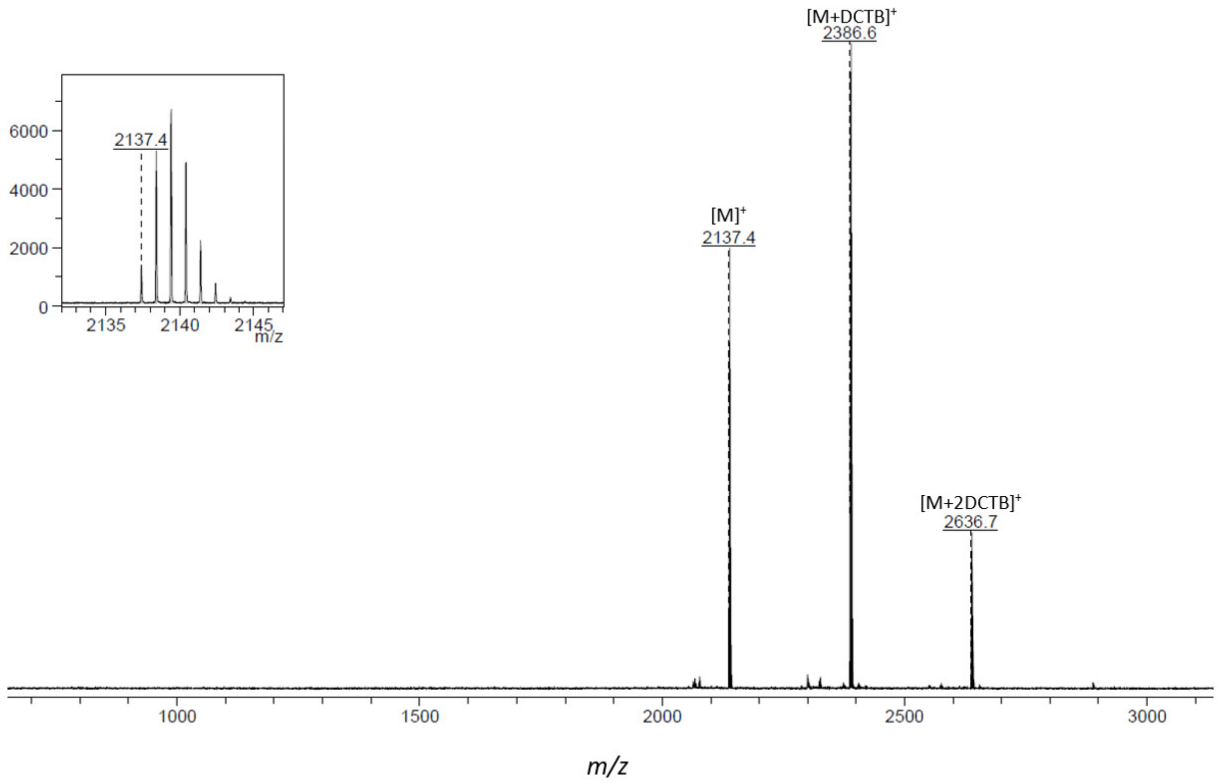

Supplementary Figure 12. MALDI-MS spectrum of 9 (matrix: DCTB).

3,5-Di-*tert*-butyl phenylacetylene

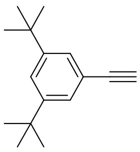

3,5-Di-*tert*-butyl phenylacetylene was synthesized according to Zhang *et al*.[22]

Supplementary Note 4.3: Oligomerization

10_n

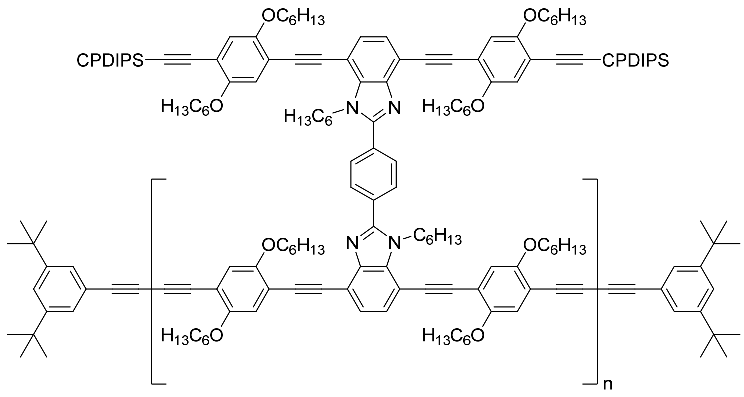

9 (118 mg, 55.2 µmol) was dissolved in dichloromethane (20 mL). Pyridine (20 mL), CuCl (77.2 mg, 780 µmol) and $CuCl_2$ (10.1 mg, 75.4 µmol) were added, and the suspension was stirred for 3 h at 25 °C. 3,5-Di-*tert*-butyl phenylacetylene (240 mg, 1.12 mmol) was added, and the reaction mixture was stirred for an additional 16 h at 25 °C. The mixture was diluted with aq. EDTA-solution (0.1 M) and dichloromethane. The layers were separated and the aqueous layer was extracted with dichloromethane. The combined organic layers were washed with aq. EDTA-solution (0.1 M), aq. HCl (10%), water and brine, dried (Na_2SO_4) and the solvent was evaporated. The crude product was diluted with dichloromethane, filtrated through a pad of silica gel and rinsed with dichloromethane. The solvent was removed under reduced pressure. The different oligomers were first separated coarsely by recGPC, and the tetramer (n = 4) and octamer (n = 8) were then subsequently purified individually by recGPC. Each fraction was dissolved in dichloromethane and precipitated with methanol. After filtration, the oligomers were obtained as a yellow film.

Formula: $C_{140}H_{186}N_6O_8Si_2$ (repeating unit), $C_{32}H_{42}$ (endcap units)

Mol weight: 2137.23 g mol^{-1} (repeating unit), 426.69 g mol^{-1} (endcap units).

^{1}H-NMR (400 MHz, CD_2Cl_2, r.t.): δ [ppm] = 7.99 (s, 4H), 7.56 – 7.46 (m, 4.41H), 7.42 – 7.40 (m, 0.83H), 7.16 – 7.13 (m, 1H), 7.12 – 6.97 (m, 7H), 4.86 – 4.76 (m, 4H), 4.11 – 3.97 (m, 16H), 2.46 – 2.41 (m, 4H), 1.94 – 1.73 (m, 24H), 1.61 – 1.45 (m, 16H), 1.44 – 1.24 (m, 38.64H), 1.23 – 1.02 (m, 40H), 1.00 – 0.79 (m, 28H), 0.79 – 0.67 (m, 6H).

Analytical GPC (PS-calibrated in THF): $M_n = 15.0 \cdot 10^3$ g mol^{-1}, $M_w = 30.1 \cdot 10^3$ g mol^{-1}, $M_p = 26.9 \cdot 10^3$ g mol^{-1}, $PD = 2.0$.

Progress of the oligomerization at different times

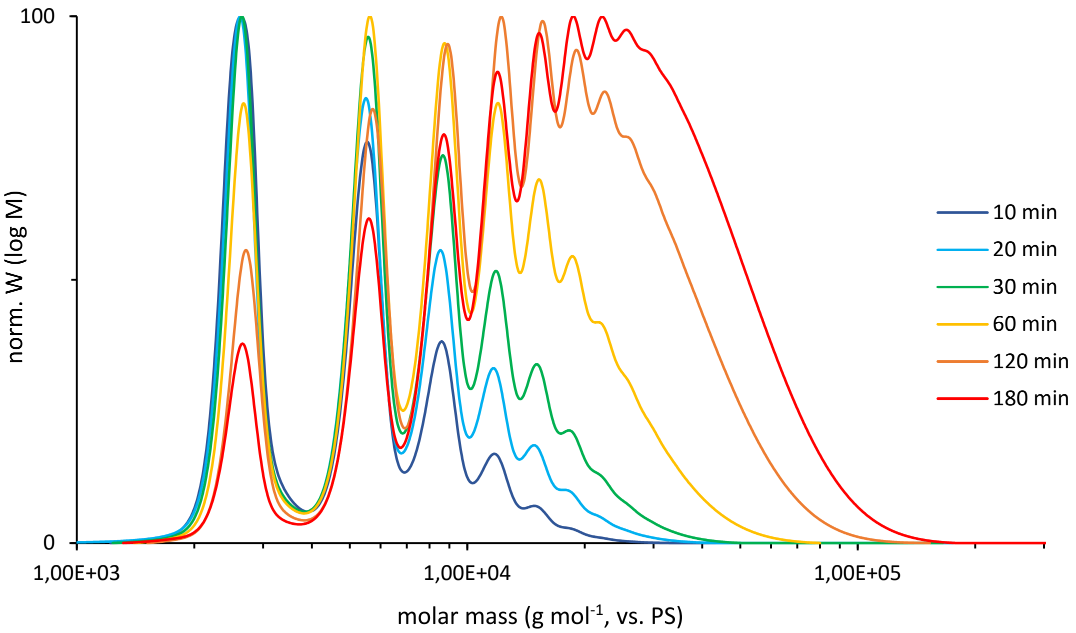

Supplementary Figure 13. Oligomerization of 9:

10 min: M_n = 3.93·10^3 g mol^{-1}, M_w = 5.39·10^3 g mol^{-1}, M_p = 2.63·10^3 g mol^{-1}, *PD* = 1.37;

20 min: M_n = 4.52·10^3 g mol^{-1}, M_w = 6.74·10^3 g mol^{-1}, M_p = 2.62·10^3 g mol^{-1}, *PD* = 1.49;

30 min: M_n = 5.23·10^3 g mol^{-1}, M_w = 8.19·10^3 g mol^{-1}, M_p = 2.65·10^3 g mol^{-1}, *PD* = 1.56;

60 min: M_n = 6.93·10^3 g mol^{-1}, M_w = 1.19·10^3 g mol^{-1}, M_p = 5.62·10^3 g mol^{-1}, *PD* = 1.71;

120 min: M_n = 10.1·10^3 g mol^{-1}, M_w = 18.8·10^3 g mol^{-1}, M_p = 12.2·10^3 g mol^{-1}, *PD* = 1.86;

180 min: M_n = 12.6·10^3 g mol^{-1}, M_w = 24.7·10^3 g mol^{-1}, M_p = 1.87·10^3 g mol^{-1}, *PD* = 1.96.

GPC of the oligomerization after 3 h.

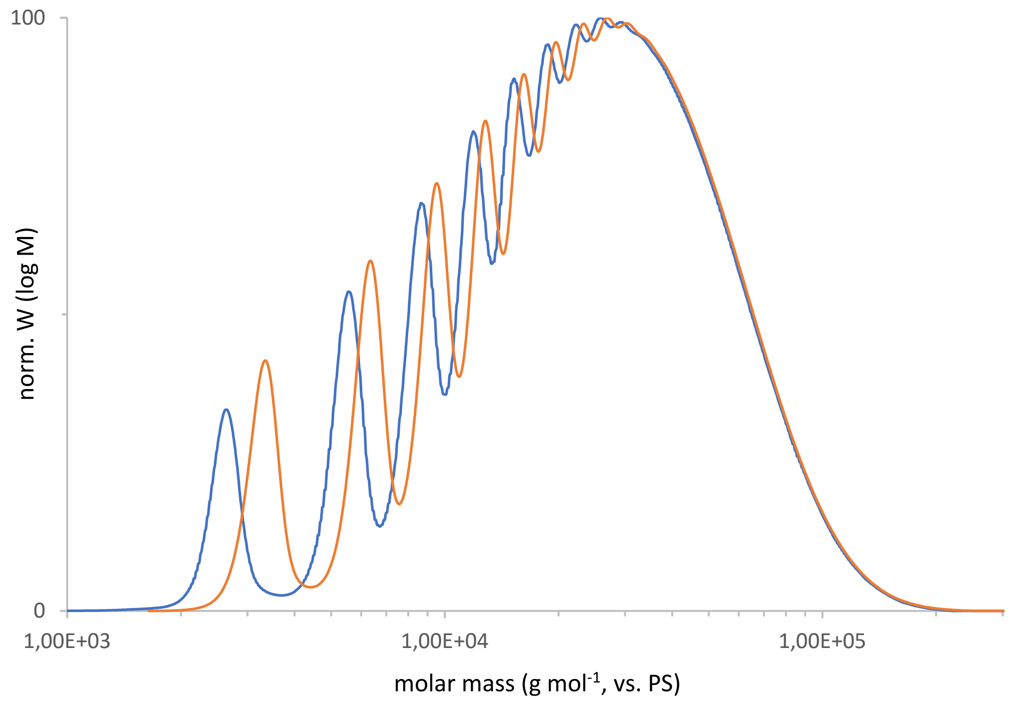

Supplementary Figure 14. Oligomerization of 9 after 3 h. M_w = 29.5·10^3 g mol^{-1}, M_n = 14.2·10^3 g mol^{-1}, M_p = 25.7·10^3 g mol^{-1}, *PD* = 2.1 (blue line), and after endcapping with 3,5-di-*tert*-butyl phenylacetylene: M_n = 15.0·10^3 g mol^{-1}, M_w = 30.1·10^3 g mol^{-1}, M_p = 26.9·10^3 g mol^{-1}, *PD* = 2.0 (orange line).

The degree of polymerization was determined by GPC after 3 h, before 3,5-di-*tert*-butyl phenylacetylene was added in a large excess to terminate the reaction.

$$P_N(GPC) = \frac{M_n}{M_{monomer} - M_{2H}} = \frac{14.2 \cdot 10^3\ g\ mol^{-1}}{2139.2480\ g\ mol^{-1} - 2.0159\ g\ mol^{-1}} = 6.64$$

However, rigid-rod polymers are generally overestimated in their molecular weight when the GPC is calibrated by poly(styrene). The degree of overestimation depends on the polymer backbone, on the length of the specific oligomer, and on the size of the side groups; see, e.g., Hinderer *et al.*[23]

NMR

For the endcapped polymer **10_n** the degree of polymerization can be calculated from the ratio of the signals of the internal phenylene ring of the monomer (red) to the end-group signals (blue).

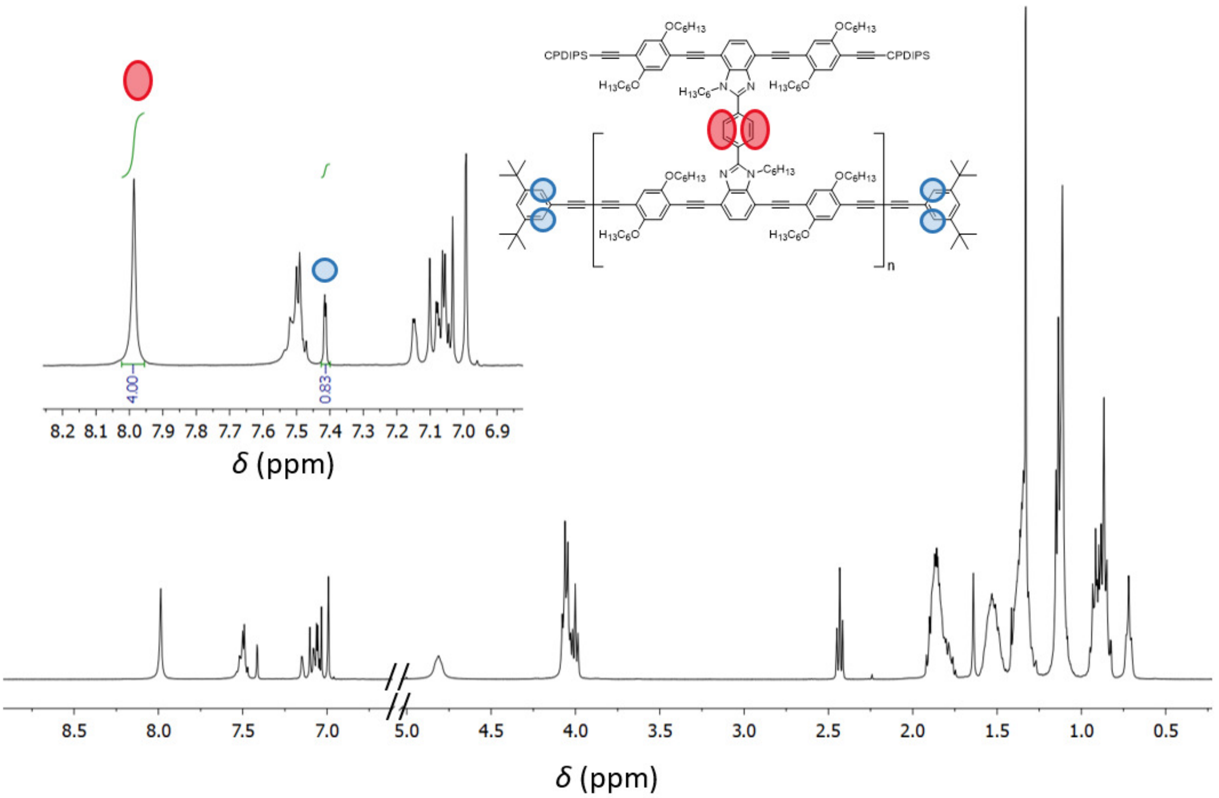

Supplementary Figure 15. ^{1}H-NMR spectrum of 10_n.

$$P_N(NMR) = \frac{Integral\ (8.02-7.95\ ppm)}{Integral\ (7.43-7.41\ ppm)} = \frac{4.00}{0.83} = 4.81$$

GPC separation and GPC overestimation factors

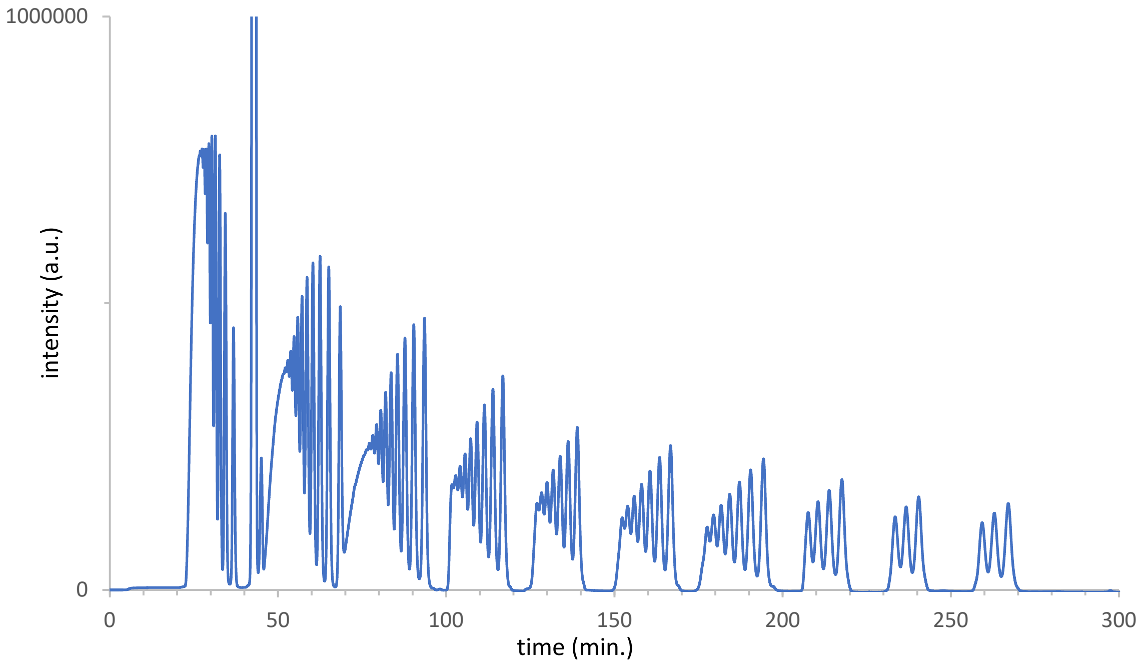

Supplementary Figure 16. **Recycling GPC separation of 10_n.** Note that oligomers are separated in each cycle and also the polymeric part of the sample has been removed during the separation process.

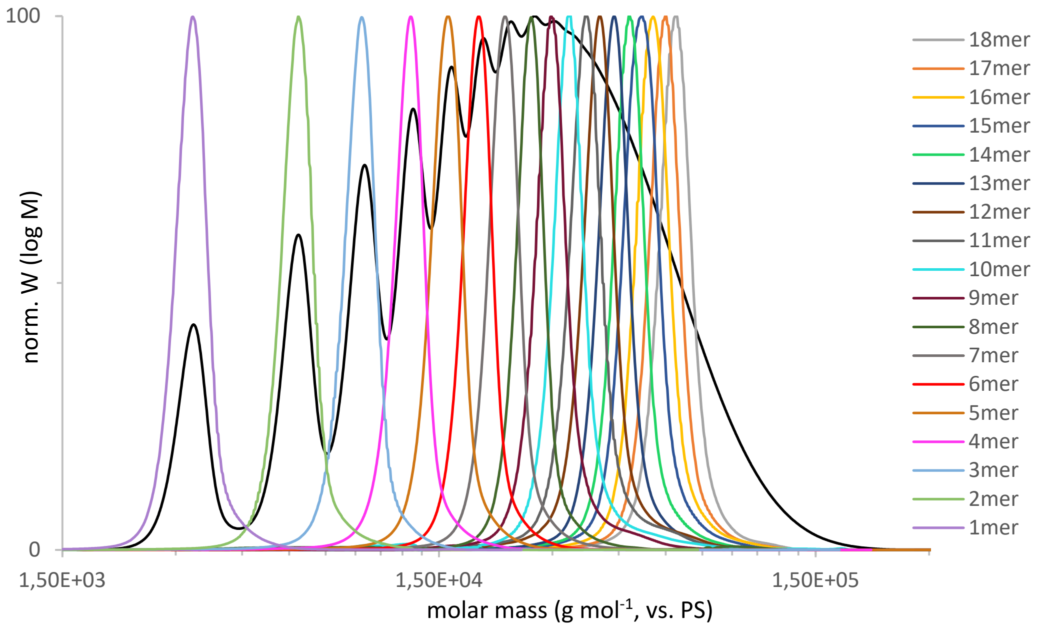

Supplementary Figure 17. **Normalized MWD overlap of the separated oligomers 10_n (n = 1 to 18) and the crude polymerization product (black).**

Overestimation factors

Supplementary Table 1. GPC peaks (M_p) of the separated oligomers of 10_n and overestimation factors f_i.

n	M_{abs} / g mol^{-1}	M_p	f_i
1	2563.92	3327	1.30
2	4701.152	6327	1.35
3	6838.384	9368	1.37
4	8975.616	12620	1.41
5	11112.848	15800	1.42
6	13250.08	19180	1.45
7	15387.312	22470	1.46
8	17524.544	26420	1.51
9	19661.776	29800	1.52
10	21799.008	32890	1.51
11	23936.24	37100	1.55
12	26073.472	39990	1.53
13	28210.704	43650	1.55
14	30347.936	48160	1.59
15	32485.168	52030	1.60
16	34622.4	55760	1.61
17	36759.632	60160	1.64
18	38896.864	64290	1.65

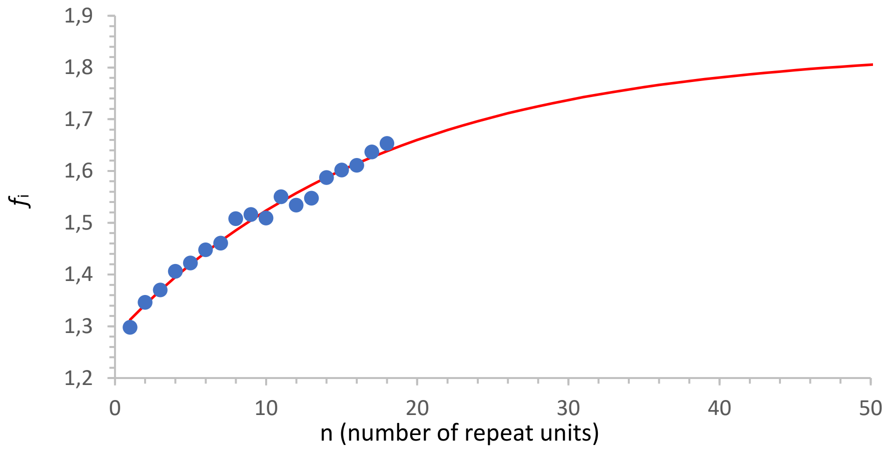

Supplementary Figure 18. Overestimation factors f_i. The fit and extrapolation follows an exponential function of the form $f_i = f_\infty - a\, e^{-n/b}$ with parameters a = 0.555, b = 17.512 and f_∞ = 1.837.

The overestimation factor of 1.4 for the tetramer **10_4** explains quantitatively the higher average degree of oligomerization determined by GPC (P_n = 6.6) compared to the end-group analysis (P_n = 4.8).

Tetramer **10_4** (5.4 mg, 602 nmol, 4.5 %):

Formula: $C_{592}H_{786}N_{24}O_{32}Si_8$

Mol weight: 8975.62 g mol^{-1}.

^{1}H-NMR (700 MHz, CD_2Cl_2, r.t.): δ [ppm] = 7.98 (s, 16H), 7.53 – 7.46 (m, 18H), 7.41 – 7.40 (m, 4H), 7.15 – 7.12 (m, 4H), 7.10 – 6.97 (m, 28H), 4.83 – 4.77 (m, 16H), 4.08 – 3.96 (m, 64H), 2.44 – 2.41 (m, 16H), 1.91 – 1.73 (m, 96H), 1.59 – 1.43 (m, 64H), 1.42 – 1.22 (m, 164H), 1.17 – 1.01 (m, 160H), 0.96 – 0.78 (m, 112H), 0.73 – 0.69 (m, 24H).

MS (MALDI-TOF, DCTB): *m/z* (%) = 11227.2 (3) $[M+9DCTB]^+$, 10977.0 (9) $[M+8DCTB]^+$, 10726.9 (19) $[M+7DCTB]^+$, 10476.8 (46) $[M+6DCTB]^+$, 10226.6 (78) $[M+5DCTB]^+$, 9976.5 (100) $[M+4DCTB]^+$, 9725.3 (90) $[M+3DCTB]^+$, 9476.1 (52) $[M+2DCTB]^+$, 9225.0 (14) $[M+DCTB]^+$, 8974.8 (3) $[M]^+$; calculated: 8967.87.

Analytical GPC (PS-calibrated in THF): M_p = 12.6·10^3 g mol^{-1}.

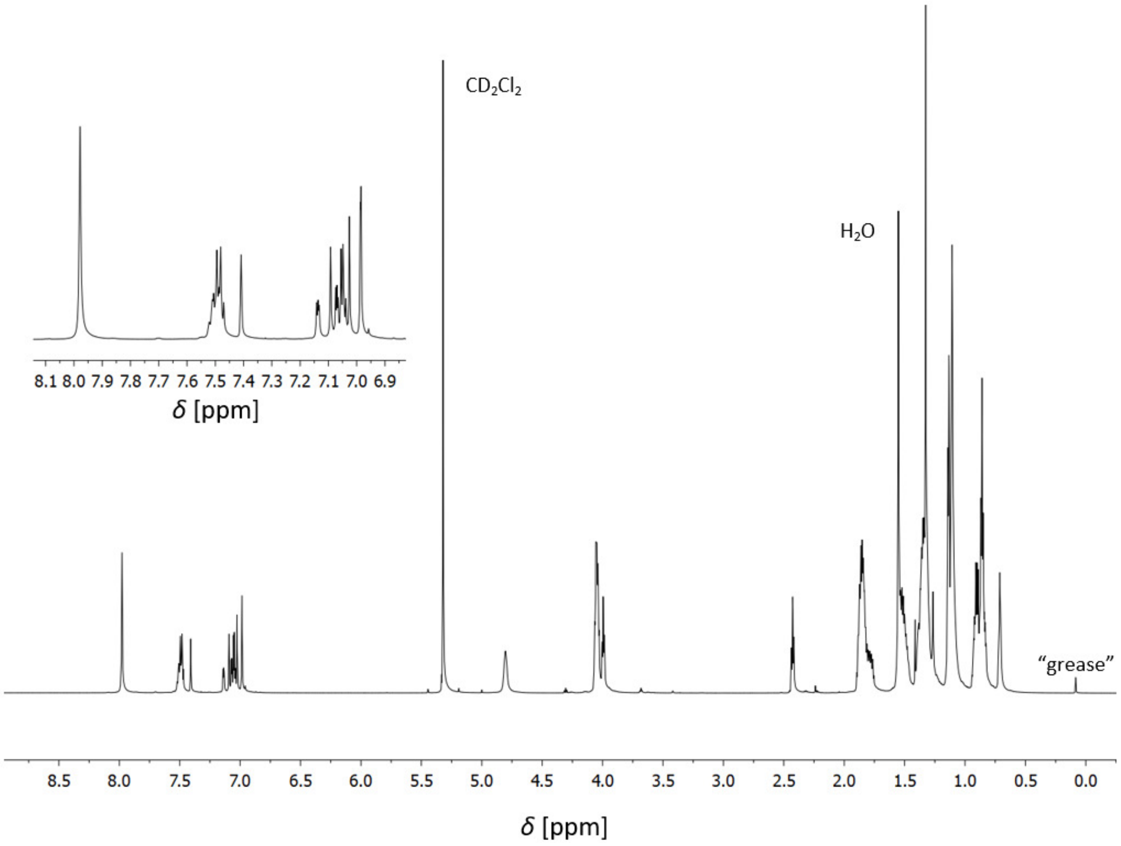

Supplementary Figure 19. ^{1}H-NMR spectrum of 10_4 in CD_2Cl_2.

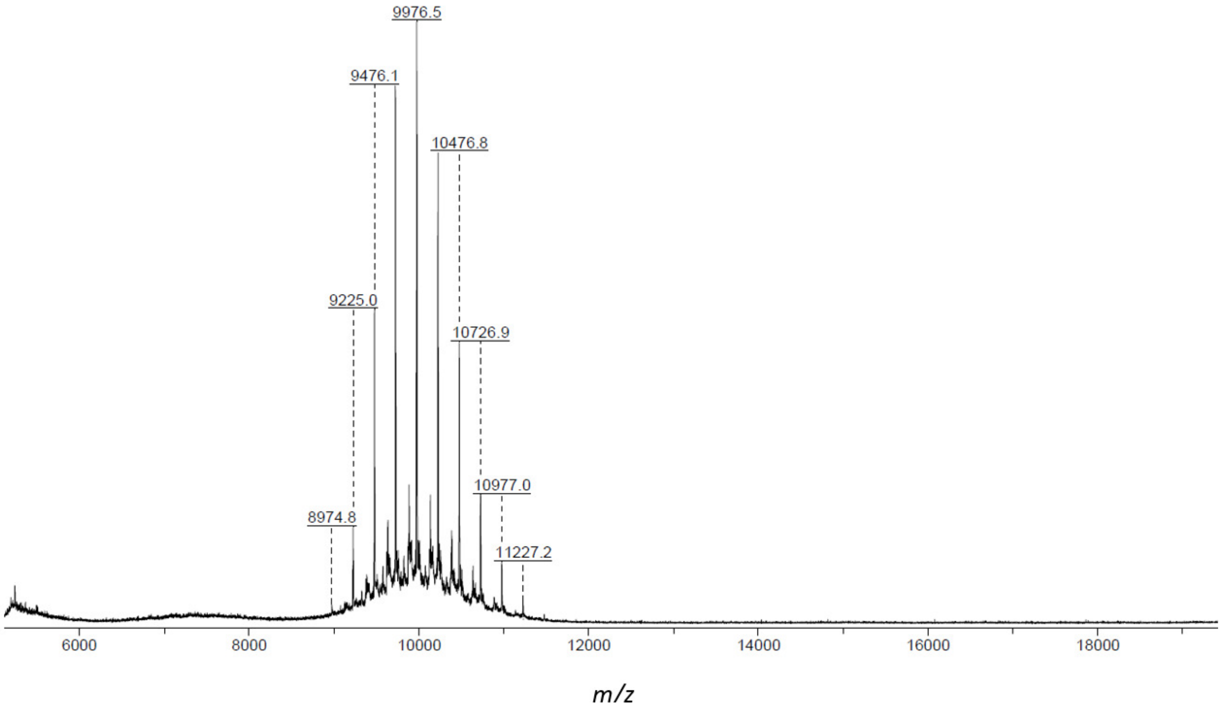

Supplementary Figure 20. MALDI-MS spectrum of 10_4. Matrix: DCTB.

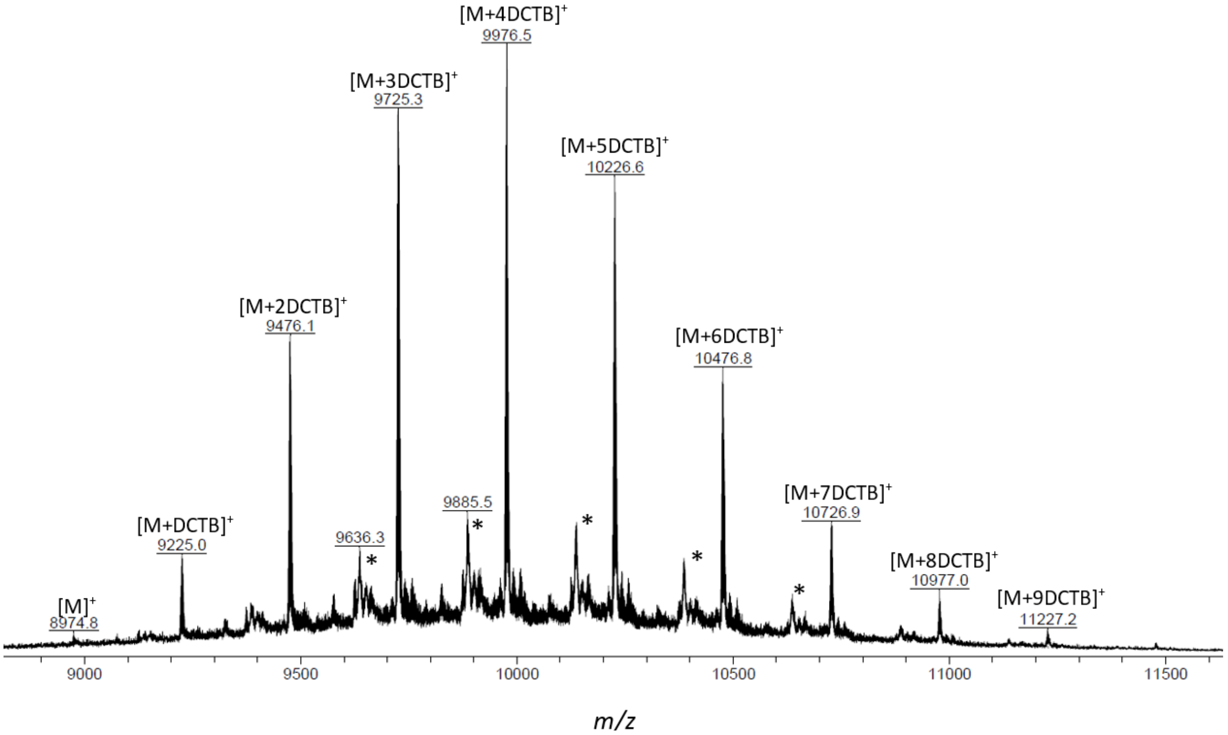

Supplementary Figure 21. Detail of MALDI-MS spectrum of 10_4. Matrix: DCTB; signals labelled with * are due to alkyl fragmentation -C_6H_{13}.

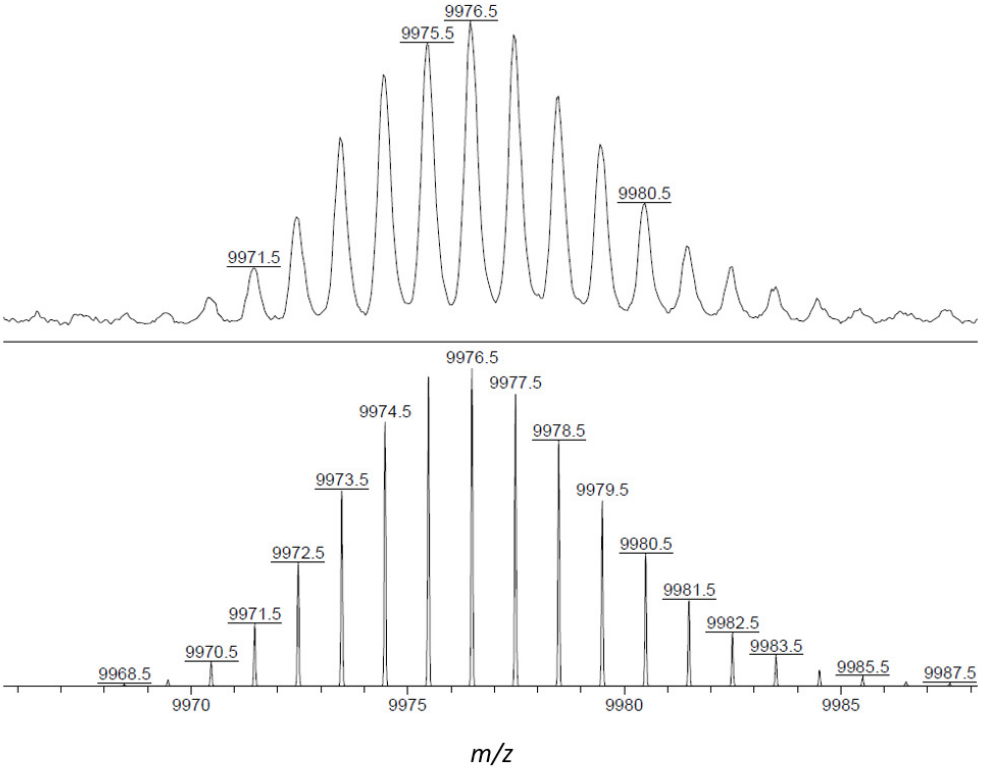

Supplementary Figure 22. Comparison of the observed (top) and calculated (bottom) isotopic signatures of the [M+4DCTB]$^+$ peak of the MALDI-MS spectrum of $\mathbf{10_4}$.

Octamer $\mathbf{10_8}$ (5.1 mg, 291 nmol, 4.3 %):

Formula: $C_{1152}H_{1530}N_{48}O_{64}Si_{16}$

Mol weight: 17524.54 g mol^{-1}.

MS (MALDI-TOF, DCTB): *m/z* (%) = 19777.8 (21) [M+9DCTB]$^+$, 19527.9 (35) [M+8DCTB]$^+$, 19276.8 (54) [M+7DCTB]$^+$, 19027.0 (85) [M+6DCTB]$^+$, 18776.7 (100) [M+5DCTB]$^+$, 18526.8 (99) [M+4DCTB]$^+$, 18275.9 (74) [M+3DCTB]$^+$, 18025.9 (34) [M+2DCTB]$^+$, 17775.3 (10) [M+DCTB]$^+$; calculated: 17509.43.

Analytical GPC (PS-calibrated in THF): $M_p = 26.4 \cdot 10^3$ g mol^{-1}.

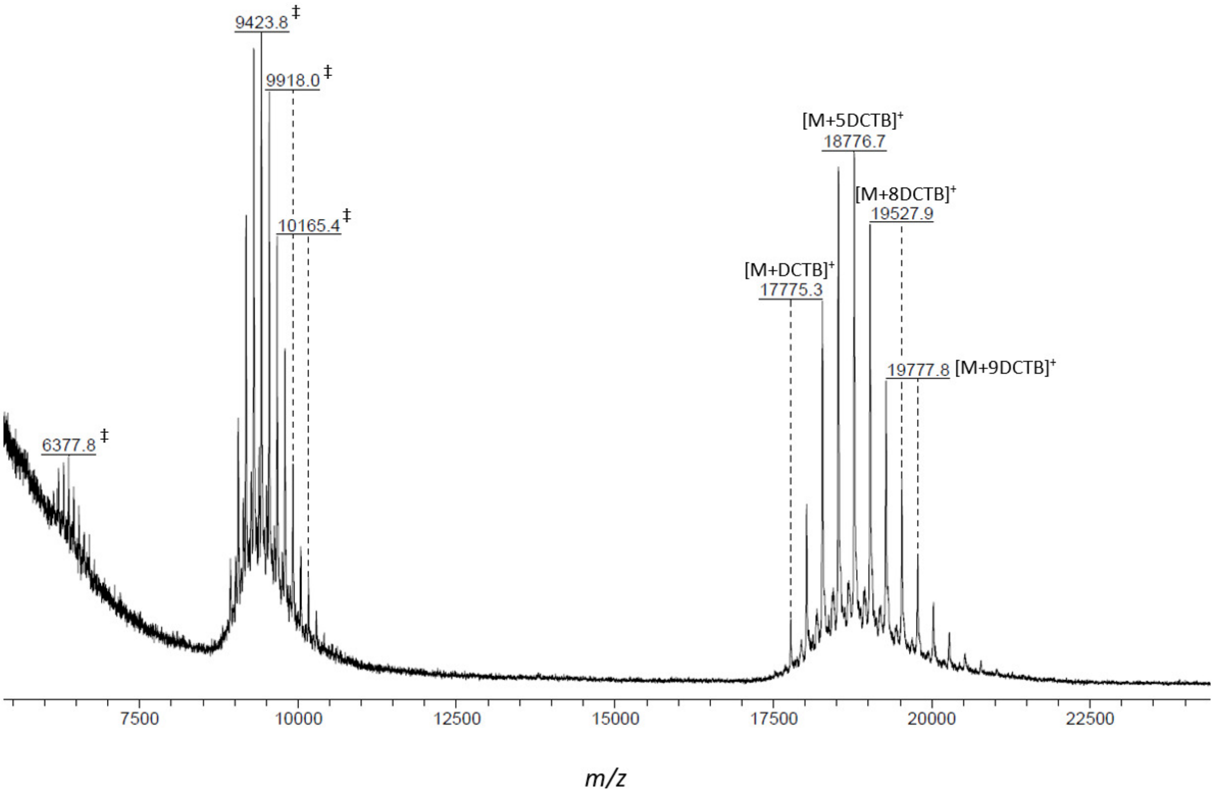

Supplementary Figure 23. MALDI-MS spectrum of 10_8. Matrix: DCTB; signals labelled with ‡ are due to multiply-charged adducts.

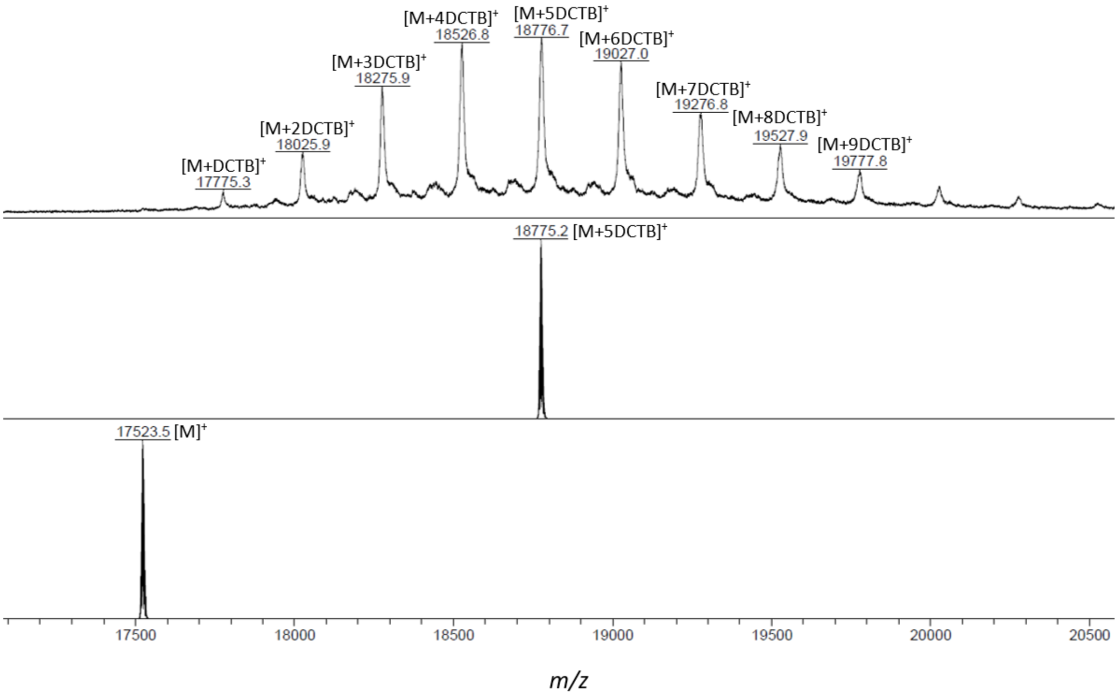

Supplementary Figure 24. Comparison of the detailed MALDI-MS spectrum of 10_8. Top: full spectrum. Middle: calculated $[M+5DCTB]^+$ peak. Bottom: calculated $[M]^+$ peak.

11_4

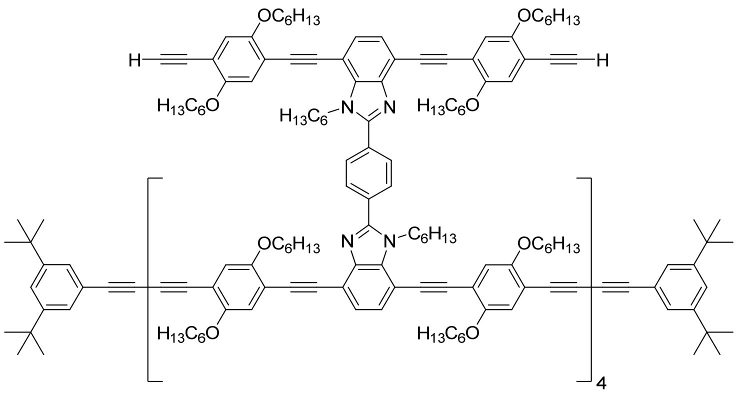

10_4 (4.8 mg, 535 nmol) was dissolved in dichloromethane (6 mL) and tetra-*n*-butylammonium fluoride (1 mL, 1 M in THF) was added. The reaction mixture was stirred at 35 °C for 3 h. Dichloromethane and water were added, and the layers were separated. The aqueous layer was extracted with dichloromethane, the combined organic layers were washed with water, dried (Na_2SO_4) and the solvent was evaporated. The crude product was purified by recGPC. The product was obtained as a yellow film.

Due to the small amount of substance, the product was not further purified as usual by precipitation following recGPC. For this reason, no exact weight was determined and no yield is given in this step.

Formula: $C_{512}H_{634}N_{16}O_{32}$

Mol weight: 7524.78 g mol^{-1}.

^{1}H-NMR (400 MHz, CD_2Cl_2, r.t.): δ [ppm] = 7.98 (s, 16H), 7.54 – 7.45 (m, 18H), 7.41 – 7.40 (m, 4H), 7.15 – 7.10 (m, 8H), 7.09 – 7.01 (m, 24H), 4.84 – 4.76 (m, 16H), 4.09 – 3.98 (m, 64H), 3.40 (s, 4H), 3.39 (s, 4H), 1.91 – 1.75 (m, 80H), 1.59 – 1.44 (m, 64H), 1.43 – 1.23 (m, 164H), 1.18 – 1.05 (m, 48H), 0.96 – 0.82 (m, 96H), 0.74 – 0.67 (m, 24H).

MS (MALDI-TOF, DCTB): *m/z* (%) = 9026.0 (35) $[M+6DCTB]^+$, 8775.8 (54) $[M+5DCTB]^+$, 8525.6 (85) $[M+4DCTB]^+$, 8275.4 (100) $[M+3DCTB]^+$, 8025.2 (90) $[M+2DCTB]^+$, 7774.0 (53) $[M+DCTB]^+$, 7523.8 (11) $[M]^+$; calculated: 7518.85.

Analytical GPC (PS-calibrated in THF): $M_p = 11.5 \cdot 10^3$ g mol^{-1}.

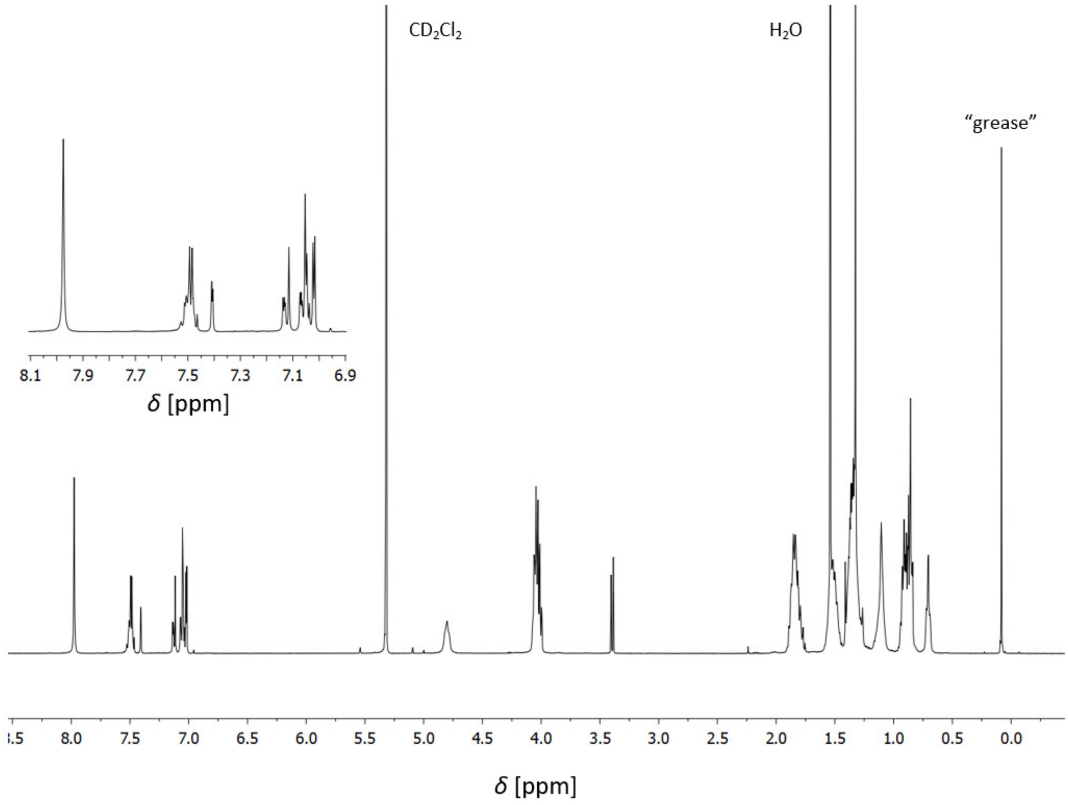

Supplementary Figure 25. ^{1}H-NMR spectrum of 11_4 in CD_2Cl_2.

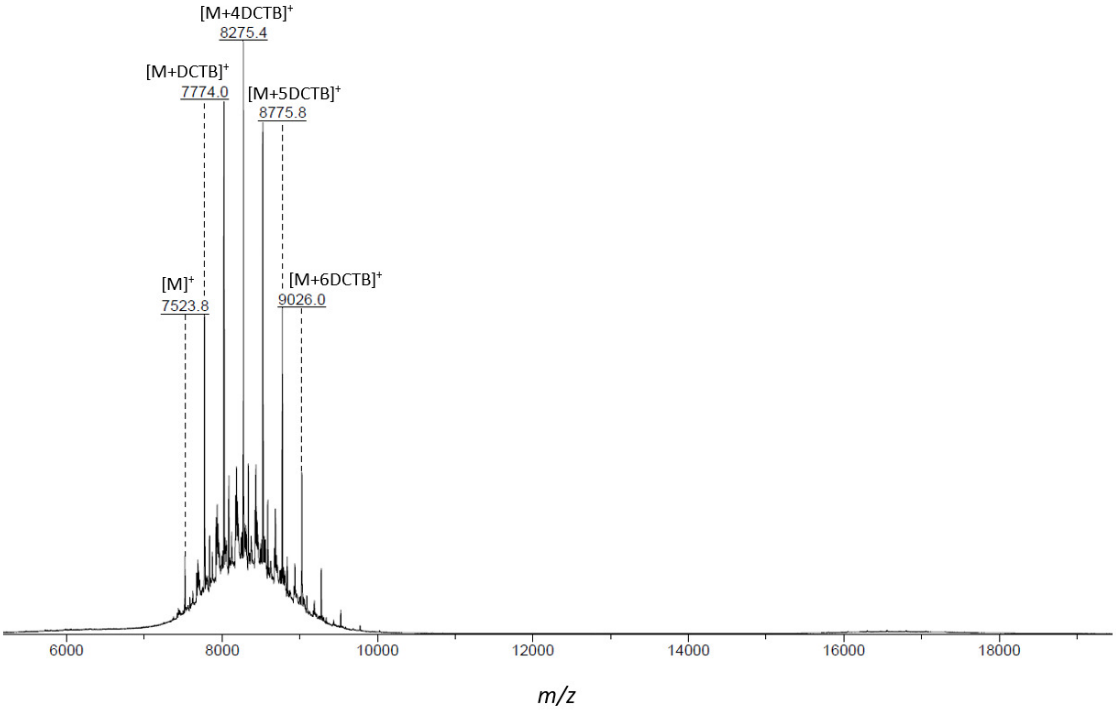

Supplementary Figure 26. MALDI-MS spectrum of 11_4. Matrix: DCTB.

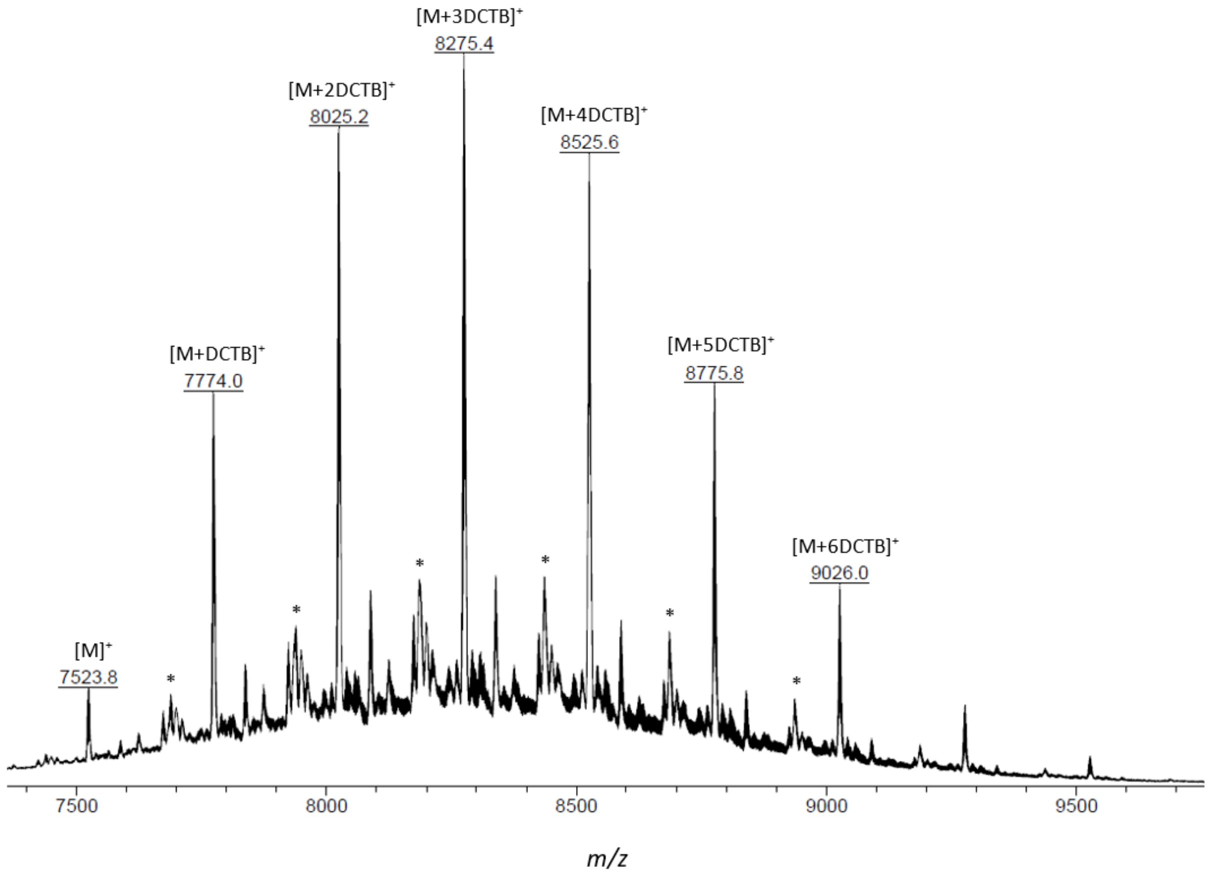

Supplementary Figure 27. Detail of MALDI-MS spectrum of 11_4. Matrix: DCTB; signals labelled with * are due to alkyl fragmentation -C_6H_{13}.

11_8

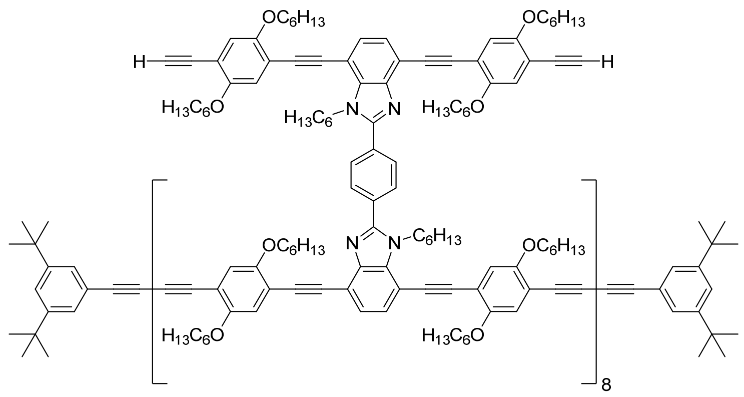

10_8 (4.3 mg, 245 nmol) was dissolved in dichloromethane (8 mL) and tetra-*n*-butylammonium fluoride (1 mL, 1 M in THF) was added. The reaction mixture was stirred at 35 °C for 3 h. Dichloromethane and water were added and the layers were separated. The aqueous layer was extracted with dichloromethane, the combined organic layers were washed with water, dried (Na_2SO_4) and the solvent was evaporated. The crude product was purified by recGPC. The product was obtained as a yellow film.

Due to the small amount of substance, the product was not further purified as usual by precipitation following recGPC. For this reason no exact weight was determined and no yield is given in this step.

Formula: $C_{992}H_{1226}N_{32}O_{64}$

Mol weight: 14622.88 g mol^{-1}.

MS (MALDI-TOF, DCTB): *m/z* (%) = 18380.1 (12) $[M+15DCTB]^+$, 18129.6 (19) $[M+14DCTB]^+$, 17878.4 (31) $[M+13DCTB]^+$, 17627.8 (43) $[M+12DCTB]^+$, 17377.0 (62) $[M+11DCTB]^+$, 17126.6 (81) $[M+10DCTB]^+$, 16875.9 (95) $[M+9DCTB]^+$, 16625.2 (100) $[M+8DCTB]^+$, 16374.8 (93) $[M+7DCTB]^+$, 16123.8 (75) $[M+6DCTB]^+$, 15873.5 (51) $[M+5DCTB]^+$, 15622.7 (25) $[M+4DCTB]^+$, 15372.3 (9) $[M+3DCTB]^+$, 15121.0 (3) $[M+2DCTB]^+$; calculated: 14611.37.

Analytical GPC (PS-calibrated in THF): $M_p = 24.7 \cdot 10^3$ g mol^{-1}.

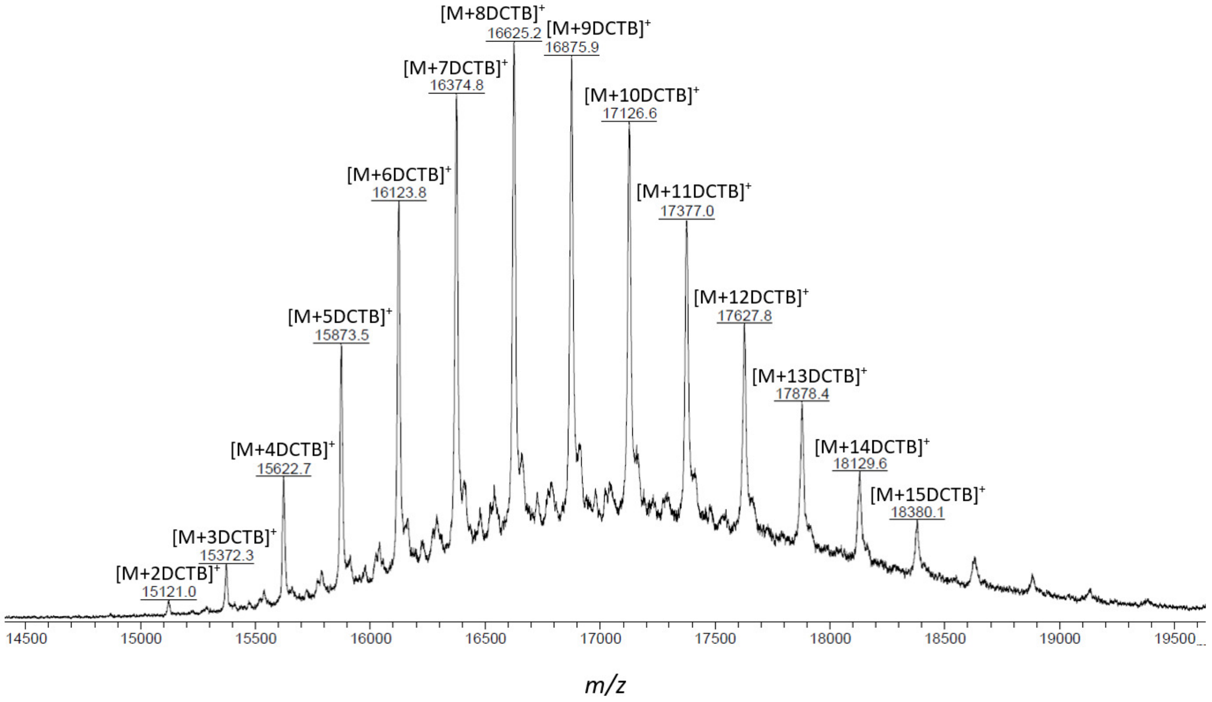

Supplementary Figure 28. Detail of MALDI-MS spectrum of 11_8. Matrix: DCTB.

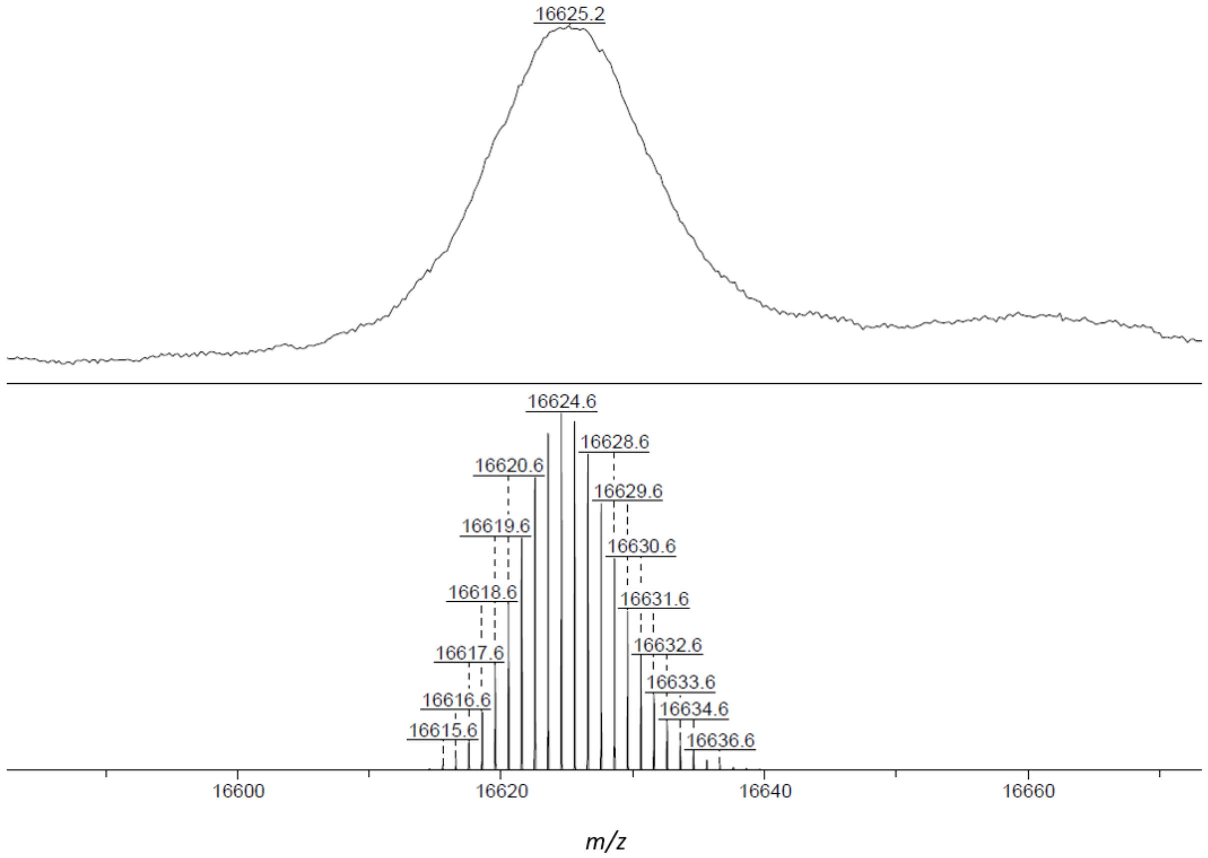

Supplementary Figure 29. Comparison of the observed (top) and calculated (bottom) isotopic signatures of the [M+8DCTB]$^+$ peak of the MALDI-MS spectrum of 11_8.

12_4

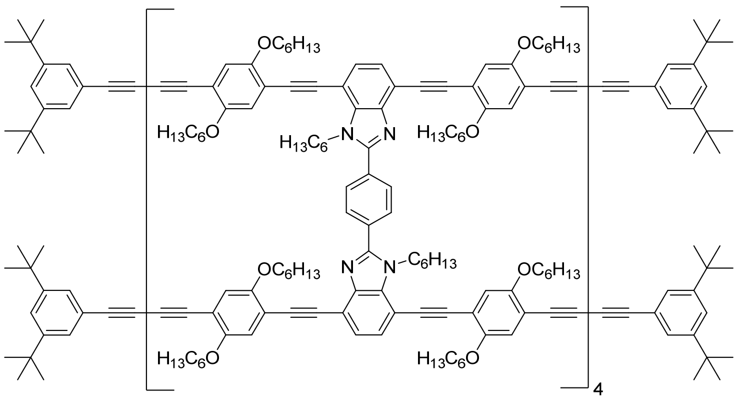

11_4 (crude product) was dissolved in pyridine (150 mL) and heated to 35 °C. CuCl (1.04 g, 10.5 mmol) and $CuCl_2$ (105 mg, 781 µmol) were added, and the suspension was stirred at 35 °C for 25 min. 3,5-Di-*tert*-butyl

phenylacetylene (459 mg, 2.14 mmol) was added and the reaction mixture was stirred at 35 °C for an additional 17 h. Thereafter, dichloromethane and aq. EDTA-solution (0.1 M) were added. The layers were separated and the aqueous layer was extracted with dichloromethane. The combined organic layers were washed with aq. HCl (10%), aq. EDTA-solution (0.1 M), and water, dried (Na_2SO_4), and the solvent was evaporated. The crude product was subjected to a short filter column (dichloromethane) and recGPC. The pre-purified product was diluted in dichloromethane and precipitated with methanol. After filtration, the product was obtained as a yellow solid (2.5 mg, 315 nmol, 59 % for two steps).

Formula: $C_{544}H_{668}N_{16}O_{32}$

Mol weight: 7943.41 g mol^{-1}.

^{1}H-NMR (700 MHz, CD_2Cl_2, r.t.): δ [ppm] = 8.00 – 7.93 (m, 16H), 7.53 – 7.47 (m, 20H), 7.42 – 7.40 (m, 8H), 7.18 – 7.12 (m, 8H), 7.11 – 7.04 (m, 24H), 4.88 – 4.72 (m, 16H), 4.10 – 4.02 (m, 64H), 1.91 – 1.75 (m, 80H), 1.59 – 1.49 (m, 64H), 1.44 – 1.25 (m, 200H), 1.16 – 1.00 (m, 48H), 0.96 – 0.90 (m, 48H), 0.90 – 0.82 (m, 48H), 0.73 – 0.68 (m, 24H).

MS (MALDI-TOF, Dithranol): *m/z* (%) = 8694.5 (3) [M+3DCTB]$^+$, 8444.5 (14) [M+2DCTB]$^+$, 8194.4 (42) [M+DCTB]$^+$, 7943.4 (100) [M]$^+$; calculated: 7937.11.

Analytical GPC (PS-calibrated in THF): M_p = 11.5·10^3 g mol^{-1}.

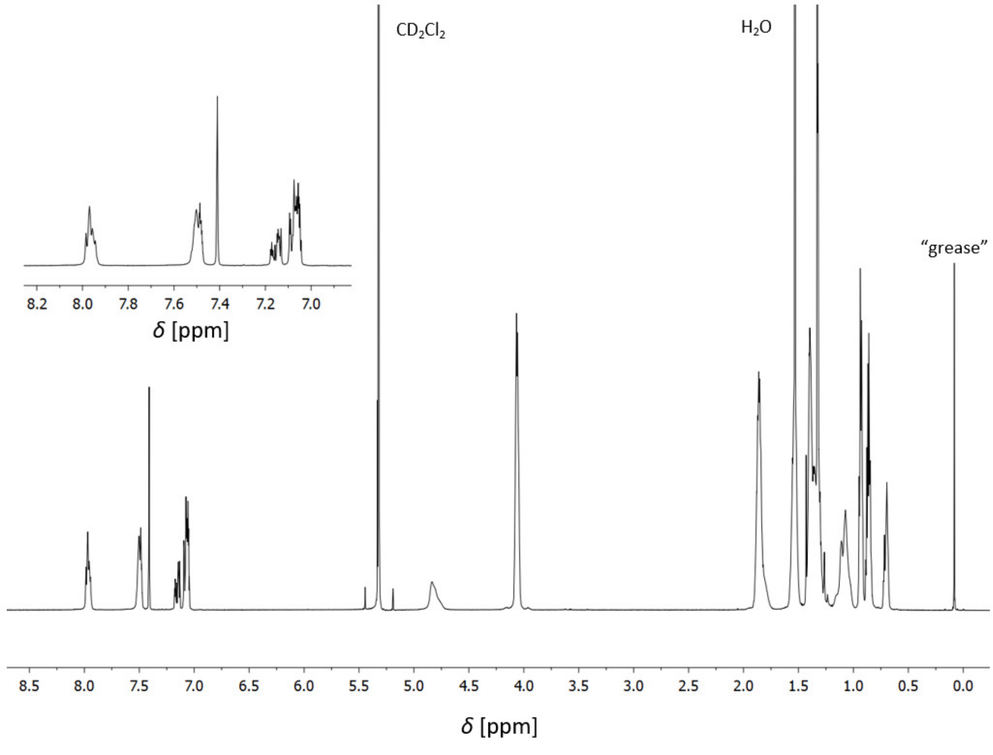

Supplementary Figure 30. ^{1}H-NMR spectrum of 12_4 in CD_2Cl_2.

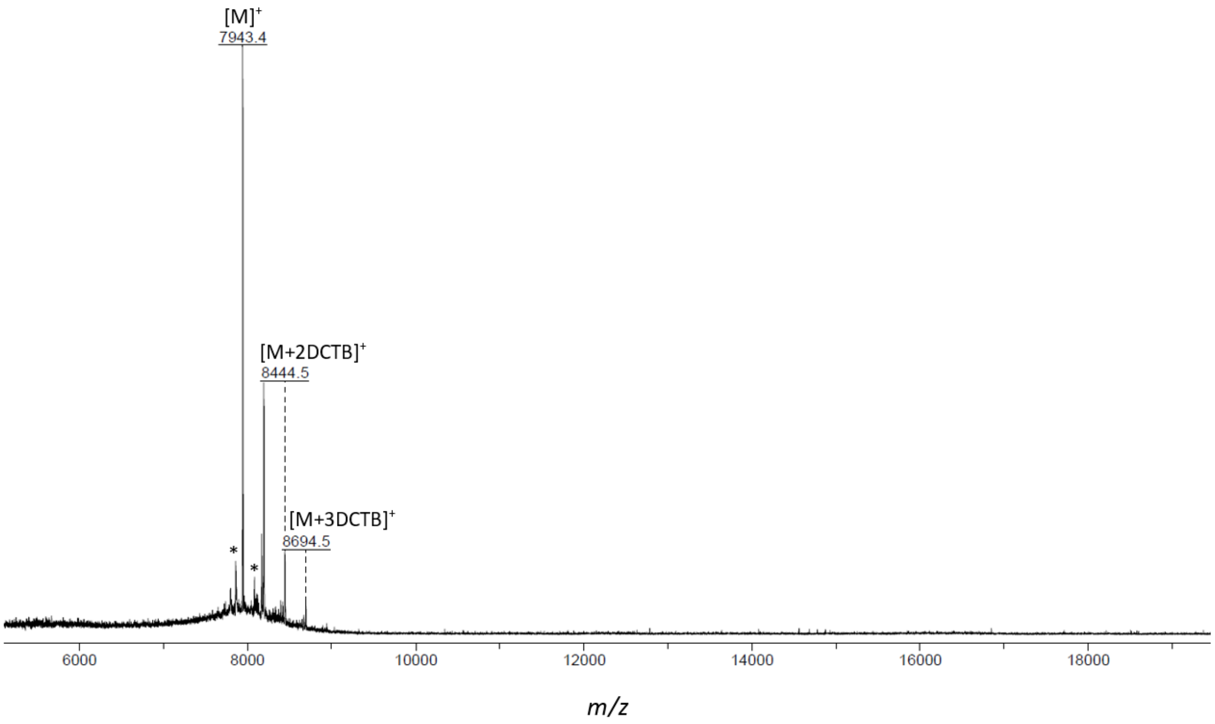

Supplementary Figure 31. MALDI-MS spectrum of 12_4. Matrix: DCTB; signals labelled with * are due to alkyl fragmentation -C_6H_{13}.

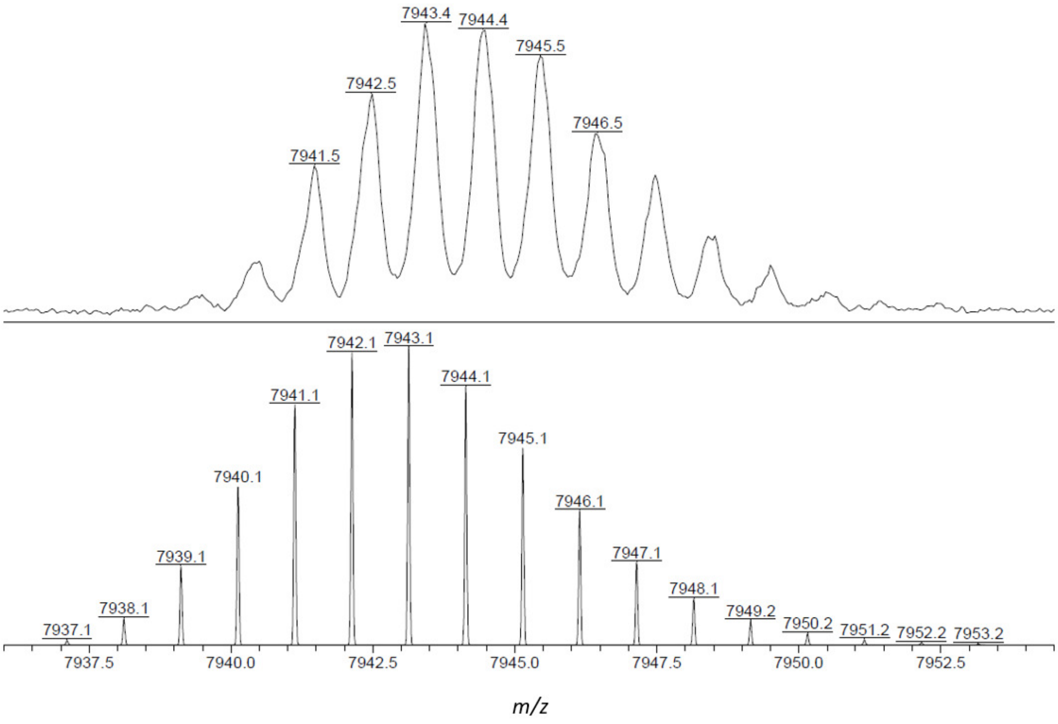

Supplementary Figure 32. Comparison of the observed (top) and calculated (bottom) isotopic signatures of the $[M]^+$ peak of the MALDI-MS spectrum of 12_4.

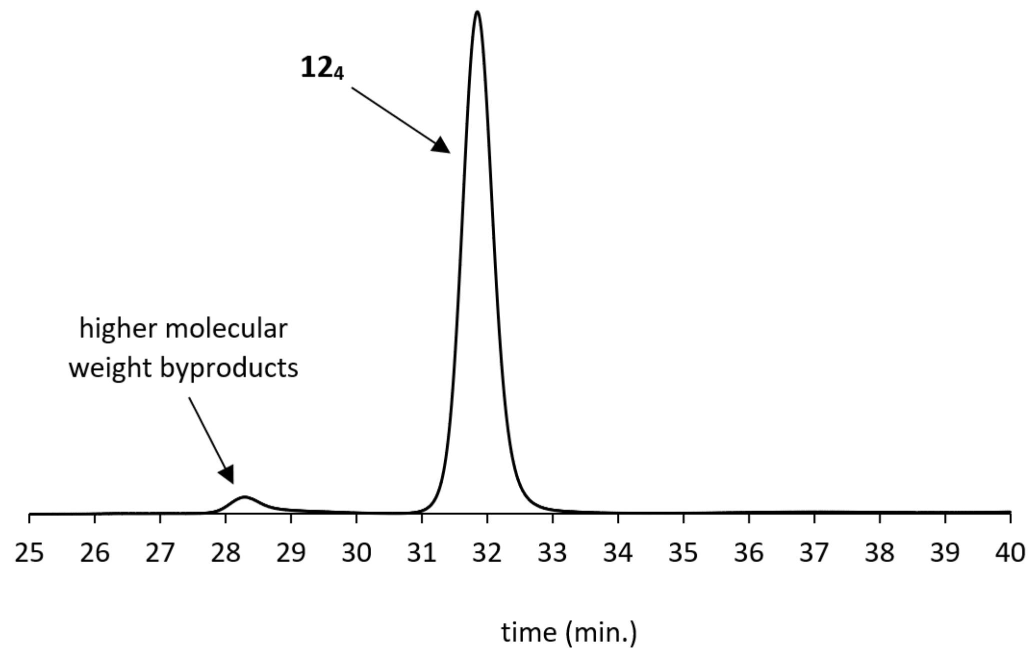

Supplementary Figure 33. **Recycling GPC elugram of the crude product of 12_4.**

12_8

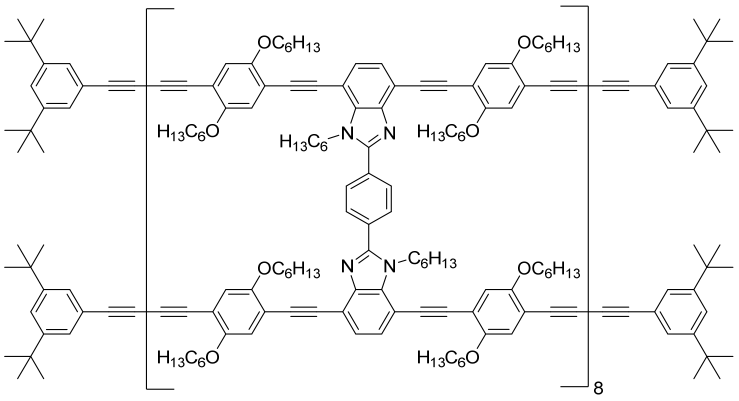

11_8 (crude product) was dissolved in pyridine (150 mL) and heated to 35 °C. CuCl (1.04 g, 10.5 mmol), and $CuCl_2$ (115 mg, 855 µmol) were added and the suspension was stirred at 35 °C for 40 min. 3,5-Di-*tert*-butyl phenylacetylene (495 mg, 2.31 mmol) was added and the reaction mixture was stirred for an additional 15 h at 35 °C. Thereafter, dichloromethane and aq. EDTA-solution (0.1 M) were added. The layers were separated and the aqueous layer was extracted with dichloromethane. The combined organic layers were washed with aq. HCl (10%), aq. EDTA-solution (0.1 M), and water, dried (Na_2SO_4), and the solvent was evaporated. The crude product was subjected to a short filter column (dichloromethane) and recGPC. The

pre-purified product was diluted in dichloromethane and precipitated with methanol. After filtration, the product was obtained as a yellow solid (2.0 mg, 133 nmol, 54% for two steps).

Formula: $C_{1024}H_{1252}N_{32}O_{64}$, molecular weight: 15033.44 g mol^{-1}.

^{1}H-NMR (700 MHz, CD_2Cl_2, r.t.): δ [ppm] = 8.00 – 7.93 (m, 32H), 7.56 – 7.46 (m, 36H), 7.42 – 7.40 (m, 8H), 7.21 – 7.13 (m, 16H), 7.12 – 7.03 (m, 48H), 4.90 – 4.72 (m, 32H), 4.13 – 4.00 (m, 128H), 1.92 – 1.76 (m, 160H), 1.60 – 1.48 (m, 128H), 1.46 – 1.25 (m, 328H), 1.19 – 0.99 (m, 96H), 0.97 – 0.90 (m, 96H), 0.90 – 0.82 (m, 96H), 0.74 – 0.66 (m, 48H).

MS (MALDI-TOF, Dithranol): *m/z* (%) = 7677.1 (32) [M+dithranol+Ag]$^{2+}$, 15021.3 (100) [M]$^+$; calculated: 15021.57.

Analytical GPC (PS-calibrated in THF): M_p = 24.8·10^3 g mol^{-1}.

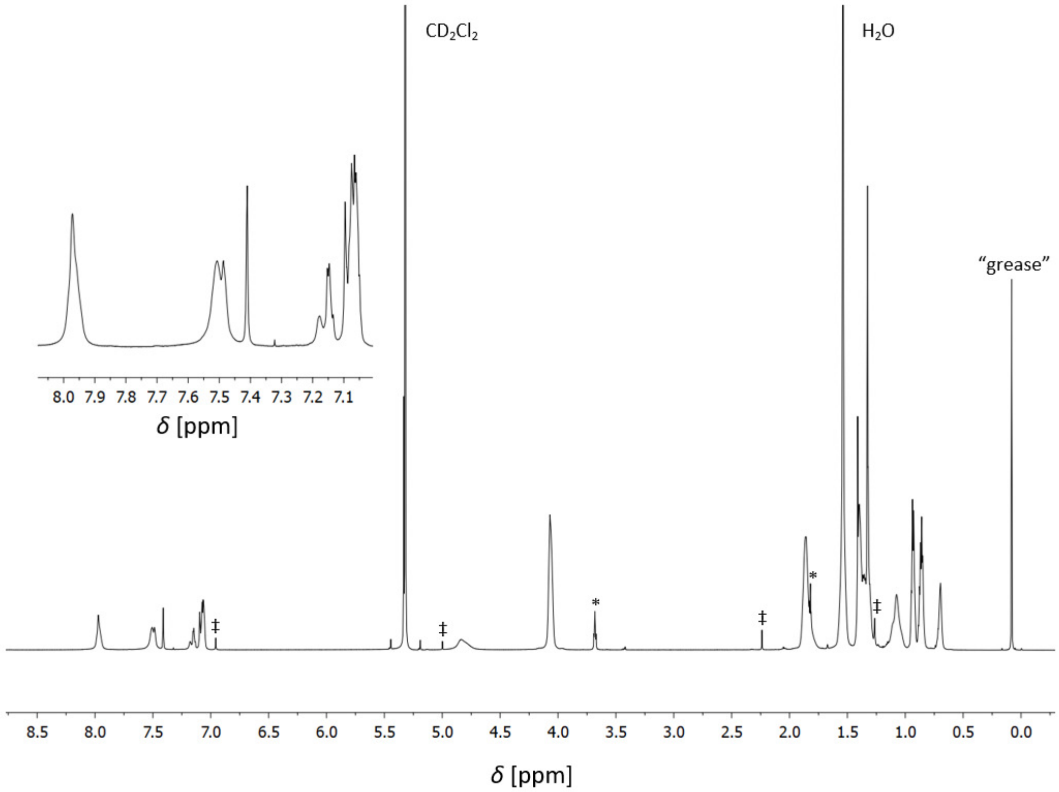

Supplementary Figure 34. ^{1}H-NMR spectrum of 12_8 in CD_2Cl_2. Signals labelled with ‡ are due to traces of BHT, signals labelled with * are due to traces of tetrahydrofuran.

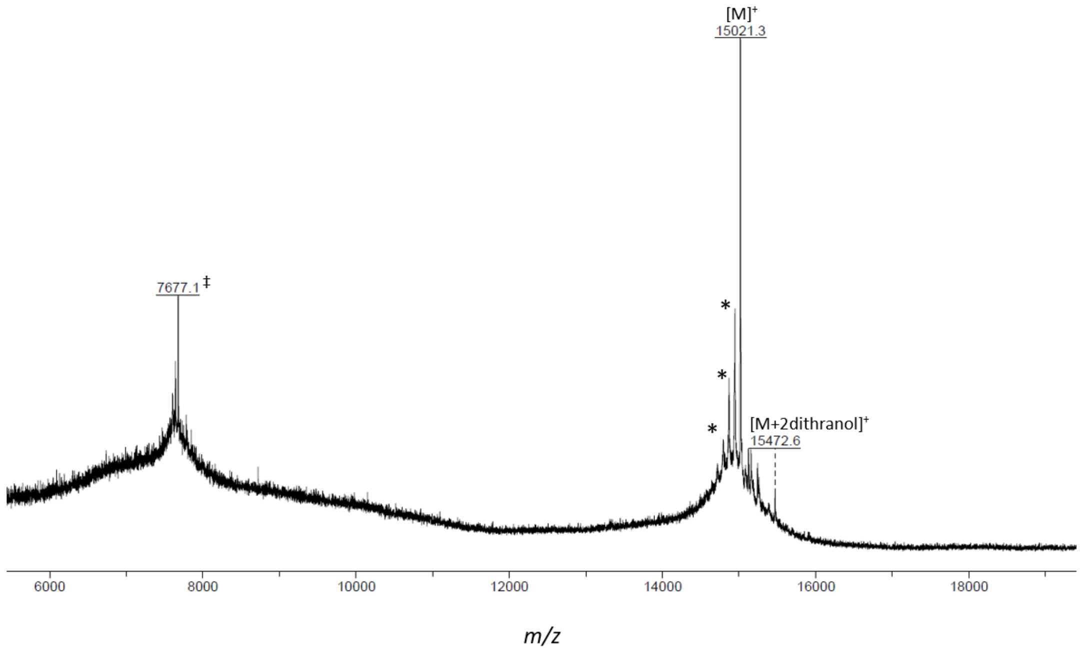

Supplementary Figure 35. MALDI-MS spectrum of 12_8 . Matrix: dithranol; signals labelled with ‡ are due to multiple charged adducts; signals labelled with * are due to alkyl fragmentation -C_6H_{13}.

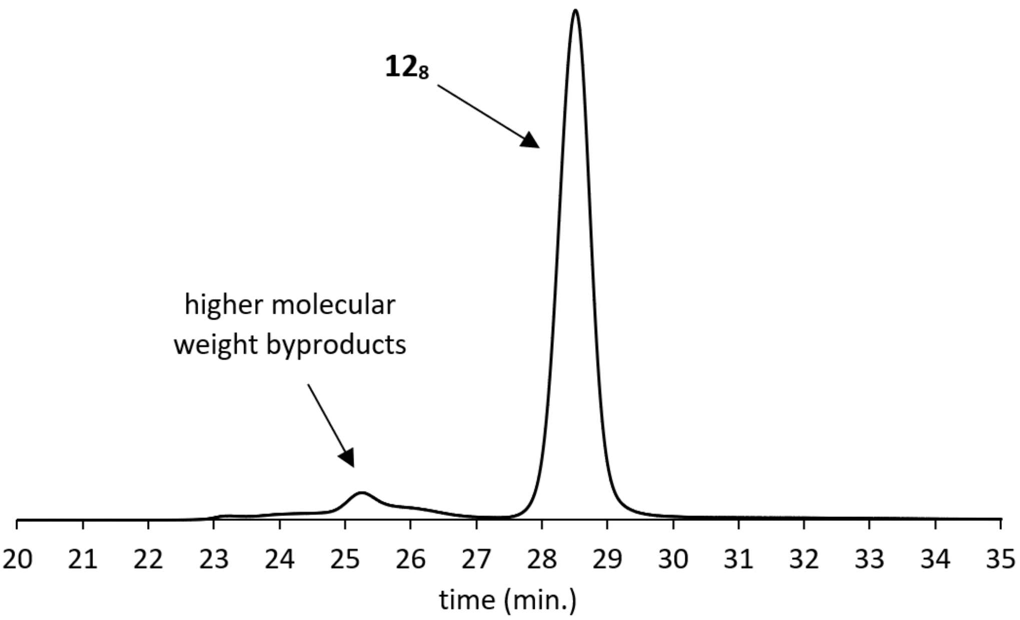

Supplementary Figure 36. **Recycling GPC elugram of the crude product of 12_8.**

Supplementary Note 4.4: Polymerization

10_n

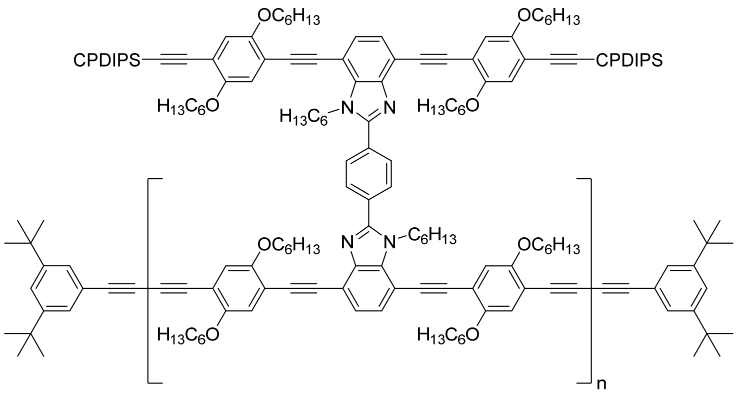

9 (351 mg, 164 µmol) was dissolved in dichloromethane (40 mL). Pyridine (40 mL), CuCl (154 mg, 1.56 mmol) and $CuCl_2$ (20 mg, 149 µmol) were added and the suspension was stirred at 25 °C for 16 h. 3,5-Di-*tert*-butyl phenylacetylene (329 mg, 1.53 mmol) was added and the reaction mixture was stirred at 25 °C for an additional 5 h. The reaction was diluted with aq. EDTA-solution (0.1 M) and dichloromethane. The layers were separated and the aqueous layer was extracted with dichloromethane. The combined organic layers were washed with aq. EDTA-solution (0.1 M), aq. HCl (10%), water and brine, dried (Na_2SO_4), and the solvent was evaporated. The crude product was diluted with dichloromethane, filtrated through a pad of silica gel and rinsed with dichloromethane. The solvent was removed under reduced pressure.

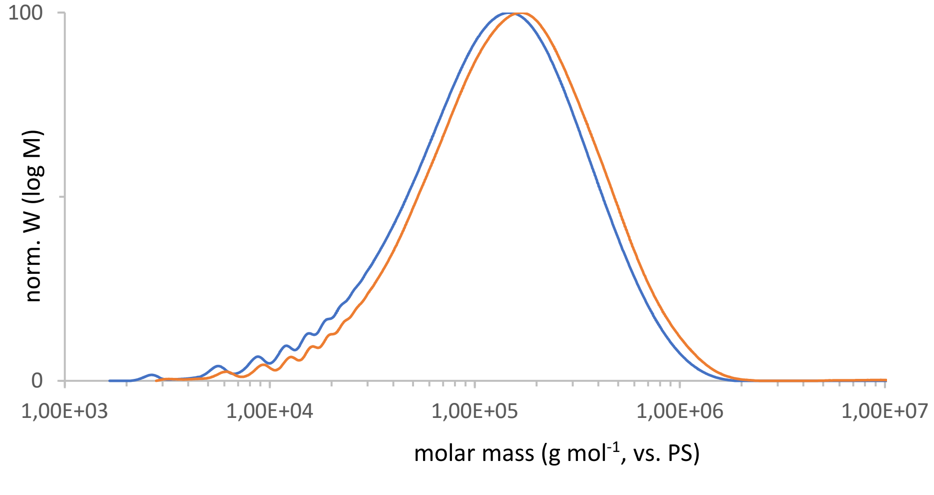

Supplementary Figure 37. Analytical GPC elugram after 16 h. $M_w = 191.3 \cdot 10^3$ g mol^{-1}, $M_n = 69.1 \cdot 10^3$ g mol^{-1}, $M_p = 149.7 \cdot 10^3$ g mol^{-1}, $PD = 2.8$ (blue). After 3,5-di-*tert*-butyl phenylacetylene addition and an additional 3 h to yield the endcapped polymer: $M_w = 225.3 \cdot 10^3$ g mol^{-1}, $M_n = 86.5 \cdot 10^3$ g mol^{-1}, $M_p = 171.3 \cdot 10^3$ g mol^{-1}, $PD = 2.6$ (orange).

The crude polymer was purified coarsely by recGPC and separated into five fractions with low polydispersity. One of the fractions was dissolved in dichloromethane and precipitated with methanol. After filtration, the polymer fraction was obtained as a yellow film.

Formula: $C_{140}H_{186}N_6O_8Si_2$ (repeating unit), $C_{32}H_{42}$ (endcap units), $C_{5632}H_{7482}N_{240}O_{320}Si_{80}$ (n = 40)

Mol weight: 2137.23 g mol^{-1} (repeating unit), 426.69 g mol^{-1} (endcap units), 85915.97 g mol^{-1} (n = 40).

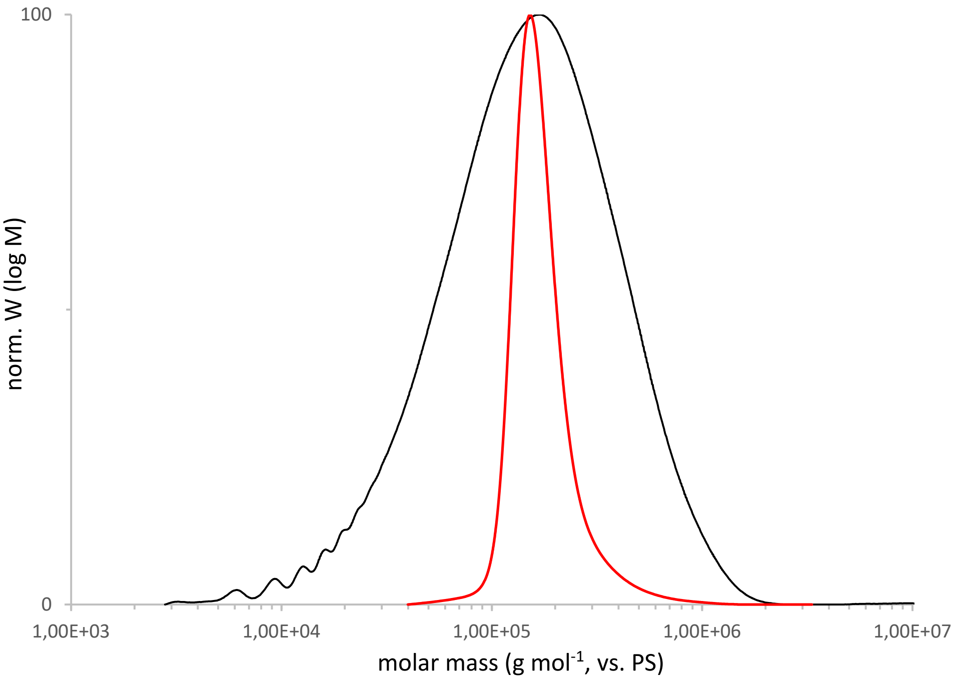

Supplementary Figure 38. Analytical GPC elugrams of the endcapped polymer and the isolated fraction (red).

10_n (approximately 10 mg, 2.8 %):

^{1}H-NMR (700 MHz, CD_2Cl_2, r.t.): δ [ppm] = 7.98 (s, 4H), 7.53 – 7.46 (m, 4H), 7.41 – 7.39 (m, 0.03H), 7.15 – 6.97 (m, 8H), 4.80 (s, 4H), 4.09 – 3.96 (m, 16H), 2.44 – 2.41 (m, 4H), 1.91 – 1.73 (m, 24H), 1.59 – 1.44 (m, 16H), 1.42 – 1.22 (m, 32H), 1.17 – 1.01 (m, 40H), 0.96 – 0.78 (m, 28H), 0.73 – 0.69 (m, 6H).

MS (MALDI-TOF, DCTB): *m/z* (%) see Supplementary Fig. 38; calculated: 2135.38 (repeating unit), 426.33 (endcap units), 85841.81 (n = 40).

Analytical GPC (PS-calibrated in THF): $M_n = 162.3 \cdot 10^3$ g mol^{-1}, $M_w = 183.4 \cdot 10^3$ g mol^{-1}, $M_p = 151.8 \cdot 10^3$ g mol^{-1}, *PD* = 1.1.

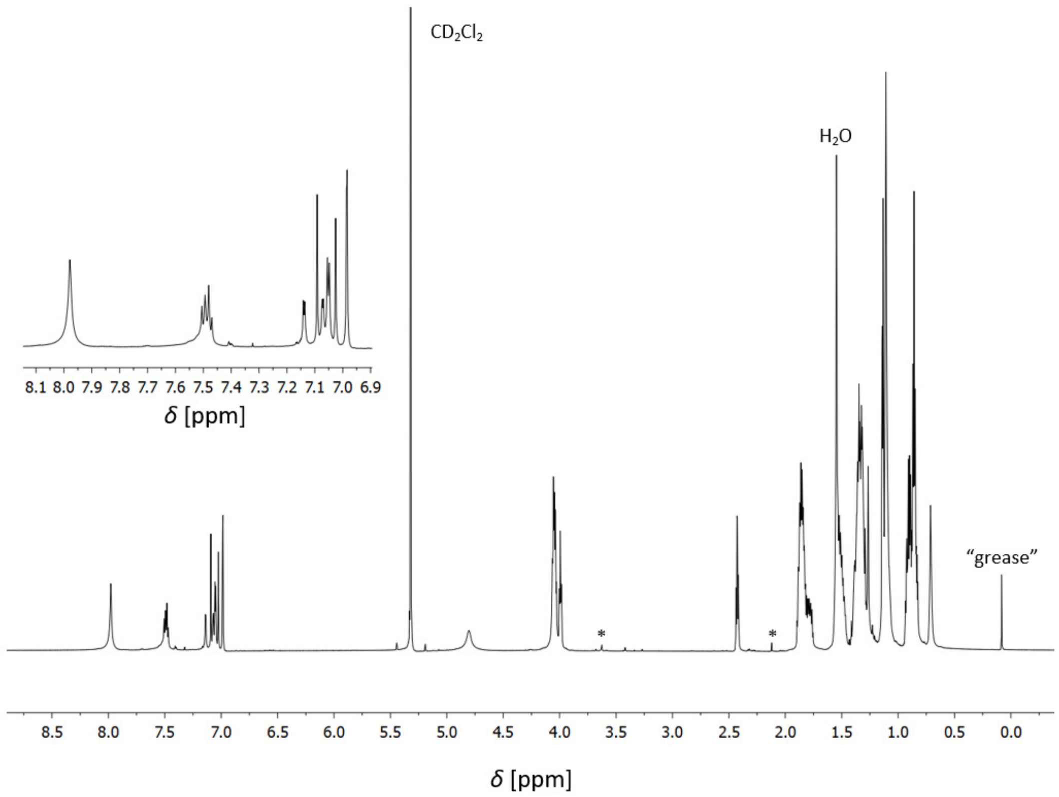

Supplementary Figure 39. ^{1}H-NMR spectrum of 10_n in CD_2Cl_2. Signals labelled with an asterisk are due to traces of tetrahydrofuran.

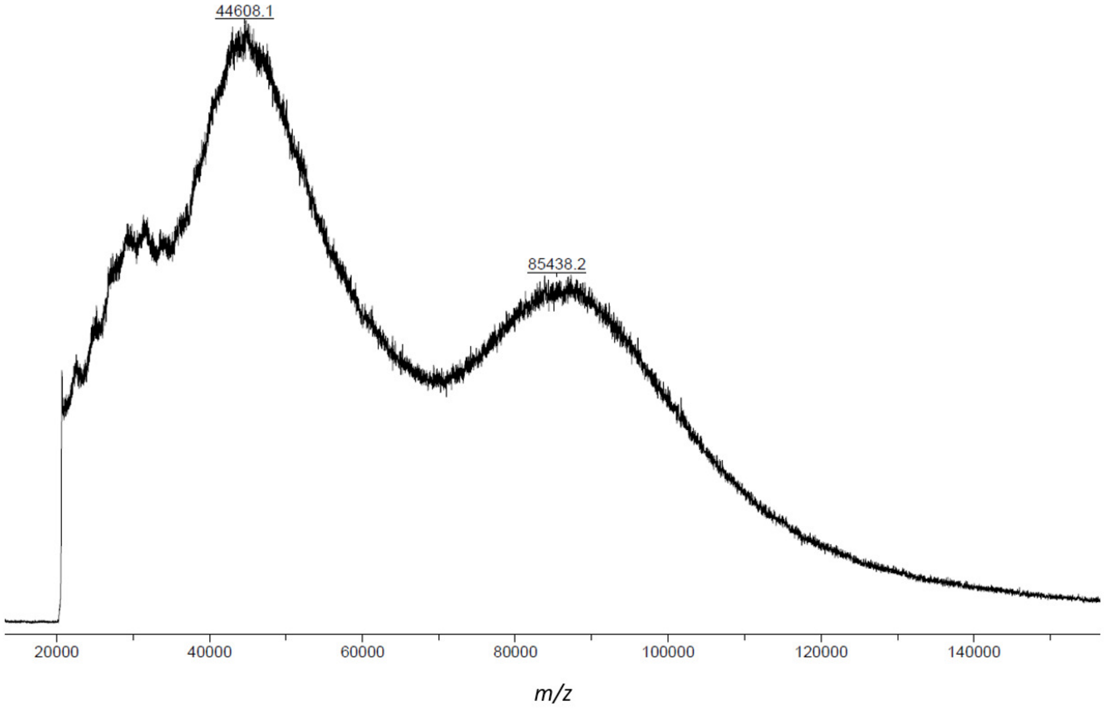

Supplementary Figure 40. MALDI-MS spectrum of 10_n. Matrix: DCTB.

The peak molecular weight of the GPC analysis M_p = 151.8·10^3 g mol^{-1} can be used to calculate the real molecular weight when divided by 1.78, the overestimation factor extrapolated for a degree of polymerization of 40. This gives the actual peak molecular weight of approximately 85.3 ·10^3 g mol^{-1} which agrees well the MALDI-TOF results.

11$_n$

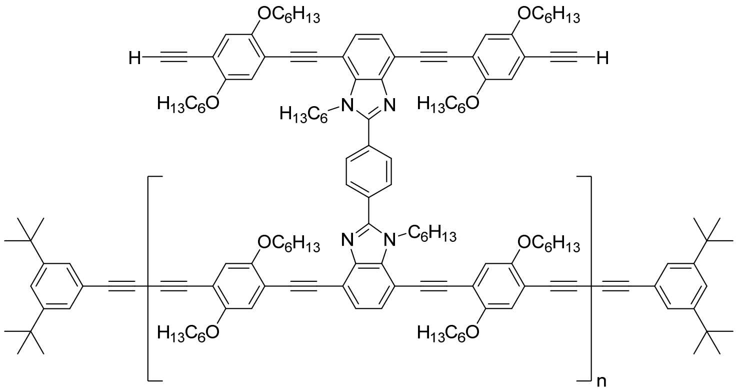

10$_n$ (approximately 10 mg) was dissolved in dichloromethane (10 mL) and tetra-*n*-butylammonium fluoride (1 mL, 1M in THF) was added. The reaction mixture was stirred at 35 °C for 1 h. Dichloromethane and water were added, and the layers were separated. The aqueous layer was extracted with dichloromethane, the combined organic layers were washed with water, dried (Na_2SO_4) and the solvent was evaporated. The crude product was purified *via* recGPC. The product was obtained as a yellow film.

Due to the small amount of substance, the product was not further purified as usual by precipitation following recGPC. For this reason no exact weight was determined and no yield is given in this step.

Formula: $C_{120}H_{148}N_4O_8$ (repeating unit), $C_{32}H_{42}$ (endcap units), $C_{4832}H_{5962}N_{160}O_{320}$ (n = 40).

Mol weight: 1774,5240 g mol^{-1} (repeating unit), 426.6880 g mol^{-1} (endcap units), 71407.648 g mol^{-1} (n = 40).

^{1}H-NMR (700 MHz, CD_2Cl_2, r.t.): δ [ppm] = 7.98 (s, 4H), 7.54 – 7.45 (m, 4H), 7.41 – 7.40 (m, 0.05H), 7.15 – 7.01 (m, 8H), 4.80 (s, 4H), 4.11 – 3.96 (m, 16H), 3.40 (s, 1H), 3.39 (s, 1H), 1.91 – 1.76 (m, 20H), 1.58 – 1.44 (m, 16H), 1.42 – 1.24 (m, 32H), 1.17 – 1.04 (m, 12H), 0.96 – 0.82 (m, 24H), 0.74 – 0.67 (m, 6H).

Analytical GPC (PS-calibrated in THF): M_n = 182.3·10^3 g mol^{-1}, M_w = 275.2·10^3 g mol^{-1}, M_p = 145.6·10^3 g mol^{-1}, *PD* = 1.5.

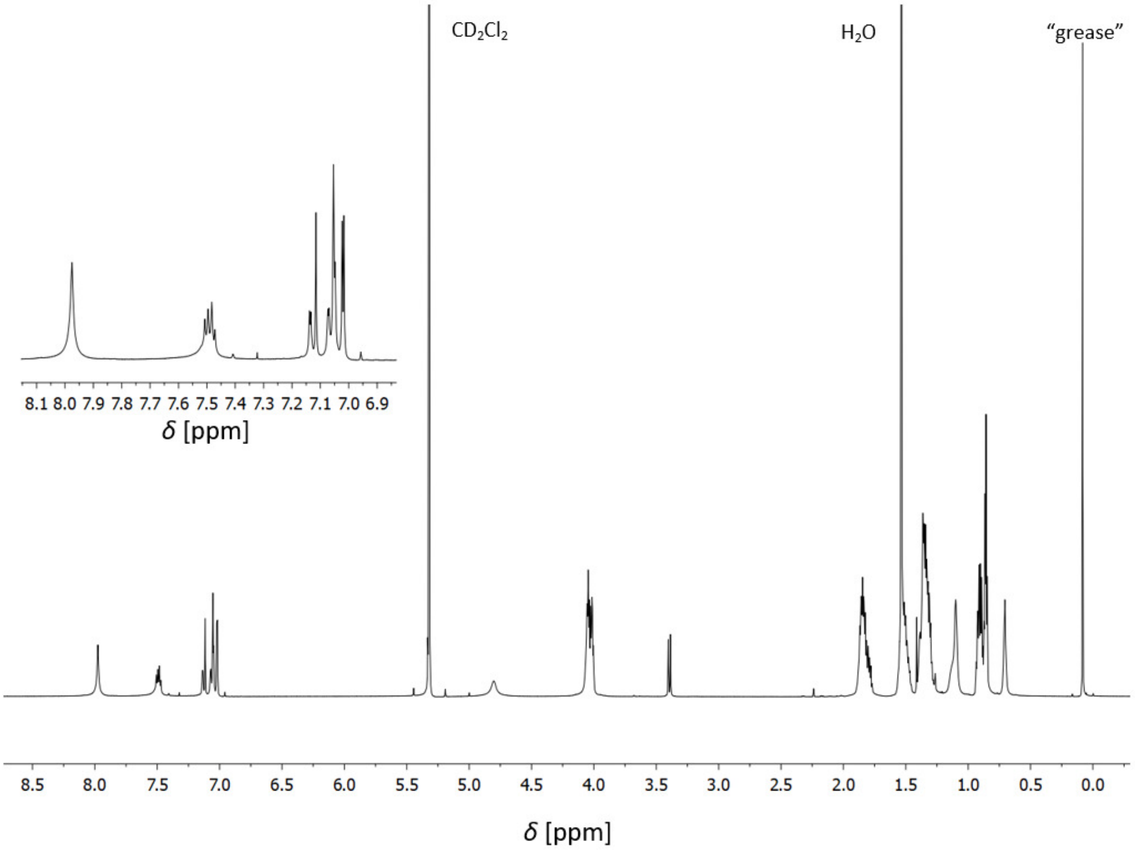

Supplementary Figure 41. ^{1}H-NMR spectrum of 11_n in CD_2Cl_2.

12_n

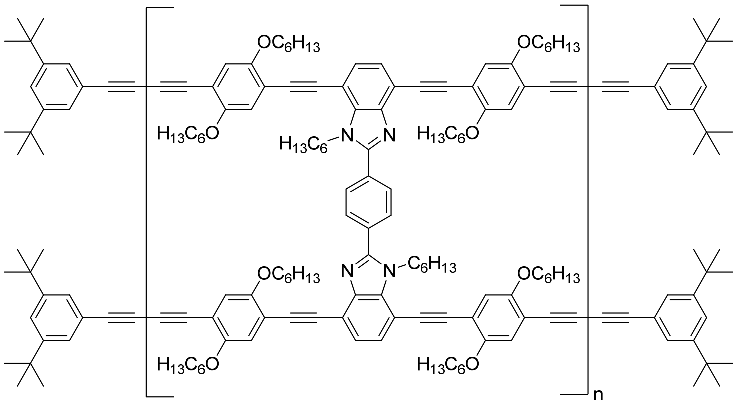

11_n (crude product) was dissolved in pyridine (150 mL) and heated to 35 °C. CuCl (1.02 g, 10.3 mmol), and $CuCl_2$ (134 mg, 997 µmol) were added and the suspension was stirred at 35 °C for 45 min. 3,5-Di-*tert*-butyl phenylacetylene (409 mg, 1.91 mmol) was added and the reaction mixture was stirred at 35 °C for an

additional 16 h. Thereafter, dichloromethane and aq. EDTA-solution (0.1 M) were added. The layers were separated and the aqueous layer was extracted with dichloromethane. The combined organic layers were washed with aq. HCl (10%), aq. EDTA-solution (0.1 M) and water, dried (Na_2SO_4), and the solvent was evaporated. The crude product was purified by filtering through a short filter column (dichloromethane) and by subsequent recGPC. The prepurified product was diluted in dichloromethane and precipitated with methanol. After filtration, the product was obtained as a yellow solid (1.9 mg, 23 %).

Formula: $C_{120}H_{146}N_4O_8$ (repeating unit), $C_{64}H_{84}$ (endcap units), $C_{4864}H_{5924}N_{160}O_{320}$ (n = 40).

Mol weight: 1772.51 g mol^{-1} (repeating unit), 853.38 g mol^{-1} (endcap units), 71753.70 g mol^{-1} (n = 40).

Due to the small amount of product, no ^{1}H-NMR spectrum could be obtained.

Analytical GPC (PS-calibrated in THF): M_n = 192.2·10^3 g mol^{-1}, M_w = 254.3·10^3 g mol^{-1}, M_p = 174.9·10^3 g mol^{-1}, *PD* = 1.3.

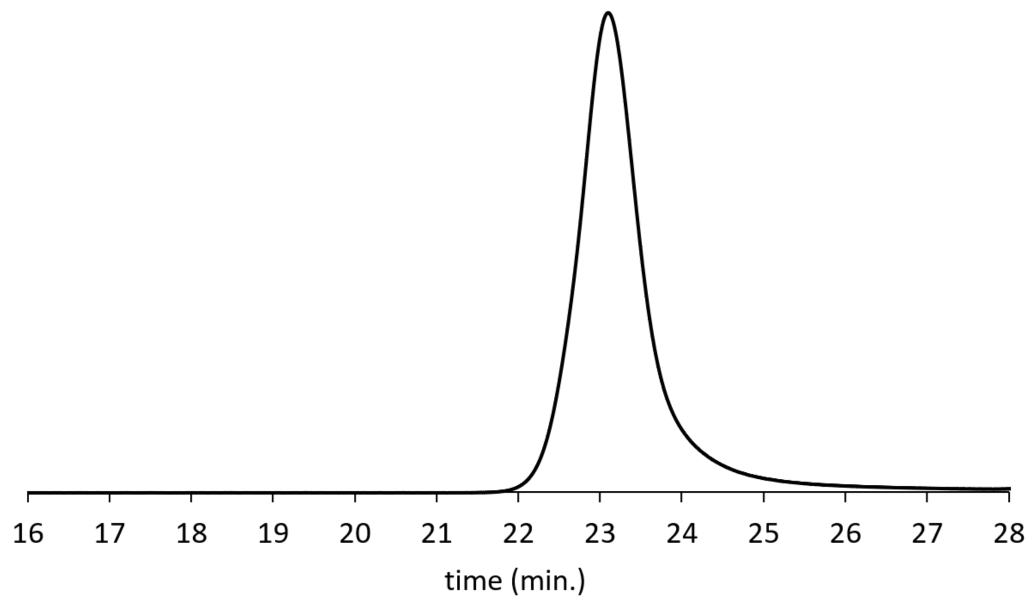

Supplementary Figure 42. **Recycling GPC elugram of the crude product of 12_n.**

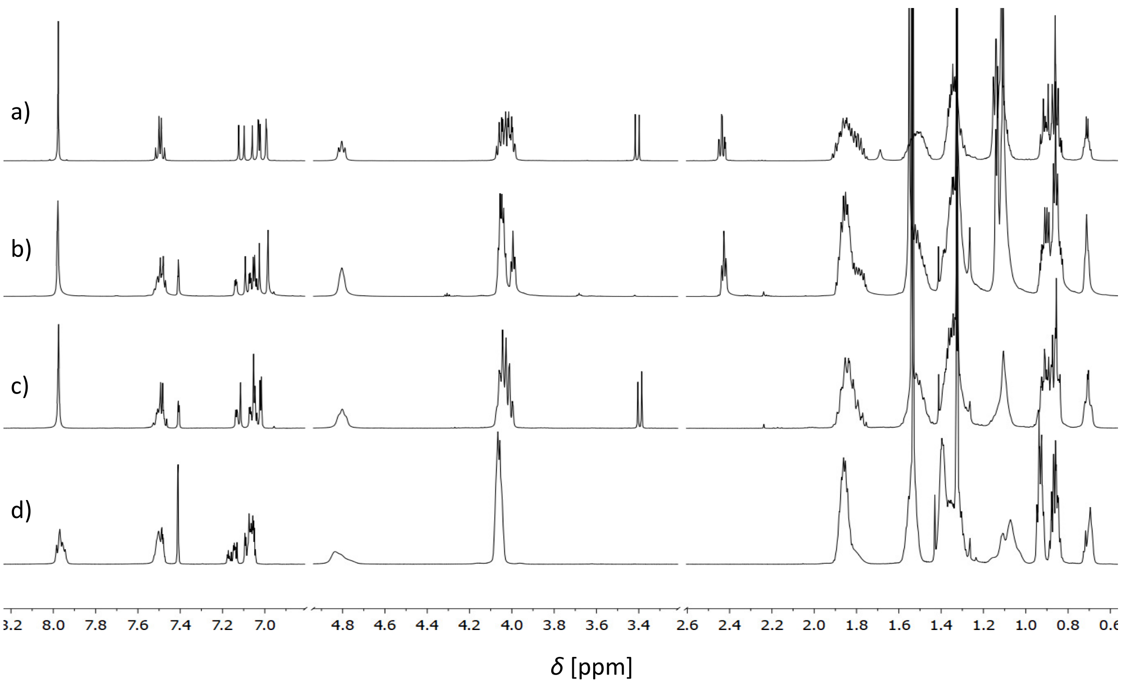

Supplementary Figure 43. Comparison of the ^{1}H-NMR spectra of the monomer 9 (a), open tetramer 10_4 (b), deprotected open tetramer 11_4 (c) and ladder tetramer 12_4 (d).

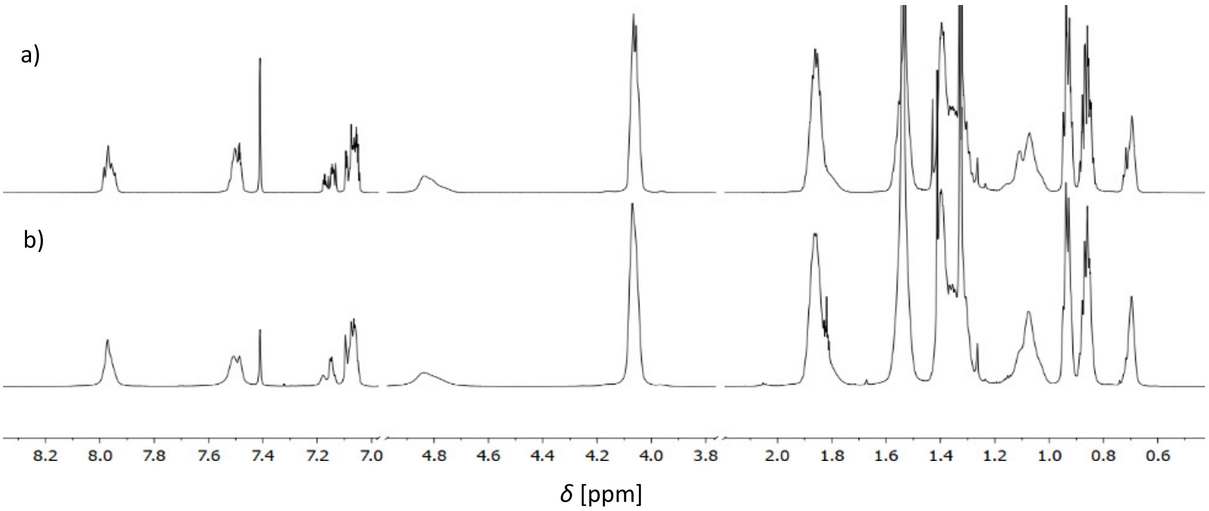

Supplementary Figure 44. Comparison of the ^{1}H-NMR spectra of the ladder tetramer 12_4 (a) and ladder octamer 12_8 (b).

Supplementary References

1 Lakowicz, J. R. Principles of Fluorescence Spectroscopy. *Kluwer Academic/Plenum: New York 3rd ed.* (2006).

2 Higgins, D. A. *et al.* Polarization-modulation near-field scanning optical microscopy of mesostructured materials. *J. Phys. Chem.* **100**, 13794-13803 (1996).

3 Adachi, T. *et al.* Highly ordered single conjugated polymer chain rod morphologies. *J. Phys. Chem. C* **114**, 20896-20902 (2010).

4 Hanbury Brown, R. *et al.* Correlation between Photons in two Coherent Beams of Light. *Nature* **177**, 27-28 (1956).

5 Weston, K. D. *et al.* Measuring the number of independent emitters in single-molecule fluorescence images and trajectories using coincident photons. *Anal. Chem.* **74**, 5342-5349 (2002).

6 Steiner, F. *et al.* Singlet-triplet annihilation limits exciton yield in poly(3-hexylthiophene). *Phys. Rev. Lett.* **112**, 137402 (2014).

7 Spicher, S. *et al*. Robust Atomistic Modeling of Materials, Organometallic, and Biochemical Systems. *Angew. Chem. Int. Ed.* **59**, 15665–15673 (2020).

8 "Semiempirical Extended Tight-Binding Program Package xtb", https://github.com/grimme-lab/xtb. Accessed: 2020-04-20.

9 Grimme, S. *et al.* A robust and accurate tight-binding quantum chemical method for structures, vibrational frequencies, and noncovalent interactions of large molecular systems parametrized for all spd-block elements (Z=1–86). *J. Chem. Theory Comput.* **13**, 1989–2009 (2017).

10 Bannwarth, C. *et al.* GFN2-xTB - An accurate and broadly parametrized self-consistent tight-binding quantum chemical method with multipole electrostatics and density-dependent dispersion contributions. *J. Chem. Theory Comput.* **15**, 1652–1671 (2019).

11 Brehm, M. *et al.* TRAVIS - A Free Analyzer and Visualizer for Monte Carlo and Molecular Dynamics Trajectories. *J. Chem. Inf. Model.* **51**, 2007–2023, PMID: 21761915 (2011).

12 Jester, S.-S. *et al.* Oligomers and Cyclooligomers of Rigid Phenylene–Ethynylene–Butadiynylenes: Synthesis and Self-Assembled Monolayers. *Angew. Chem. Int. Ed.* **49**, 6101–6105 (2010).

13 Yang, T. *et al.* Self-assembly of long chain alkanes and their derivatives on graphite. *J. Chem. Phys.* **128**, 124709 (2009).

14 Hündgen, M. *et al.* Supramolecular nanopatterns of H-shaped molecules. *Chem. Commun.* **54**, 10558–10561 (2018).

15 Jester, S.-S. *et al.* Self-assembled monolayers of clamped oligo(phenylene-ethynylene-butadiynylene)s. *Chem. Commun.* **47**, 8838–8840 (2011).

16 Lei, S.-B. *et al.* Substituent Effects on Two-Dimensional Assembling and Chain Folding of Rigid-Rod Polymer Poly(p-phenyleneethynylene) Derivatives on the Solid/Liquid Interface. *Macromolecules* **40**, 4552-4560 (2007).

17 Samorí, P. *et al.* Macromolecular Fractionation of Rod-Like Polymers at Atomically Flat Solid-Liquid Interfaces. *Adv. Mater.* **12**, 579-582 (2000).

18 Gampe, D. M. *et al.* Pushing to the low limits: tetraazaanthracenes with very low-lying LUMO levels and near-infrared absorption. *Chem. Commun.*, **53**, 10220–10223 (2017).

19 Shimada, M. *et al.* Bright Solid State Emission of Disilane-Bridged Donor-Acceptor-Donor and Acceptor-Donor-Acceptor Chromophores. *Angew. Chem. Int. Ed.* **55**, 3022–3026 (2016).

20 Gaefke, G. *et al.* [(3-Cyanopropyl) diisopropylsilyl] acetylene, a More Stable Analogue of [(3-Cyanopropyl) dimethylsilyl] acetylene. *Synthesis* **14**, 2155–2157 (2008).

21 Wang, C. *et al.* Convergent synthesis of 10 nm aryleneethynylene molecular wires by an iterative regioselective deprotection/sonogashira coupling protocol. *J. Org. Chem.* **71**, 108–116 (2006).

22 Zhang, R. et al. Soluble Silver Acetylide for the Construction and Structural Conversion of All-Alkynyl-Stabilized High-Nuclearity Homoleptic Silver Clusters. *Cryst. Growth Des.* **15**, 2505–2513 (2015).

23 Hinderer, F. *et al.* Monodisperse (p-Phenylene-Butadiynylene)s: GPC Conversion Factors and Self-Assembled Monolayers. *Macromolecules* **49**, 1816–1821 (2016).
