# Supplementary material for: Nanoscale π-conjugated ladders
Source: Nat Commun. 2021 Nov 16;12:6614. doi: 10.1038/s41467-021-26688-9 (PMC8595307; doi:10.1038/s41467-021-26688-9)
Supplement: Supplementary file 2 — Description of Additional Supplementary Files [file 41467_2021_26688_MOESM2_ESM.pdf]

Description of Additional Supplementary Files

File Name: Supplementary Movie 1
Description: Visualization of a 100 ps segment of the GFN-FF/GBSA(THF) MD simulation trajectory of the open octamer (10₈) after the equilibration phase. The structural change from a straight to a disordered conformation is captured.

File Name: Supplementary Movie 2
Description: Visualization of a 100 ps segment from the GFN-FF/GBSA(THF) MD simulation trajectory of the closed octamer (12₈) after the equilibration phase. The structure remains elongated throughout the entire simulation.
